# Naturalized and Invasive Species Integrate Differently in the Trait Space of Local Plant Communities

**DOI:** 10.1111/ele.70235

**Published:** 2025-11-09

**Authors:** Jan Divíšek, Petr Pyšek, David M. Richardson, Nicholas J. Gotelli, Brian Beckage, Jane Molofsky, Zdeňka Lososová, Milan Chytrý

**Affiliations:** ^1^ Department of Botany and Zoology, Faculty of Science Masaryk University Brno Czech Republic; ^2^ Department of Geography, Faculty of Science Masaryk University Brno Czech Republic; ^3^ Department of Invasion Ecology, Institute of Botany Czech Academy of Sciences Průhonice Czech Republic; ^4^ Department of Ecology, Faculty of Science Charles University Prague Czech Republic; ^5^ Centre for Invasion Biology, Department of Botany and Zoology Stellenbosch University Stellenbosch South Africa; ^6^ Department of Biology University of Vermont Burlington Vermont USA; ^7^ Department of Plant Biology University of Vermont Burlington Vermont USA; ^8^ Department of Computer Science University of Vermont Burlington Vermont USA; ^9^ Gund Institute for Environment University of Vermont Burlington Vermont USA

**Keywords:** community invasibility, functional traits, habitat types, invasion stages, plant communities, species invasiveness, vegetation

## Abstract

How alien plant species integrate into local native communities remains a widely debated but largely unresolved question. For 12,460 plant communities from six different habitats, we show that naturalized non‐invasive species integrate near the center of the multidimensional functional trait space of each community, whereas invasive species tend to occupy the edges. This pattern is driven mainly by specific leaf area, plant height and seed mass, followed by genome size. These results suggest that functional similarity to resident native species supports successful naturalization of alien species through preadaptation to environmental conditions. In contrast, the functional dissimilarity of invasive species enables them to exploit new niches, potentially avoiding direct competition with co‐occurring native species while still passing through environmental filters. The magnitude of differences between native, naturalized and invasive species is habitat‐specific, reflecting both the local ecological conditions and the traits of the most widespread species in a given habitat.

## Graphical abstract

We investigated the relative positions of native, non‐invasive naturalized and invasive plant species in the functional trait space of local communities in six different habitat types. We show that naturalized species integrate near the center of the multidimensional functional trait space of each community, whereas invasive species tend to occupy the edges.

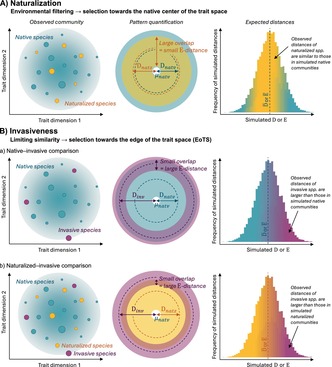

## 1 Introduction

The introduction‐naturalization‐invasion (INI) continuum (Richardson et al. 2000) separates alien species based on the invasion stage they have achieved into casual, naturalized and invasive. Naturalized species maintain self‐replacing populations over multiple life cycles without direct human intervention, while invasive species, a subset of naturalized species, produce a large number of offspring, dominate in invaded communities and spread over long distances. Understanding the mechanisms underlying the transition of species along this continuum is a key challenge in invasion ecology (Richardson and Pyšek 2012).

Two opposing hypotheses were proposed to explain the successful naturalization in a community of native species and species advancement to the invasive stage. The environmental filtering hypothesis (Keddy 1992) states that alien species functionally similar to native ones are more likely to establish and persist in a community because they fit local environmental conditions. In contrast, the limiting similarity hypothesis (MacArthur and Levins 1967) posits that alien species must be sufficiently different from native species to reduce niche overlap and competition. Numerous studies have compared traits of naturalized (non‐invasive) versus invasive species (e.g., Gallagher et al. 2015; van Kleunen et al. 2010), demonstrating that certain traits of invasive species, such as larger specific leaf area (SLA), earlier and longer flowering, or tall stature, tend to differ from those of naturalized species (Gallagher et al. 2015; Pyšek et al. 2009). Moreover, invasive species tend to exhibit greater trait differentiation from native than naturalized species do (Divíšek et al. 2018; van Kleunen et al. 2010).

**FIGURE 1:**
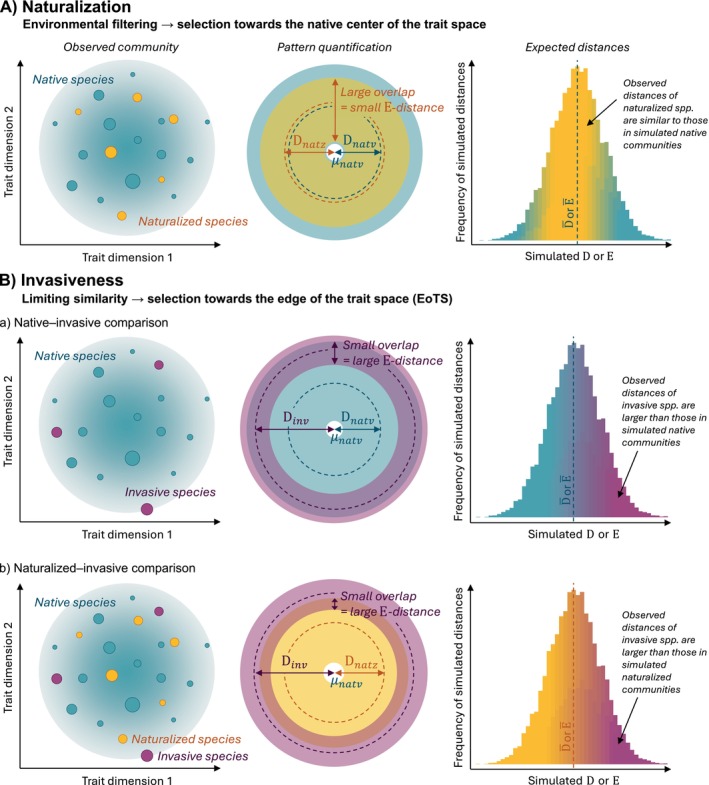
The concept of optimal differentiation to the edge of trait space (EoTS) and its quantification in a local plant community. D quantifies the mean distance of native (natv), naturalized (natz) and invasive (inv) species (community fractions) from the native center ($\mu_{\text{natv}}$) of the multidimensional trait space (for simplicity, only two dimensions are shown). E‐distance (E) quantifies the overlap of the two community fractions. The larger E, the smaller the overlap. (A) Naturalized species integrate near the center of the native community and overlap with native species ($D_{\text{natz}}$ and E are small) because local environmental filters select species with traits similar to the native community. Observed distances of naturalized species should be smaller or similar to those expected for communities of the same size and only with the traits of native species. (B) Invasive species integrate at the periphery of the functional trait space and overlap less with native species ($D_{\mathrm{inv}}$ and E are large) to reduce interspecific competition while still passing environmental filters. When accounting for random expectation, the observed distances of invasive species should be larger than most simulated distances of native species (a). The same should apply to the differences between naturalized and invasive species (b).

There is also growing evidence that novel trait combinations, defined by the position of a species in the functional trait space, are important for alien species to successfully naturalize or become invasive (Divíšek et al. 2018; Hui et al. 2023; Mathakutha et al. 2019; Ordonez et al. 2010). Trait space reflects various trade‐offs related to plant function that ultimately affect species survival, growth and reproduction (Reich 2014). For example, the Leaf Economics Spectrum reflects the differences between species with conservative and acquisitive use of resources (Wright et al. 2004). The Plant Size Spectrum refers to the trait variation related to the size of plants and their organs, which affects the ability of species to compete with other plants (Díaz et al. 2016). Phenological traits may determine reproductive success over a season and consequently the potential of a species to colonize new areas (Fenner and Thompson 2005). Finally, genomic traits drive the expression of other characteristics of a species which influence its adaptation to environmental conditions (Knight et al. 2005). The success of alien species in local plant communities can therefore be investigated within the increasingly used framework of functional distinctiveness (Kondratyeva et al. 2019; Munoz et al. 2023; Violle et al. 2017). This framework quantifies the extent to which a species occupies a unique position in the functional trait space and differs from others in terms of ecological functions such as resource use, growth and reproduction. Functionally distinct alien species may have traits that allow them to exploit resources in ways that native species cannot, thereby increasing their ability to dominate and spread. Building on this idea, the concept of optimal differentiation to the edge of trait space (EoTS; Molofsky et al. 2022) suggests that invasive species (the most successful aliens) insert themselves into the edges of the multidimensional trait space of the native community, where they still pass through environmental filters but avoid competition with native species (Figure 1). The EoTS concept thus postulates that environmental filtering is important for successful naturalization, but becoming invasive requires a certain level of functional distinctiveness to reduce competition with resident species. Although this pattern has been found for species pools of Central European habitats (Divíšek et al. 2018), it has never been studied in local plant communities, that is, small areas where individual species compete directly with each other (Tilman 1982).

The positions of native, naturalized and invasive species in the functional trait space of a local community may also be influenced by habitat type, as adaptations required for the successful establishment and persistence of alien species in a native community can differ among habitats (Fridley et al. 2021). Comparisons between multiple habitats are important for identifying habitat‐specific factors that affect plant invasions. For example, in highly disturbed habitats, trait differences between native and alien species are less pronounced because disturbance acts as a strong environmental filter (Kalusová et al. 2017). The degree to which invasive species alter the phylogenetic and functional structure of resident native species and their abundance may also differ significantly across different habitat types (Lososová, de Bello, et al. 2015; Sodhi et al. 2019).

Here, we use eight morphological, phenological and genomic (hereafter functional) traits to examine the differences between native and alien plant species in 12,460 local communities in six major habitats of Central Europe (Table 1). We quantified the distribution and overlap of native, non‐invasive naturalized (hereafter naturalized) and invasive species in the functional trait space of each community. For each habitat type, we tested whether alien species are close to the native center of the trait space (clustering) or at the edge (dispersion). We hypothesize that the clustering of alien species around the native center and the overlap with native species indicates that environmental filtering plays a crucial role in their integration into local communities (Figure 1A). In contrast, if alien species are distributed at the edges and do not overlap with native species, they likely fill empty niches in the community to avoid niche overlap and competition with native species (Figure 1B). While the first mechanism may be important for species naturalization in a community, the latter should be advantageous for becoming invasive.

**TABLE 1:** Alien plant invasions in local communities (vegetation plots) of six habitat types.

	Grassland vegetation	Ruderal and weed vegetation	Rock and scree vegetation	Wetland vegetation	Scrub vegetation	Forest vegetation
Species pool size	1308	1061	404	745	907	1097
Native spp.	1124	806	326	617	774	957
Naturalized spp.	156	218	67	100	109	102
Invasive spp.	28	37	11	28	24	38
Total number of plots	6554	6265	325	6371	553	4850
No. of invaded plots	3274	5639	183	1702	339	1382
With naturalized spp.	68%	92%	90%	63%	71%	59%
With invasive spp.	65%	74%	28%	57%	69%	74%
With both	34%	65%	17%	20%	11%	32%
Mean species occurrence frequency in invaded plots (± SD)	85 ± 203	108 ± 274	5 ± 8	31 ± 63	10 ± 16	41 ± 85
Native spp.	94 ± 211	85 ± 259	5 ± 7	33 ± 62	10 ± 17	44 ± 86
Naturalized spp.	26 ± 53	169 ± 294	5 ± 10	16 ± 45	6 ± 9	13 ± 36
Invasive spp.	90 ± 337	209 ± 376	5 ± 6	46 ± 105	15 ± 23	36 ± 126
Mean no. of species per invaded plot (± SD)	30.4 ± 12.3	19.5 ± 10.0	8.4 ± 6.5	11.5 ± 7.4	23.0 ± 11.4	28.5 ± 12.6
Native spp.	28.4 ± 12.3	11.6 ± 6.9	6.3 ± 6.1	9.8 ± 6.7	20.1 ± 11.4	26.5 ± 12.9
Naturalized spp.	1.2 ± 1.5	6.6 ± 5.9	1.9 ± 1.5	0.9 ± 1.3	1.8 ± 2.3	0.9 ± 1.2
Invasive spp.	0.8 ± 0.7	1.4 ± 1.3	0.3 ± 0.5	0.8 ± 0.9	1.1 ± 1.0	1.0 ± 0.9
Mean species cover (%) per invaded plot (± SD)	5.8 ± 8.5	7.9 ± 12.1	5.0 ± 7.3	11.3 ± 18.0	7.6 ± 9.9	9.6 ± 14.5
Native spp.	5.5 ± 3.0	6.6 ± 6.0	3.2 ± 2.2	13.2 ± 12.6	8.9 ± 6.9	7.5 ± 4.3
Naturalized spp.	3.3 ± 6.0	6.3 ± 9.4	7.5 ± 10.0	15.1 ± 27.1	3.3 ± 4.2	4.1 ± 7.6
Invasive spp.	8.8 ± 13.9	11.8 ± 18.6	3.6 ± 6.7	3.9 ± 9.2	10.0 ± 15.0	16.9 ± 22.5

*Note:* Species pool size is the total number of species recorded in the vegetation plots of each habitat type. Invaded plots are those in which at least one native species occurred together with at least one naturalized or invasive species or with both. The species occurrence frequency is expressed as the number of plots of the habitat type in which the species occurs.

## 2 Materials and Methods

### 2.1 Plant‐Community Data

The data on species composition of local plant communities comes from 24,918 vegetation plots sampled in the Czech Republic, stored in the Czech National Phytosociological Database (Chytrý and Rafajová 2003; GIVD code EU‐CZ‐001). The size of the vegetation plots in the dataset was restricted to 1–100 m^2^ for non‐forest plots and 100–625 m^2^ for forest plots. Each plot was classified to one of the six main terrestrial habitat types of Central Europe (Table 1) using a computer‐based expert system (Chytrý et al. 2020): (1) grassland and heathland vegetation below the timberline (hereafter grassland vegetation; 6554 plots), (2) ruderal and weed vegetation (6265 plots), (3) rock and scree vegetation (325 plots), (4) wetland vegetation (6371 plots), (5) scrub vegetation (553 plots) and (6) forest vegetation (4850 plots). Juvenile trees occurring in the plots of habitats 1–4 were removed as their functional trait values describe fully grown individuals. All non‐seed plants were also removed. Based on the classified vegetation plots, a list of species occurring in each habitat type (species pool) was created and the frequency of occurrence of each species was recorded. The size of the habitat species pools, species occurrence frequencies and percentage covers in vegetation plots of each habitat type are shown in Table 1.

### 2.2 Alien Species Classification

Our dataset contained 1705 seed plant species, of which 309 were aliens in the Czech Republic. These alien species represent a quite heterogeneous group in terms of their traits, origin, time of introduction, and invasion status (Pyšek et al. 2012). It has been shown that historically older and more stable forests are phylogenetically more diverse and less invaded than younger and less stable (frequently disturbed) habitats such as grasslands and other open habitats (Lososová, de Bello, et al. 2015; Lososová, Šmarda, et al. 2015). These habitats are invaded by species from the same lineages as native species and are preadapted to similar types of disturbances (Lososová, de Bello, et al. 2015). Typically, they are weeds from families Lamiaceae and Poaceae. The most successful invaders in the country are from families Asteraceae, Poaceae, Brassicaceae, Rosaceae, Fabaceae or Amaranthaceae. The information on species invasion status was taken from Pyšek et al. (2012), where it was based on criteria introduced by Richardson et al. (2000). Naturalized species (259 in our dataset) were defined as alien species that form self‐sustaining populations over several life cycles without human‐facilitated input of propagules. Invasive species (50), a subset of naturalized species, were defined as alien species that produce offspring in very large numbers and spread over long distances (Richardson et al. 2000). The most frequent naturalized and invasive species in each habitat type are listed in Figures S1 and S2. Of the 24,918 vegetation plots in our dataset, native species co‐occurred with alien species in 12,460 plots. These plots were used in subsequent analyses.

### 2.3 Functional Traits

Data on species' functional traits were compiled from the databases Pladias (Chytrý et al. 2021), LEDA (Kleyer et al. 2008), GIFT (Weigelt et al. 2020) and various literature sources (Table S1). For each species, the following functional traits were used: (1) maximum plant height (m), (2) seed mass (mg), (3) leaf area (mm^2^), (4) specific leaf area (SLA; mm^2^ mg^−1^), (5) leaf dry matter content (LDMC; mg g^−1^), (6) middle of the flowering period (month), (7) length of the flowering period (number of months) and (8) 2C genome size (Mbp). The strongest Spearman correlation was between maximum height and leaf area (*ρ* = 0.553; Figure S3). Only the maximum plant height, middle and length of the flowering period were available for all species in our dataset. For the remaining traits, data were only available for 68% (LDMC), 73% (leaf area), 78% (SLA) and 85% (seed mass and 2C genome size) of the species. Since the common practice of removing missing data not only reduces the sample size but may also introduce bias, we imputed missing trait values using three methods described in Table S2. Here, we present only results based on missForest imputation (Stekhoven and Bühlmann 2012), while results from MICE‐PMM (van Buuren and Groothuis‐Oudshoorn 2011) and Phylopars (Goolsby et al. 2024) are in Data S1. Before analyses, all traits except the middle of the flowering period were log10‐transformed. All traits were then scaled to zero mean and unit variance. Finally, all species were assigned to five broadly defined life forms: (1) macrophanerophytes (woody plants over 10 m tall, mainly trees; 60 species), (2) nanophanerophytes (woody plants up to 10 m tall, i.e., mainly shrubs; 66 species), (3) chamaephytes (dwarf shrubs and semi‐shrubs; 55 species), (4) herbs (1522 species) and (5) epiphytes (2 species). For the overall trait spectrum of each habitat visualized using principal component analysis, see Figures S4–S9.

### 2.4 Species Distribution in the Functional Trait Space

For each vegetation plot, we constructed an eight‐dimensional trait space where each axis was represented by one of the eight functional traits. Each species present in the community was then represented as a unique point in this trait space. To quantify the distribution of community fractions (native, naturalized and invasive species) in this trait space, we measured (1) the mean distance of each fraction from the native center, and (2) the overlap of each two fractions. Mean distance from the center (Figure S10a) was calculated as:

$$D_{X}=\frac{1}{n_{X}}\sum_{i\in X}\left\|x_{i}-\mu_{\text{natv}}\right\|,$$

where $\left\|\cdot \right\|$ denotes Euclidean norm, $x_{i}$ is a vector of trait values of i‐th species from group X (i.e., native, naturalized or invasive), $n_{X}$ is the size of this group and $\mu_{\text{natv}}$ is the trait space center (mean vector) for native species. $D_{X}$ is thus analogous to the unweighted functional dispersion index by Laliberté and Legendre (2010).

The overlap was quantified using E‐distance, which measures the dissimilarity between two probability distributions (Rizzo and Székely 2010, 2016). We chose this metric because it detects differences in both the location (average position) and dispersion (whether one group is functionally more or less diverse than the other group) of the compared groups. The empirical E‐distance for two groups of species (Figure S11a) is calculated as:

$$E_{\mathrm{XY}}=\frac{2}{n_{X}n_{Y}}\sum_{i\in X}\sum_{j\in Y}\left\|x_{i}-y_{j}\right\|-\frac{1}{n_{X}^{2}}\sum_{i\in X}\sum_{j\in X}\left\|x_{i}-x_{j}\right\|-\frac{1}{n_{Y}^{2}}\sum_{i\in Y}\sum_{j\in Y}\left\|y_{i}-y_{j}\right\|,$$

where $\left\|\cdot \right\|$ denotes Euclidean norm, $x_{i}$ and $y_{j}$ are vectors of trait values of i‐th and j‐th species from groups X and Y, respectively, and n is the size of respective group. In the second and third term, if i=j, then $\left\|x_{i}-x_{j}\right\|=0$ and $\left\|y_{i}-y_{j}\right\|=0$, respectively. If the distribution of the two species groups in the trait space is identical (complete overlap), the E‐distance is zero. The higher the value, the more the two distributions differ. The magnitude of E‐distance, therefore, directly reflects the degree of functional segregation (or dissimilarity) between native and alien species (Figure S12).

In addition, we developed weighted versions of the abovementioned metrics, in which square‐root‐transformed species percentage covers in the vegetation plots were used as weights. The calculation of these weighted versions is explained in Figures S10b and S11b. The results of associated analyses can be found in Data S1.

As an alternative to the Euclidean distance used by both the abovementioned metrics (D and E), we also used the Gower (1971) distance with the extension by Podani (1999) to ordinal variables, considering the middle of the flowering period as this type of variable. For each vegetation plot, we then calculated D and E metrics based on principal coordinate analysis (PCoA) axes from a Gower species dissimilarity matrix instead of the original traits (Laliberté and Legendre 2010). The results based on this approach are included in Data S1.

For scrub and forest vegetation plots, the abovementioned metrics were calculated separately for herb‐layer plants (herbs and dwarf shrubs) and phanerophytes (nanophanerophytes and macrophanerophytes).

### 2.5 Null Models

We used null models to determine whether native and alien community fractions in each plot are more clustered or dispersed around the center of the native trait space than would be expected for randomly assembled communities and to account for different sizes of individual community fractions. For each vegetation plot in which naturalized or invasive species were compared with native species, we simulated trait composition by a stratified random drawing of species from the habitat's native species pool. For the comparison between naturalized and invasive species, trait composition of these community fractions was simulated by drawing from the habitat's naturalized species pool. The number of species in the plot, the proportion of different plant life forms and the cover of individual species (when the weighted versions of D and E metrics were used) were maintained in each simulation. Selections from species pools were either (1) unweighted (i.e., completely random), (2) weighted by species occurrence frequency in the habitat, with more frequent species having a higher chance to be selected or (3) weighted by the probability of species occurrence in the plot, so that species likely to occur in the plot based on co‐occurrence with resident native species had a higher chance of being selected. Occurrence probability was calculated using the Beals index (Beals 1984) implemented in the R package vegan (Oksanen et al. 2022). We present here only the results based on the Beals weighting, while the results of null models 1 and 2 are in Data S1.

We repeated the simulations 999 times for each vegetation plot to obtain the distribution of the simulated D values for each community fraction. We then calculated the deviation from the null expectation (ΔD) as the observed D minus the mean of the simulated values. Positive ΔD indicates dispersion towards the edge of the trait space, while negative ΔD indicates clustering around the center, relative to the null expectation for a community fraction of the same size. We intentionally did not calculate the standardized effect size (SES) because it is sensitive to variation in species richness and therefore less suitable for comparisons between groups with different numbers of species (Sandel 2018).

For E‐distance measuring the overlap of each two community fractions present in the plot, we used the abovementioned simulations to calculate the probability (also called empirical *p*‐value) that the observed overlap is larger (E‐distance is smaller) than in simulated communities. Probability was calculated as (the number of simulated E‐distance values that are equal to or greater than the observed one + 1)/(the number of simulations + 1). Probability values approaching zero indicate that the observed overlap is smaller (E‐distance is larger) than in simulated communities, while the value of one indicates larger observed overlap (smaller E‐distance) than in all simulated communities.

For each habitat type, the deviations from expected distance (ΔD) and the overlap probabilities were summarized using boxplots and density plots. To test whether ΔD values for individual community fractions are significantly different within the habitat type, we used the Wilcoxon signed‐rank test for paired samples. The resulting *p*‐values were adjusted using Benjamini and Hochberg's (1995) method. The effect size (*r*) for the difference between compared community fractions was calculated by dividing the *z*‐statistic from the Wilcoxon test by the square root of the sample size (*N*). The effect size (in absolute value) is considered to be small when *r* < 0.3, medium when 0.3 ≤ *r* < 0.5 and large when *r* ≥ 0.5. These analyses were performed not only within the eight‐dimensional trait space of each plot, but also for each functional trait separately.

### 2.6 Traits Driving Functional Differences

We used regression trees (CART; Breiman et al. 1984), to analyze which individual traits contribute most to the dispersion (∆D>0) or clustering (∆D<0) of naturalized and invasive community fractions in the trait space of each vegetation plot. The ΔD values for plots of different habitat types were pooled and used as the dependent variable while the mean values of individual traits for each community fraction were used as explanatory variables. Optimal tree size was determined by 10‐fold cross‐validation as the smallest tree that reached the minimum cross‐validation error plus 1 SE. Besides the primary splitter variable, we also identified surrogates for each node, that is, variables allocating at least 90% of the cases to the same cluster as the primary splitter.

All analyses were performed in R software version 4.2.1 (R Core Team 2022), and the packages used are listed in Table S3.

## 3 Results

**FIGURE 2:**
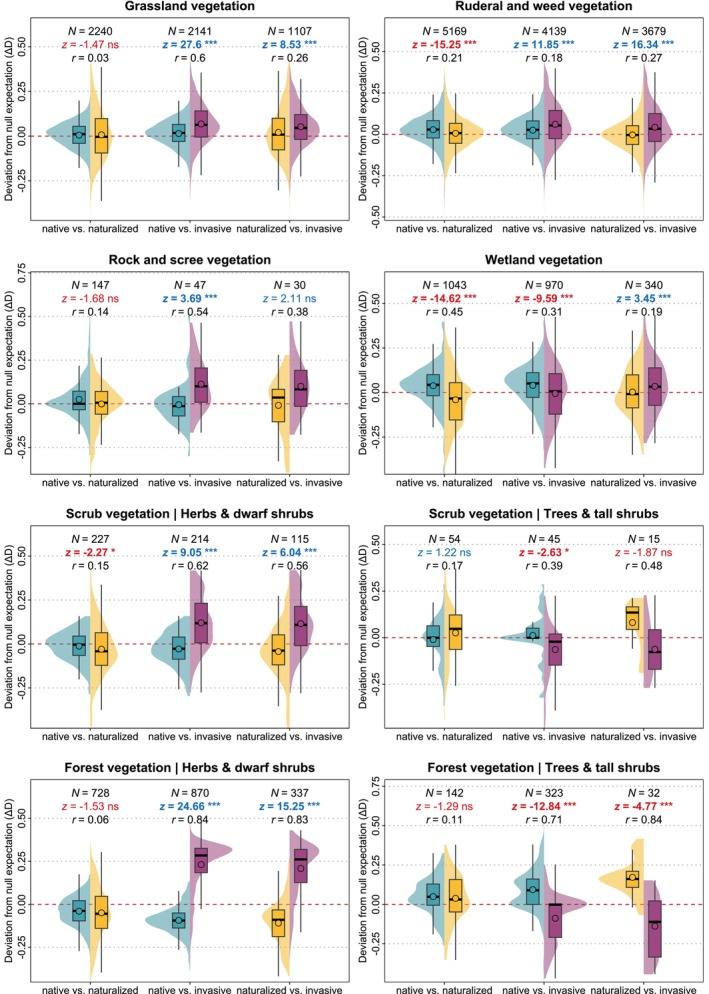
Distances of native (cyan), naturalized (gold) and invasive (magenta) community fractions from the native center of the eight‐dimensional trait space in each vegetation plot. Distances are expressed as the deviation (ΔD) from the mean expected distance for community fractions of the same size simulated by the weighted random drawing of native species (or naturalized species for the ‘naturalized vs. invasive’ comparison) from the habitat species pools. Positive values indicate a tendency towards overdispersion (greater distance from the center of the native trait space than expected for the simulated community), while negative values indicate a tendency towards clustering (shorter distance from the center than expected). *N* is the number of plots, *z* is the statistical value of the Wilcoxon signed‐rank test for paired samples. *p*‐values were adjusted following Benjamini and Hochberg (1995): ***p < 0.001, ** 0.001 ≤ *p* < 0.01, * 0.01 ≤ *p* < 0.05, ns *p* ≥ 0.05. Statistically significant results (*p* < 0.05) are in bold. Red and blue fonts indicate negative and positive differences, respectively. The effect size *r* (in absolute value) associated with the Wilcoxon test is considered small when *r* < 0.3, medium when 0.3 ≤ *r* < 0.5, and large when *r* ≥ 0.5. The thick horizontal line in each box indicates the median, the dot indicates the mean, the bottom and top of each box indicate the 25th and 75th percentiles, respectively, and whiskers represent either the maximum/minimum value or 1.5 × interquartile range, whichever is closer to the mean.

We found distinct patterns in the distances of each community fraction from the native center of the eight‐dimensional functional trait space in each local community (for distances within each individual trait, see Figures S13–S14). The distances of native ($∆D_{\text{natv}}$) and naturalized ($∆D_{\text{natz}}$) species in most habitats were similar or even smaller (indicating clustering) than expected for a community fraction of the same size composed of only traits of native species pool. In contrast, the distances of invasive species ($∆D_{\mathrm{inv}}$) were larger in five of six habitat types (except wetlands; Figure 2). However, in scrub and forest vegetation, they were only larger in the herb layer. The magnitude of the within‐habitat differences between co‐occurring community fractions depended on the habitat type. We found very small, mostly negative, differences between native and naturalized community fractions. Their effect size ranged from *r* = 0.03 in grasslands (*z* = −1.47, *p* = 0.143) to *r* = 0.45 in wetlands (*z* = −14.6, *p* < 0.001). This indicates that the distribution of naturalized species in the community trait space is mostly not different from native species and is more clustered than expected. In contrast, invasive community fractions were usually distributed further away than co‐occurring native species. The magnitude of the within‐habitat differences ranged from *r* = 0.18 for ruderal and weed vegetation (*z* = 11.8, *p* < 0.001) to *r* = 0.84 for forest herb layers (*z* = 24.7, *p* < 0.001). In scrub and forest vegetation, however, this pattern can largely be attributed to *Impatiens parviflora* (Figure S15). On the other hand, invasive trees and tall shrubs showed an even stronger clustering than co‐occurring native species (*r* = 0.39, *z* = −2.63, *p* = 0.03 for scrub vegetation and *r* = 0.71, z = −12.84, *p* < 0.001 for forest vegetation). A similar pattern was also observed in wetland vegetation (*r* = 0.31, *z* = −9.59, *p* < 0.001).

When comparing the distances of naturalized ($∆D_{\text{natz}}$) and invasive ($∆D_{\mathrm{inv}}$) community fractions (expressed as deviations from the expected distance of a naturalized community fraction of the same size), we found a pattern similar to the ‘native vs. invasive’ comparison. In all habitats, invasive species were distributed closer to the edge of the community trait space than were co‐occurring naturalized species. The magnitude of the differences ranged from *r* = 0.19 for wetlands (*z* = 3.45, *p* < 0.001) to *r* = 0.83 for forest understories (*z* = 15.2, *p* < 0.001). The exceptions included tree and shrub layers in scrub and forest vegetation where invasive community fractions were clustered around the trait space center more than co‐occurring naturalized species (*r* = 0.48 and 0.84, *z* = −1.87 and –4.77, *p* = 0.096 and 0.001, respectively).

After weighting the distances of individual community fractions (D) by species covers, the observed effect sizes decreased slightly for most comparisons (Figure S18). However, weighting changed the direction of difference and statistical significance in only a few cases, including the ‘native vs. naturalized’ comparison for grasslands, scrub and forest vegetation. In these cases, the observed differences were very small and on the edge of statistical significance.

**FIGURE 3:**
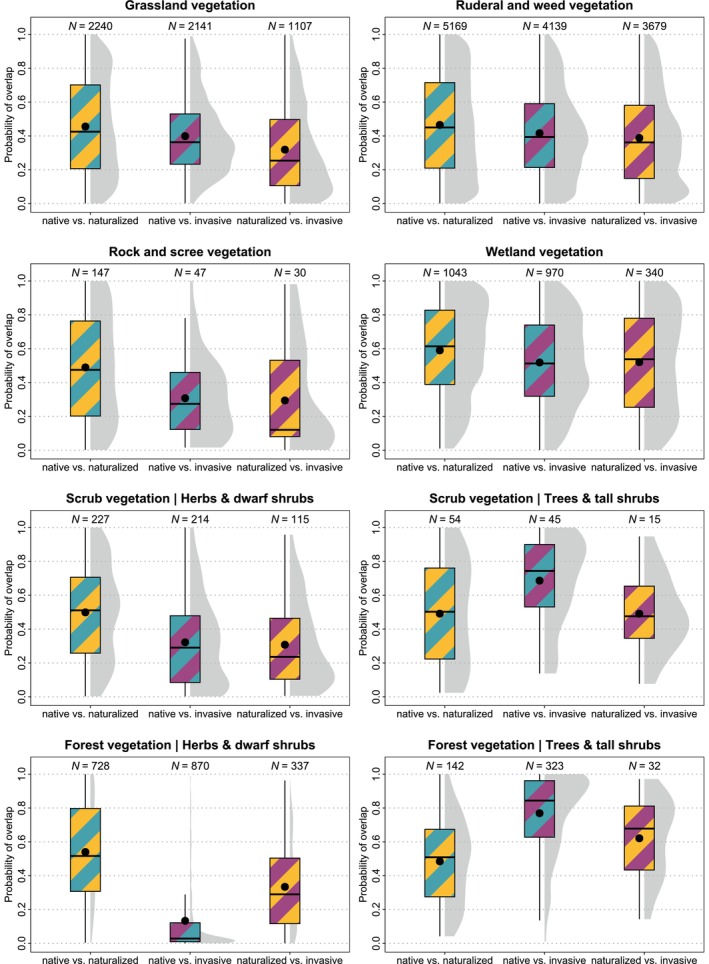
Probability of overlap between native and naturalized species (cyan/gold), native and invasive species (cyan/magenta) and naturalized and invasive species (gold/magenta) in the eight‐dimensional trait space of each plot. Probability was calculated by comparing the observed overlap value, expressed by E‐distance, with the distribution of 999 simulated overlap values for two community fractions with corresponding sizes and traits of only native (for ‘native vs. naturalized’ and ‘native vs. invasive’ comparisons) or naturalized species (for ‘naturalized vs. invasive’ comparison). Probability values approaching zero indicate that the observed overlap is smaller (E‐distance is larger) than in simulated communities, while values approaching one indicate larger observed overlap (smaller E‐distance) than in all simulated communities. *N* is the number of plots in which native and alien species were compared.

Using E‐distance, we found that in all habitat types (except the tree layer in scrub and forests), native and naturalized species overlapped more than native and invasive species and more than naturalized and invasive species. Null models showed that the observed overlap of native and naturalized community fractions did not differ from that in most simulated communities with native traits only (Figure 3). In other words, the median of observed overlap values ranged from the 40th percentile of simulated values for wetlands to the 58th percentile for grasslands. Native and invasive community fractions overlapped less. In most habitats except for wetlands and tree and shrub layers in scrub and forest vegetation, the median observed overlap was smaller (E‐distance was larger) than in 60% (ruderal and weed vegetation) of simulated communities, and in three habitats it was even smaller than in 70% of simulated communities. The overlap of naturalized and invasive community fractions in these habitats was also small. These community fractions overlapped less than comparable fractions in 64% (ruderal and weed vegetation) of simulated communities with only traits of naturalized species. For probabilities of overlap after removing *Impatiens parviflora* , see Figure S16.

The use of species covers as weights in the calculation of E‐distances led to very similar results. The probability of overlap increased slightly in a few cases, including the ‘native vs. naturalized’ comparison in ruderal and weed vegetation and the ‘native vs. invasive’ comparison in grasslands (Figure S19).

All alternative approaches to these analyses using (1) the PCoA axes from the Gower distance matrix (Figures S20–S23), (2) the data from the MICE‐PMM and Phylopars imputations (Figures S24–S31) and (3) alternative null models (Figures S32–S39) produced very similar results. Changes only occurred in comparisons where the differences between community fractions were small or the sample size was small, while the overall pattern remained unchanged.

**FIGURE 4:**
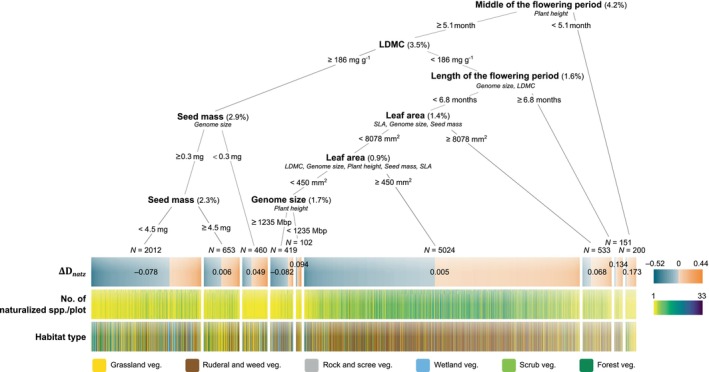
Decision tree of functional traits contributing to the clustering ($\Delta D_{natz}<0$; blue‐grey colour scale) or dispersion ($\Delta D_{natz}>0$; orange colour scale) of naturalized community fractions when the distance from the trait space center expected for a native community fraction of the same size is accounted for. Only herbs and dwarf shrubs were analyzed. The primary splitter variable at each node is shown in bold, and its split value is indicated below the node. The numbers in parentheses indicate the percentage variance explained by each splitting. Surrogates (i.e., variables that assign at least 90% of the cases to the same group as the primary splitter) are indicated in smaller italic letters below the primary splitter. The mean $∆D_{\text{natz}}$ and the number of community fractions (*N*) included in each terminal node are shown within and above the uppermost colour scale, respectively. The total variance explained by the tree is 18.5%.

**FIGURE 5:**
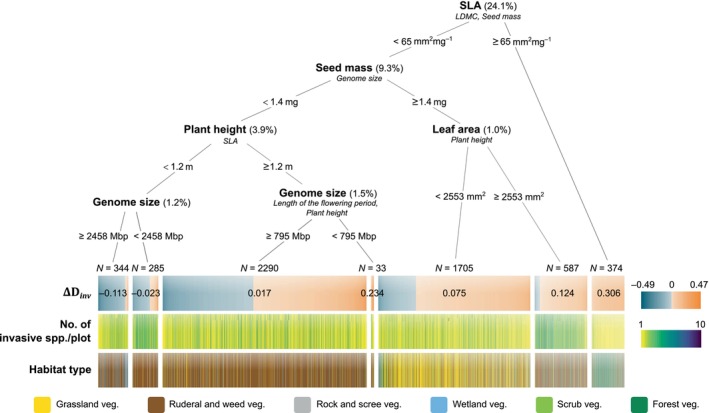
Decision tree of functional traits contributing to the clustering ($\Delta D_{inv}<0$; blue‐grey colour scale) or dispersion ($\Delta D_{inv}>0$; orange colour scale) of invasive community fractions when the distance from the trait space center expected for a naturalized community fraction of the same size is accounted for. For details, see Figure 4. The total variance explained by the tree is 41%.

Finally, regression trees revealed which individual functional traits contributed most to the clustering (∆D<0) or dispersion (∆D>0) of naturalized and invasive community fractions in the eight‐dimensional trait space of each plot across all habitat types. We found that the most important traits influencing the distance of naturalized herbs and dwarf shrubs from the native center of the trait space were the time of flowering, LDMC, length of flowering and seed mass (Figure 4). On the other hand, SLA, seed mass, plant height and leaf area, followed by genome size, were the most important traits for invasive species (Figure 5). However, the effect of SLA was largely attributable to *Impatiens parviflora* (Figure S16).

## 4 Discussion

Using eight functional traits to explore the integration of alien plant species into local communities of six main habitat types in Central Europe, we showed that each of the two opposing hypotheses, that is, environmental filtering (Keddy 1992) and limiting similarity (MacArthur and Levins 1967), is important at a different stage of the invasion process (Richardson et al. 2000). The distribution of naturalized species in the trait space of most communities appeared to be very similar to native species, suggesting that they have similar adaptations to local environmental conditions and resource use as native species. This preadaptation may facilitate their naturalization in the community, and environmental filtering appears to be the most important factor at this stage of the introduction–naturalization–invasion continuum. However, this convergence in traits may also result from competitive hierarchy (Mayfield and Levine 2010). If certain trait values confer superior fitness in a given environment (e.g., greater height for light competition), both successful native and alien species may converge on this strategy while suppressing other (e.g., shorter) species. This may lead to a community where all remaining species are functionally similar. Therefore, the observed similarity of naturalized to native species may reflect not only adaptations to the abiotic environment but also adaptations to competition from the native species. Regardless of the underlying mechanism, the similarity of naturalized to native species may also lead to significant niche overlap. Functionally similar species are often assumed to utilize resources in a similar way, particularly for the resource‐acquisition traits (e.g., Wright et al. 2004), which can increase the intensity of competition. Increased competition may potentially act as a barrier to the progression of naturalized species to the invasive stage.

In contrast, invasive species in communities of most habitat types exhibited trait combinations that differed from native and naturalized species, which shifted them to the edge of the functional trait space of the community. This suggests that it may be advantageous for an already naturalized alien species to differ, at least to some degree, from native species to become invasive. Alternatively, this functional distinctiveness could be the result of competitive exclusion, where invasive species have displaced native species with the most similar strategies. However, inferring competition from trait patterns is challenging, as the link between trait differences and the strength of competitive interactions is often complex and context‐dependent (Levine et al. 2025). Although the occurrence at the edge of the trait space is not direct proof of competition avoidance, it represents a clear strategy of functional differentiation. This may help invasive species to exploit new niches, use resources more effectively and invest them to gain dominance over co‐occurring native species. The observed patterns largely persisted after weighting the distances by species covers. The weighting caused a slight decrease in the magnitude of most differences, but significantly affected only a few comparisons where the observed differences were very small. This may indicate that successful alien species use strategies that are at least partially similar to those of dominant native species.

Our results are consistent with other studies that have documented different traits or trait combinations of invasive species (Gallagher et al. 2015; van Kleunen et al. 2010; Mathakutha et al. 2019), but also with the differences between native and alien species observed at the level of habitat species pools (Divíšek et al. 2018), where plant height was the main trait distinguishing invasive species. Here, we also found evidence that tall invasive species are functionally more distinct from both native and naturalized species, but the effect of SLA on the shift of invasive species towards the edge of the community's trait space was even more pronounced, particularly due to *Impatiens parviflora* , which is an exceptionally successful invasive species in Central European forests (Wagner et al. 2017). Being taller and able to outcompete other species by shading is indeed an advantage, but rapid resource acquisition seems to be equally or even more important within local communities. A larger SLA indicates thinner or less dense leaves, which has several implications for a plant's growth strategy, resource use efficiency and adaptability to varying environmental conditions. A large SLA is often associated with a high relative growth rate and is characteristic of species adapted to resource‐rich environments, where the ability to rapidly utilize available light, water and nutrients is crucial (Kraft et al. 2015; Wright et al. 2004). This finding is consistent with other studies that have also reported larger SLA for invasive species (Gallagher et al. 2015; Hamilton et al. 2005; Leishman et al. 2007; Ordonez et al. 2010).

Besides SLA and plant height, we identified a large effect of seed mass. Unlike other studies showing that small seed mass correlates with species invasiveness (Hamilton et al. 2005; Leishman et al. 2007; Ordonez et al. 2010), we found that invasive species tend to have larger seeds than native and naturalized species. Heavier seeds often contain more reserves, which support seedling growth and increase their tolerance to stress factors such as shading from co‐occurring plants. Thus, heavier seeds can give rise to more vigorous seedlings that can outcompete neighbouring plants (Moles and Westoby 2006; Turnbull et al. 1999) and become dominant in a community.

We also found some evidence for the effects of genome size. According to the Large Genome Constraint Hypothesis (Knight et al. 2005), plants with larger genomes may be less tolerant to environmental stress and less plastic under stress gradients. A small genome may be associated with earlier germination, faster plant growth, higher photosynthetic rate and larger SLA (Bennett 1987; Knight and Ackerly 2002). Pyšek et al. (2023) found that a small genome is advantageous during naturalization, but not for invasive spread. However, here we found that both small and large genomes can shift species to the edge of the trait space. The effect of genome size, therefore, remains unclear and requires further investigation.

Previous studies have found that successful invaders have early or long flowering periods (Cadotte and Lovett‐Doust 2001; Lake and Leishman 2004; Pyšek et al. 2003). A longer flowering may provide more opportunities for pollination, potentially leading to higher reproductive success over a season and the colonization of new areas (Fenner and Thompson 2005). We found that in some communities, naturalized species flowered earlier and for a longer time than native species, but such communities were few. In most communities, the flowering phenology of both species groups was similar. For invasive species, flowering phenology did not appear to differ from native or naturalized species. The effect of flowering phenology on naturalization or invasiveness of alien species thus probably depends on the ecological context, not only in the invaded range but also in the native range, as it is a conservative trait (Godoy, Castro‐Díez, et al. 2009; Godoy, Richardson, et al. 2009).

Our dataset comes from the temperate zone of Central Europe and habitats with a large proportion of herbaceous plants. Some habitats (e.g., ruderal and weed vegetation) are highly invaded, while others (e.g., wetlands and forests) are invaded much less. The most widespread naturalized species, such as *Tripleurospermum inodorum* or *Convolvulus arvensis* , and the most successful invaders, such as *Arrhenatherum elatius, Cirsium arvense* or *Impatiens parviflora* , were recorded at most sites of their suitable habitats. Such species largely drive the observed patterns. When we removed *I. parviflora* , the number of plots in which invasive species occurred at the edge of the trait space decreased considerably, especially in forest understories. Exceptions to the pattern observed in most habitats include the tree and shrub layers in forests and scrub and partly also wetlands. This suggests that the success of the most common invaders in these habitats ( *Robinia pseudoacacia* in forests and *Bidens frondosa* in wetlands) may depend on traits other than those included in our study (e.g., nitrogen‐fixing capacity, dispersal mode or tolerance to waterlogging).

We used trait data from the literature and databases, which may introduce some error compared to direct measurements. However, measuring traits in our nationwide dataset was not feasible. Therefore, our results cannot reflect intraspecific trait variability among communities. Another disadvantage is the incompleteness of trait values. In our study, three of eight traits had complete data, but the other five had, on average, 22% of missing values. To account for the variability introduced by different trait imputation methods, we analyzed datasets from three commonly used algorithms (missForest, MICE‐PMM and Phylopars). This confirmed that the observed patterns are robust and largely insensitive to the chosen method.

In conclusion, our study provides evidence that the functional integration of alien species depends on the stage of invasion and the habitat type in which it happens. The results suggest that environmental filtering primarily shapes naturalization, selecting for aliens that are functionally similar to native species, whereas functional differentiation becomes crucial for the subsequent stage, that is, invasion. While we recognize the challenges of inferring ecological processes from trait patterns, our study highlights the value of this approach for revealing the differences between co‐occurring native and alien species and understanding how alien species integrate into local plant communities. Future studies could test how trait differences affect direct competition between native, naturalized and invasive species, or include intraspecific trait variation and additional key traits, such as those related to dispersal or below‐ground resource acquisition. This would provide a more complete picture of the assembly processes in invaded communities.

## Author Contributions

J.D., P.P., D.M.R., N.J.G., B.B., J.M. and M.C. conceived the ideas. J.D., Z.L. and M.C. prepared the data. J.D., N.J.G. and B.B. designed the methodology. J.D. analyzed the data. J.D. led the writing of the manuscript. P.P., D.M.R. and M.C. contributed to the writing. All authors commented on the manuscript.

## Conflicts of Interest

The authors declare no conflicts of interest.

## Supporting information


**Data S1:** ele70235‐sup‐0001‐DataS1.docx.

## Data Availability

All data and R codes used in the analyses are publicly available in the Zenodo repository: https://doi.org/10.5281/zenodo.17116216.
