# Supplementary material for: Naturalized and Invasive Species Integrate Differently in the Trait Space of Local Plant Communities
Source: Ecol Lett. 2025 Nov 9;28(11):e70235. doi: 10.1111/ele.70235 (PMC12596936; doi:10.1111/ele.70235)
Supplement: Supplementary file 1 — Data S1: ele70235‐sup‐0001‐DataS1.docx. [file ELE-28-0-s001.docx]

**Supporting Information for**

**Naturalized and invasive species integrate differently in the trait space of local plant communities**

Jan Divíšek^1,2^, Petr Pyšek^3,4^, David M. Richardson^3,5^, Nicholas J. Gotelli^6^, Brian Beckage^7,8,9^, Jane Molofsky^7^, Zdeňka Lososová^1^ & Milan Chytrý^1^

1. Department of Botany and Zoology, Faculty of Science, Masaryk University, Kotlářská 2, 611 37 Brno, Czech Republic.
2. Department of Geography, Faculty of Science, Masaryk University, Kotlářská 2, 611 37 Brno, Czech Republic.
3. Department of Invasion Ecology, Institute of Botany, Czech Academy of Sciences, 252 43 Průhonice, Czech Republic.
4. Department of Ecology, Faculty of Science, Charles University, Viničná 7, 128 43 Praha 2, Czech Republic.
5. Centre for Invasion Biology, Department of Botany and Zoology, Stellenbosch University, Stellenbosch 7602, South Africa.
6. Department of Biology, University of Vermont, Burlington, VT 05405, USA.
7. Department of Plant Biology, University of Vermont, Burlington, VT 05405, USA.
8. Department of Computer Science, University of Vermont, Burlington, VT 05405, USA.
9. Gund Institute for Environment, University of Vermont, Burlington, VT 05405, USA.

Contents

[Appendix S1 – Details on data used 2](#_Toc204092934)

[Appendix S2 – Supplementary methods 12](#_Toc204092935)

[Appendix S3 – Distances (D) within individual traits 17](#_Toc204092936)

[Appendix S4 – Results after excluding *Impatiens parviflora* 20](#_Toc204092937)

[Appendix S5 – Results for weighted distance metrics (wD and wE) 24](#_Toc204092938)

[Appendix S6 – Results based on the PCoA axes from the Gower dissimilarity matrix 27](#_Toc204092939)

[Appendix S7 – Results based on the data from the MICE-PMM and Phylopars imputations 32](#_Toc204092940)

[Appendix S8 – Results of the alternative null models 41](#_Toc204092941)

[References 50](#_Toc204092942)

# Appendix S1 – Details on data used


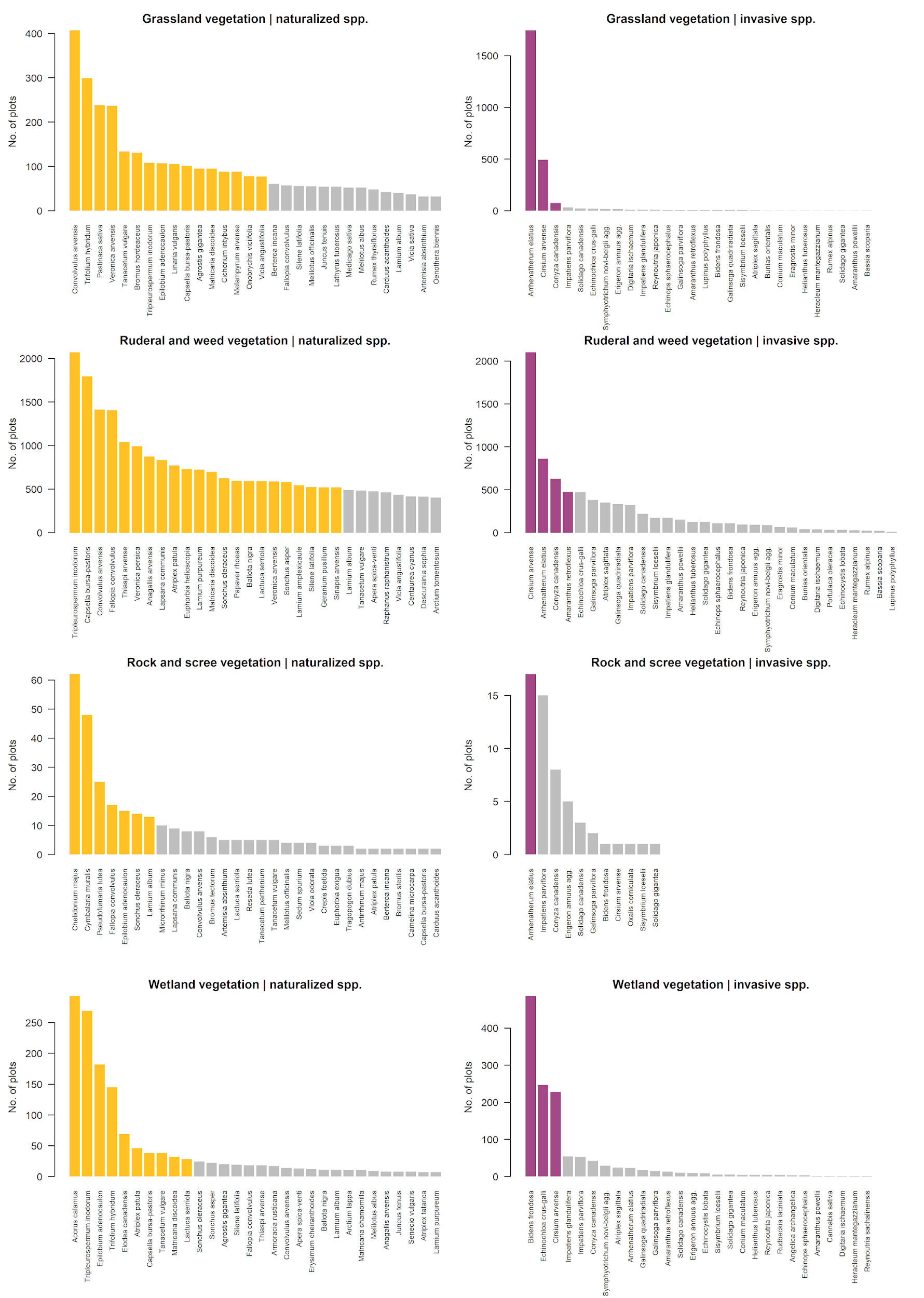


Fig. S1. The most frequent alien species (up to 30 species) in plots of grassland vegetation, ruderal and weed vegetation, rock and scree vegetation and wetland vegetation. Ten percent of the most frequent naturalized and invasive species are shown in gold and magenta, respectively.


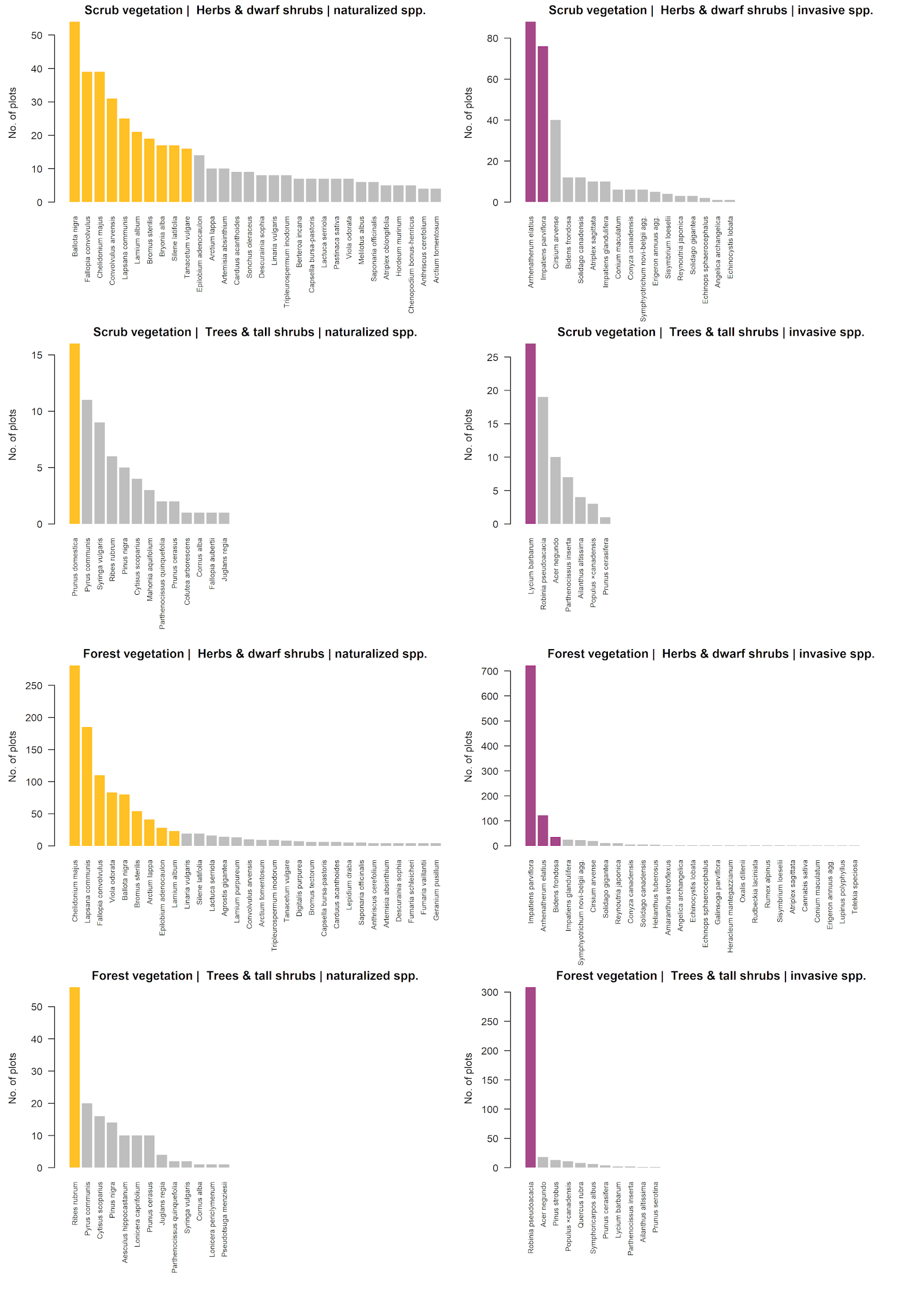


Fig. S2. The most frequent alien species (up to 30 species) in plots of scrub and forest vegetation. Ten percent of the most frequent naturalized and invasive species are shown in gold and magenta, respectively.

**Table S1. Sources of data on species functional traits.**

| **Maximum plant height:** |
| --- |
| Kubát, K., Hrouda, L., Chrtek, J., Kaplan, Z., Kirschner, J. & Štěpánek, J. (2002). *Klíč ke květeně České republiky [Key to the flora of the Czech Republic]*. Academia, Praha. |
| **Seed mass:** |
| Chytrý, M., Danihelka, J., Kaplan, Z., Wild, J., Holubová, D., Novotný, P., *et al.* (2021). Pladias Database of the Czech Flora and Vegetation. *Preslia*, 93, 1–87. |
| Kleyer, M., Bekker, R. m., Knevel, I. c., Bakker, J. p., Thompson, K., Sonnenschein, M., *et al.* (2008). The LEDA Traitbase: a database of life-history traits of the Northwest European flora. *Journal of Ecology*, 96, 1266–1274. |
| Weigelt, P., König, C. & Kreft, H. (2020). GIFT – A Global Inventory of Floras and Traits for macroecology and biogeography. *Journal of Biogeography*, 47, 16–43. |
| **Leaf area:** |
| E-Vojtkó, A., Balogh, N., Deák, B., Kelemen, A., Kis, S., Kiss, R., *et al.* (2020). Leaf trait records of vascular plant species in the Pannonian flora with special focus on endemics and rarities. *Folia Geobot*, 55, 73–79. |
| Findurová, A. (2018). Variabilita listových znaků SLA a LDMC vybraných druhů rostlin České republiky [Variability of leaf traits SLA and LDMC in selected species of the Czech flora]. Master thesis. Masaryk University, Brno. |
| Kleyer, M., Bekker, R. m., Knevel, I. c., Bakker, J. p., Thompson, K., Sonnenschein, M., *et al.* (2008). The LEDA Traitbase: a database of life-history traits of the Northwest European flora. *Journal of Ecology*, 96, 1266–1274. |
| Weigelt, P., König, C. & Kreft, H. (2020). GIFT – A Global Inventory of Floras and Traits for macroecology and biogeography. *Journal of Biogeography*, 47, 16–43. |
| **Specific leaf area:** |
| E-Vojtkó, A., Balogh, N., Deák, B., Kelemen, A., Kis, S., Kiss, R., *et al.* (2020). Leaf trait records of vascular plant species in the Pannonian flora with special focus on endemics and rarities. *Folia Geobot*, 55, 73–79. |
| Findurová, A. (2018). Variabilita listových znaků SLA a LDMC vybraných druhů rostlin České republiky [Variability of leaf traits SLA and LDMC in selected species of the Czech flora]. Master thesis. Masaryk University, Brno. |
| Kleyer, M., Bekker, R. m., Knevel, I. c., Bakker, J. p., Thompson, K., Sonnenschein, M., *et al.* (2008). The LEDA Traitbase: a database of life-history traits of the Northwest European flora. *Journal of Ecology*, 96, 1266–1274. |
| Weigelt, P., König, C. & Kreft, H. (2020). GIFT – A Global Inventory of Floras and Traits for macroecology and biogeography. *Journal of Biogeography*, 47, 16–43. |
| **Leaf dry matter content:** |
| Findurová, A. (2018). Variabilita listových znaků SLA a LDMC vybraných druhů rostlin České republiky [Variability of leaf traits SLA and LDMC in selected species of the Czech flora]. Master thesis. Masaryk University, Brno. |
| Kleyer, M., Bekker, R. m., Knevel, I. c., Bakker, J. p., Thompson, K., Sonnenschein, M., *et al.* (2008). The LEDA Traitbase: a database of life-history traits of the Northwest European flora. *Journal of Ecology*, 96, 1266–1274. |
| **Middle of the flowering period:** |
| Kubát, K., Hrouda, L., Chrtek, J., Kaplan, Z., Kirschner, J. & Štěpánek, J. (2002). *Klíč ke květeně České republiky [Key to the flora of the Czech Republic]*. Academia, Praha. |
| **Length of the flowering period:** |
| Kubát, K., Hrouda, L., Chrtek, J., Kaplan, Z., Kirschner, J. & Štěpánek, J. (2002). *Klíč ke květeně České republiky [Key to the flora of the Czech Republic]*. Academia, Praha. |
| **2C genome size:** |
| Chytrý, M., Danihelka, J., Kaplan, Z., Wild, J., Holubová, D., Novotný, P., *et al.* (2021). Pladias Database of the Czech Flora and Vegetation. *Preslia*, 93, 1–87. |
| Šmarda, P., Knápek, O., Březinová, A., Horová, L., Grulich, V., Danihelka, J., *et al.* (2019). Genome sizes and genomic guanine+cytosine (GC) contents of the Czech vascular flora with new estimates for 1700 species. *Presila*, 91, 117–142. |


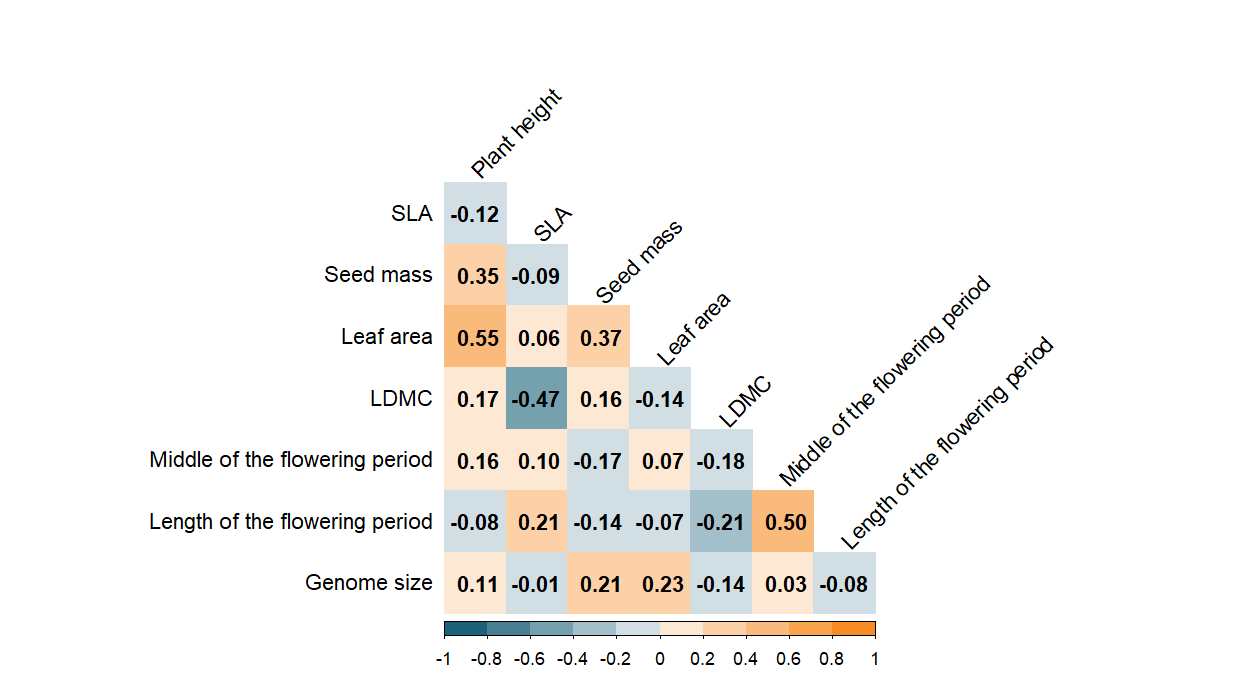


Fig. S3. Spearman correlations of species functional traits.


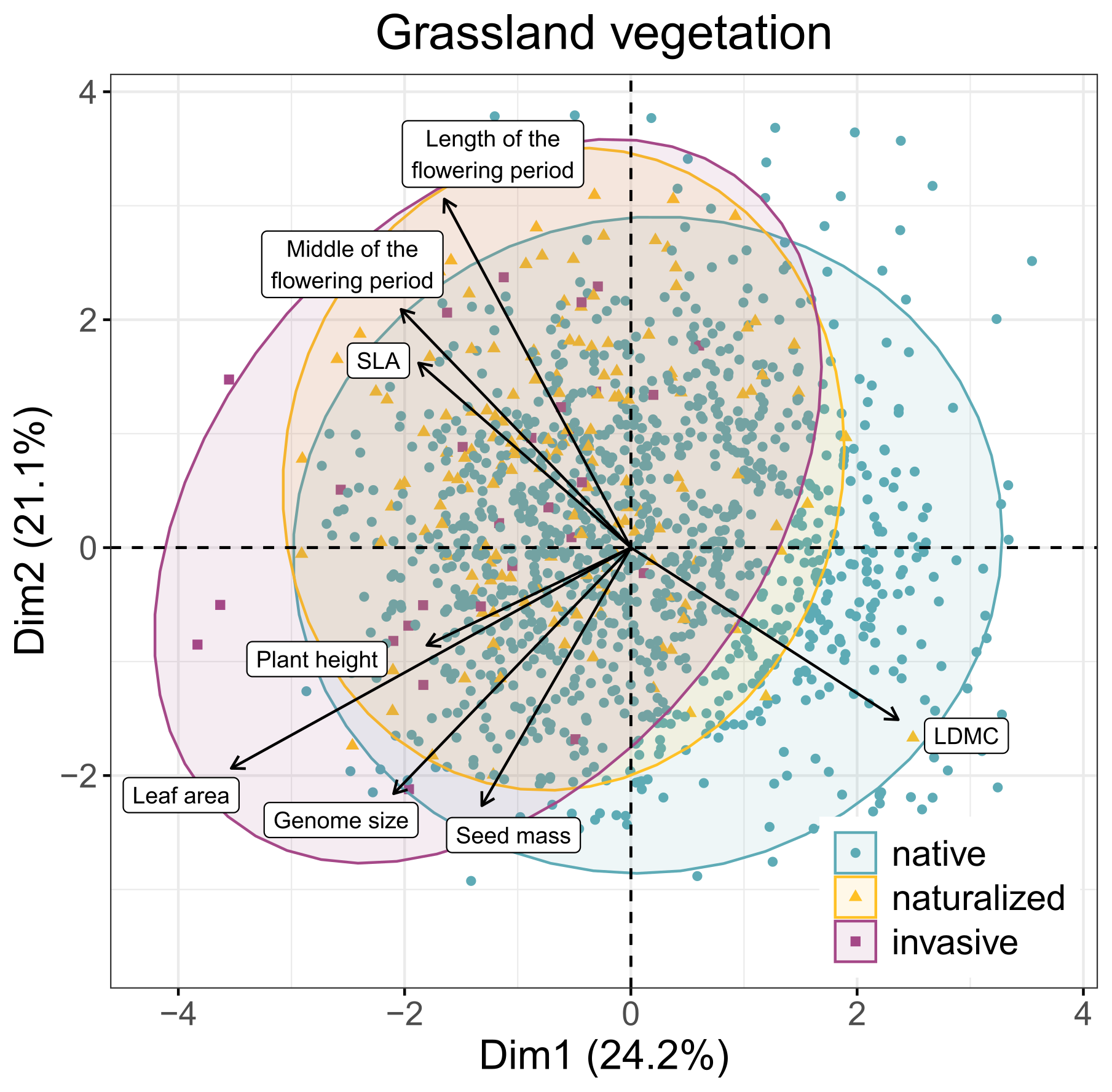


Fig. S4. The trait spectrum of grassland vegetation in the Czech Republic, visualized using principal component analysis. Native, naturalized and invasive species are represented by dots and 95% confidence ellipses. Missing trait values were imputed using the missForest method.


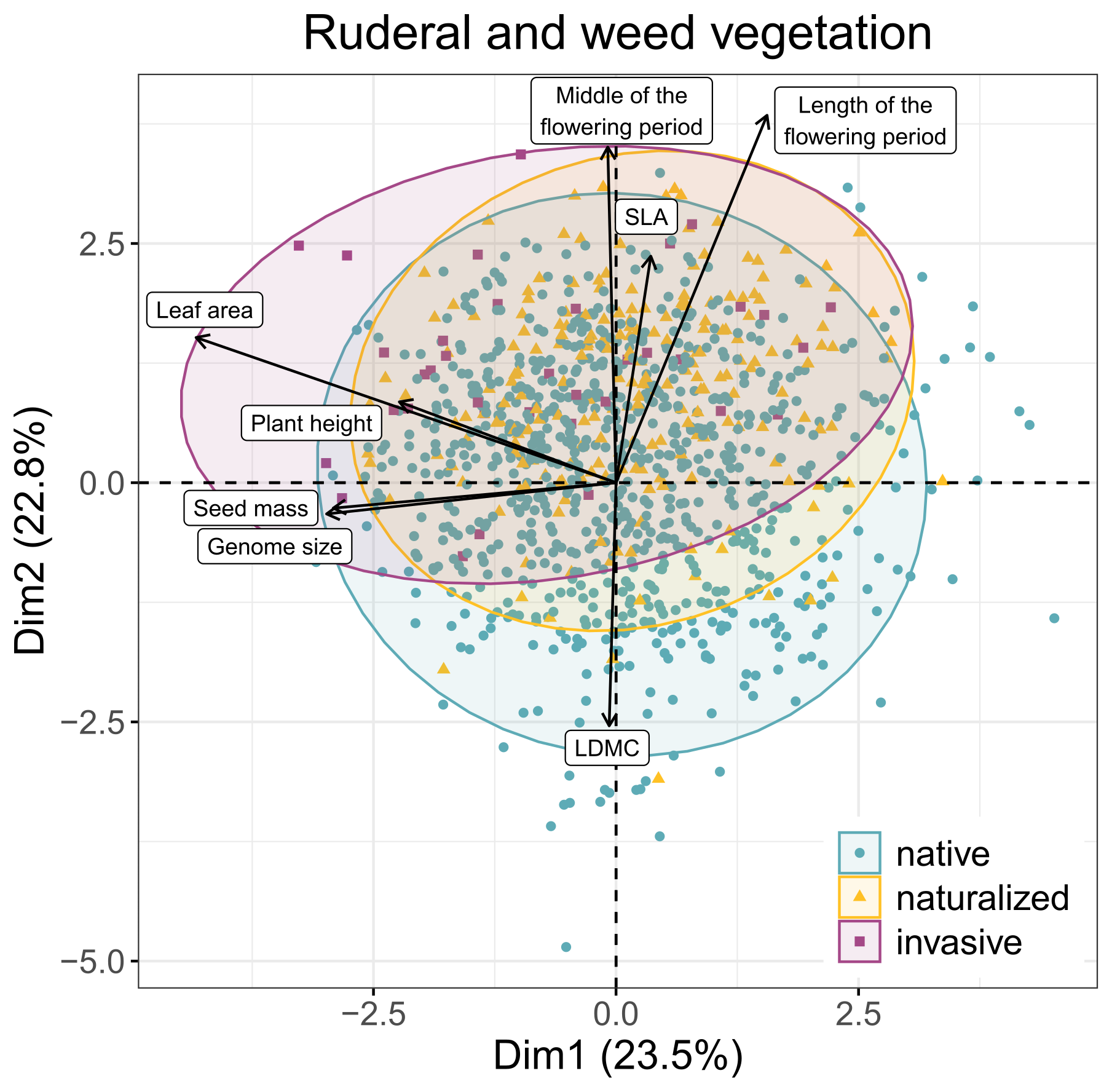


Fig. S5. The trait spectrum of ruderal and weed vegetation in the Czech Republic, visualized using principal component analysis. Native, naturalized and invasive species are represented by dots and 95% confidence ellipses. Missing trait values were imputed using the missForest method.


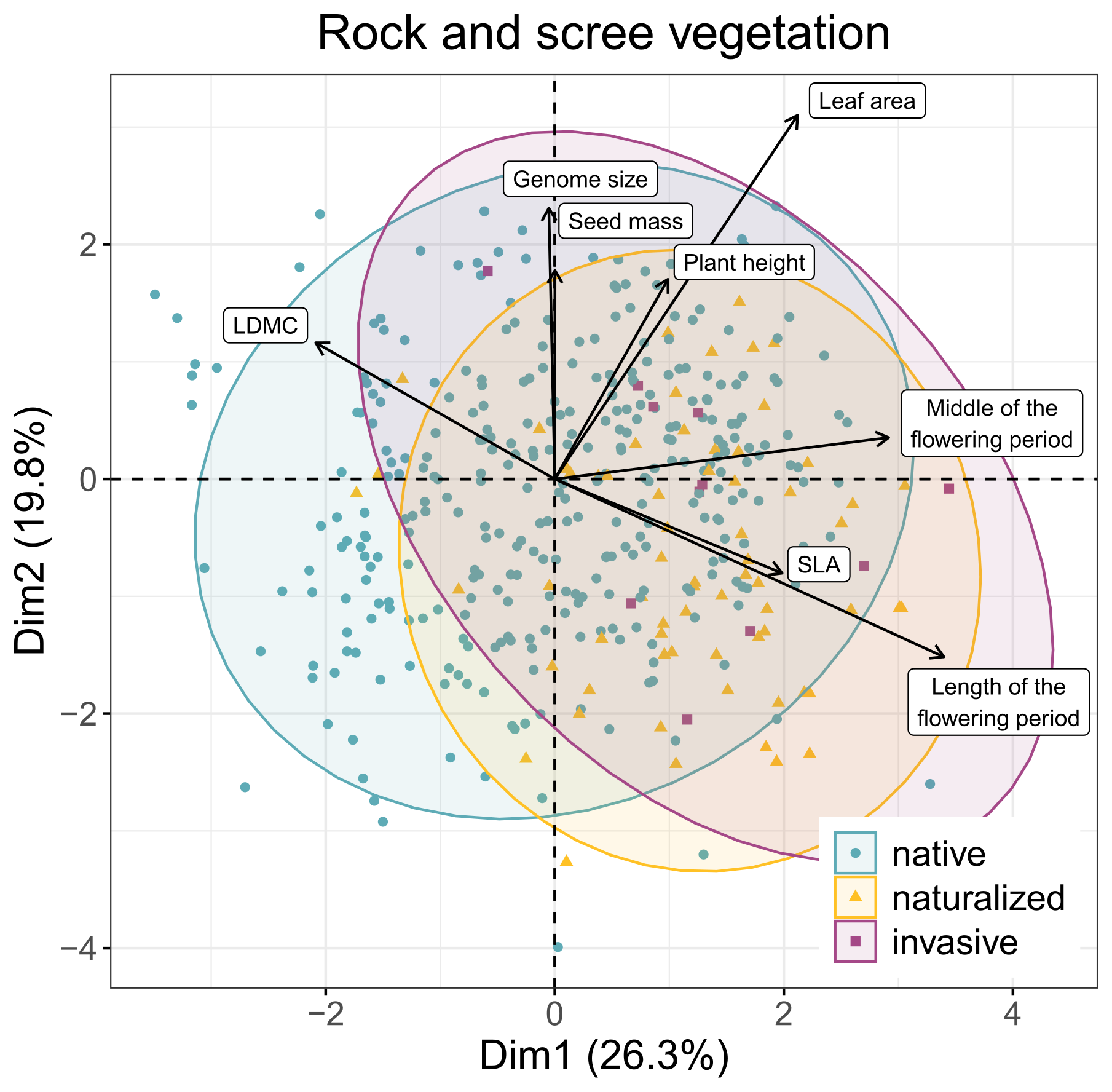


Fig. S6. The trait spectrum of rock and scree vegetation in the Czech Republic, visualized using principal component analysis. Native, naturalized and invasive species are represented by dots and 95% confidence ellipses. Missing trait values were imputed using the missForest method.


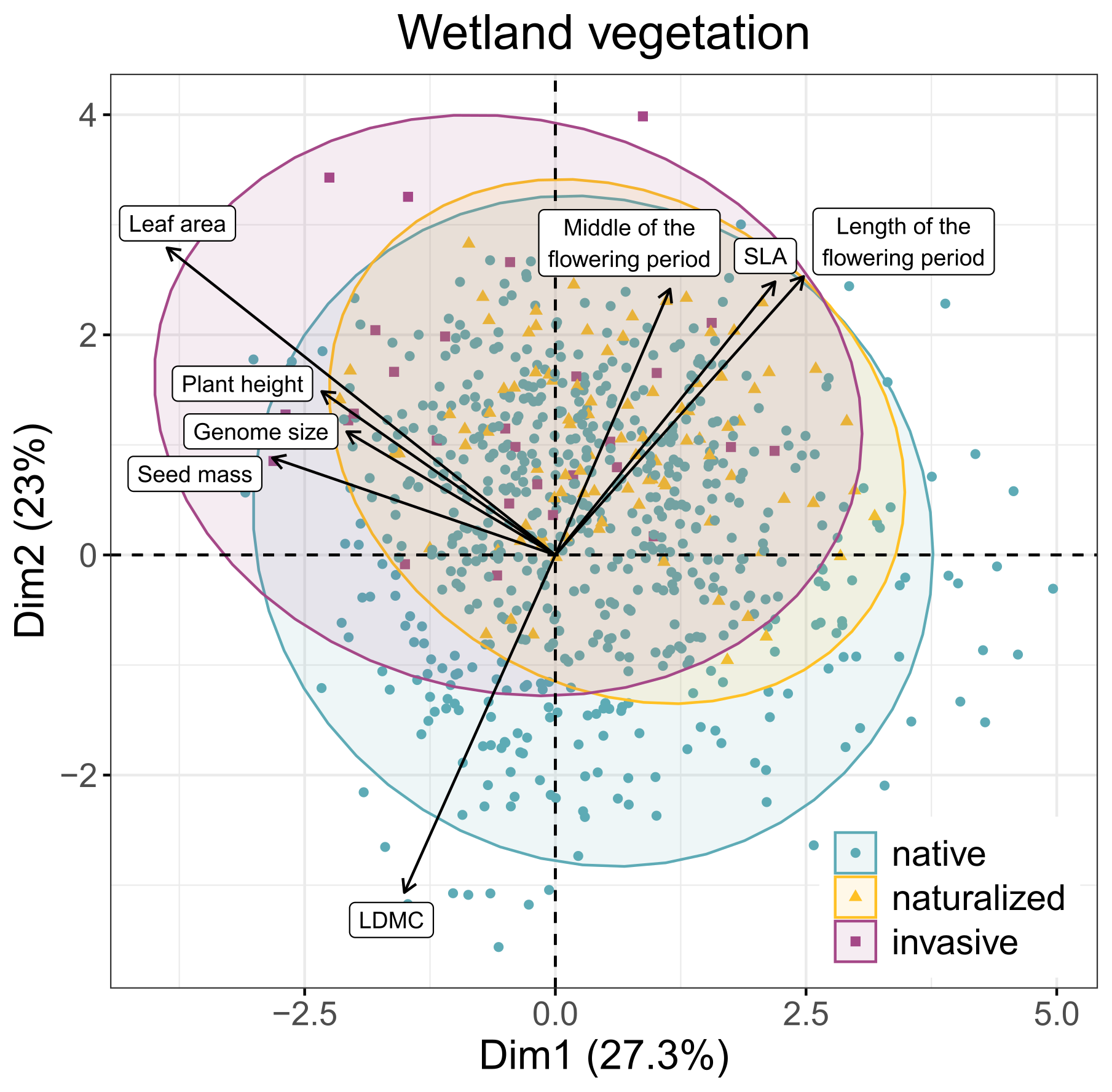


Fig. S7. The trait spectrum of wetland vegetation in the Czech Republic, visualized using principal component analysis. Native, naturalized and invasive species are represented by dots and 95% confidence ellipses. Missing trait values were imputed using the missForest method.


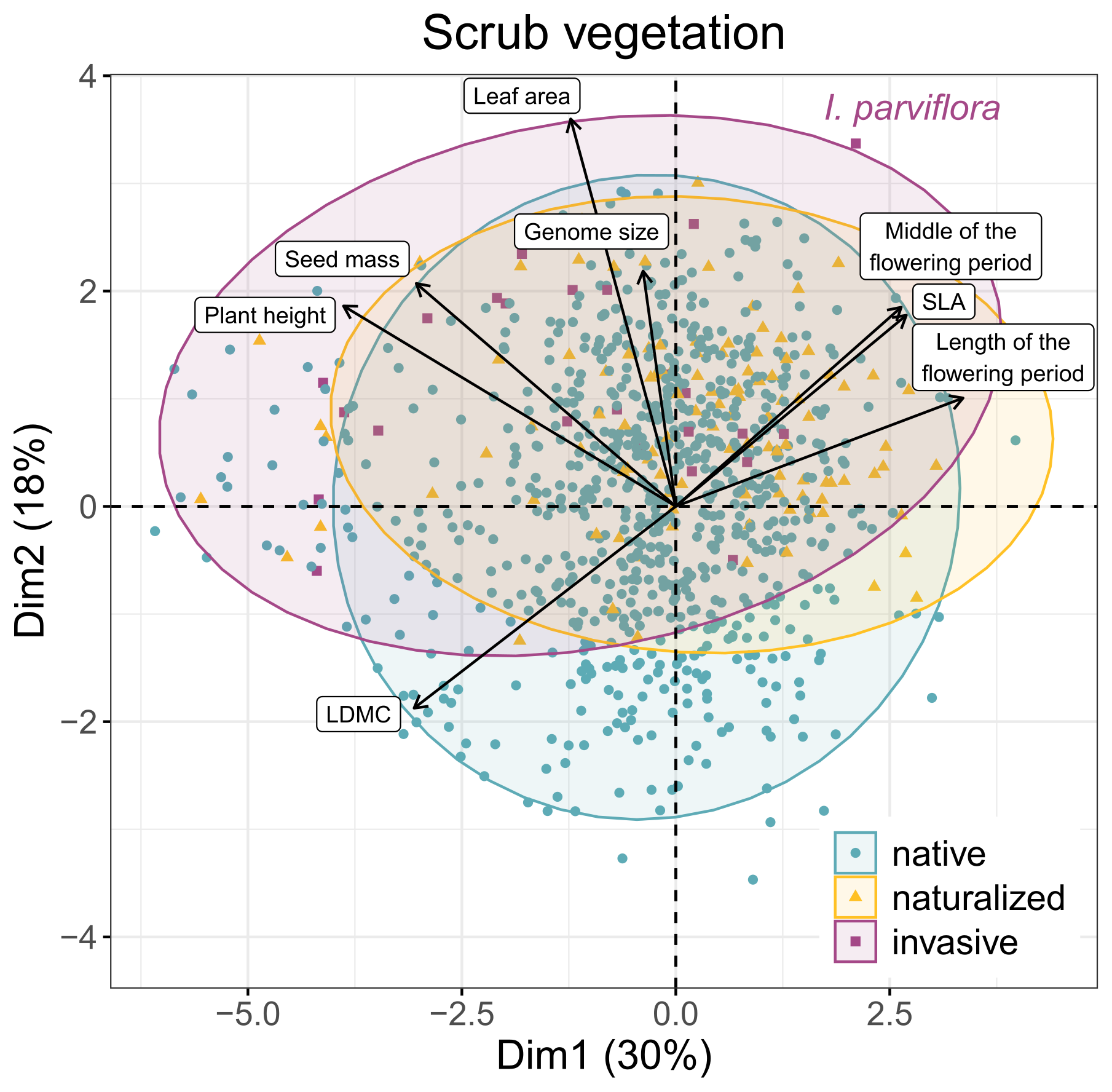


Fig. S8. The trait spectrum of scrub vegetation in the Czech Republic, visualized using principal component analysis. Native, naturalized and invasive species are represented by dots and 95% confidence ellipses. Missing trait values were imputed using the missForest method.


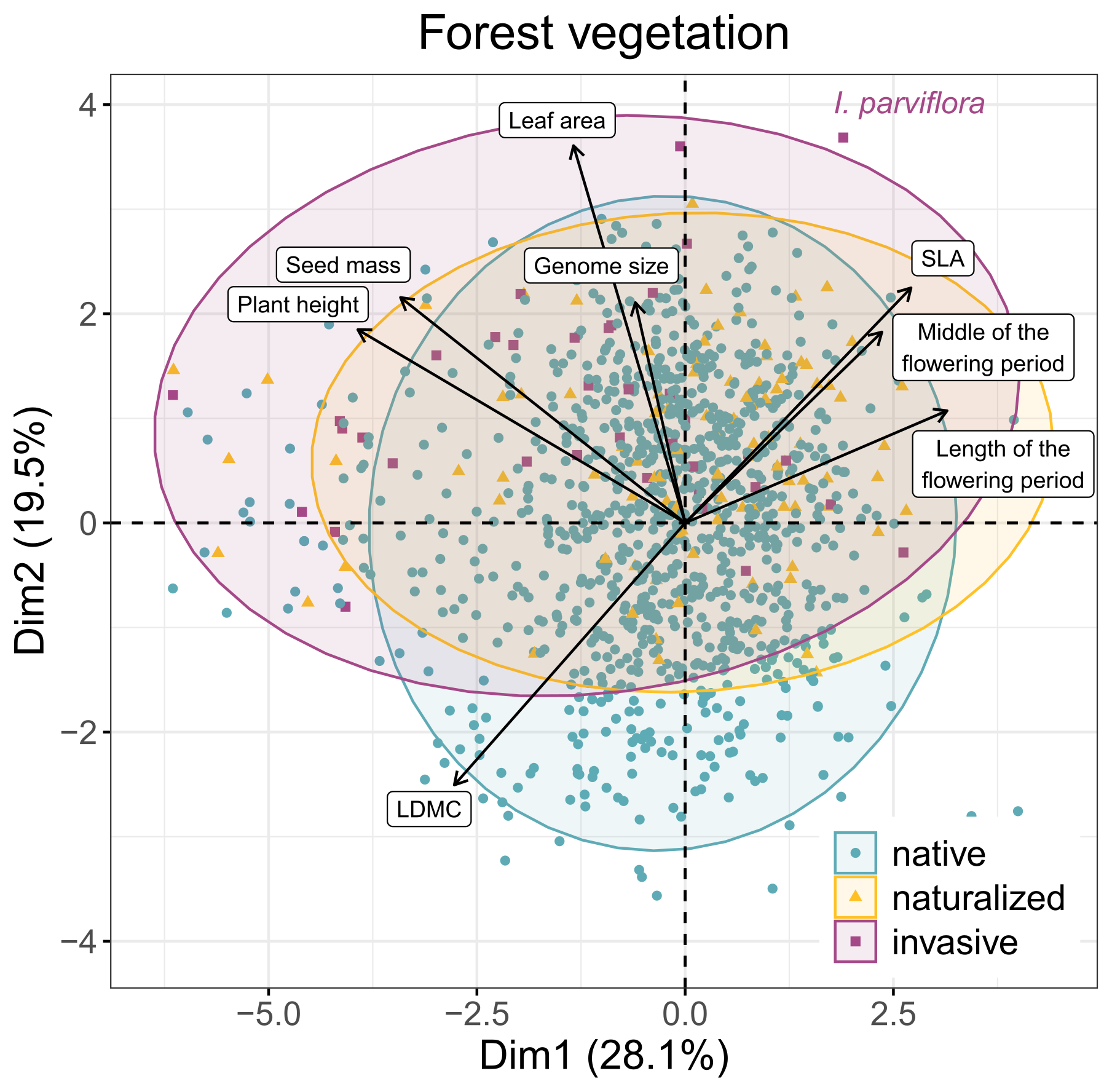


Fig. S9. The trait spectrum of forest vegetation in the Czech Republic, visualized using principal component analysis. Native, naturalized and invasive species are represented by dots and 95% confidence ellipses. Missing trait values were imputed using the missForest method.

# Appendix S2 – Supplementary methods

Table S2. Details on imputation of missing trait values. Trait values were log10-transformed and scaled to zero mean and unit variance before imputation.

| **Method** | **Reference** | **Details** |
| --- | --- | --- |
| missForest | Stekhoven, D.J. & Bühlmann, P. (2012). MissForest—non-parametric missing value imputation for mixed-type data. *Bioinformatics*, 28, 112–118. | The first 10 eigenvectors obtained from phylogenetic eigenvector analysis (Peres-Neto 2006) were used as additional predictors to improve the estimation of missing trait values (Penone *et al.* 2014). Details on their calculation are provided in Divíšek *et al*. (2018). missForest settings: maxiter = 10, ntree = 100, mtry = 4. |
| Multivariate Imputation by Chained Equations (MICE) with the predictive mean matching (PMM) | van Buuren, S. & Groothuis-Oudshoorn, K. (2011). mice: Multivariate Imputation by Chained Equations in R. *Journal of Statistical Software*, 45, 1–67. | The first 10 eigenvectors obtained from phylogenetic eigenvector analysis (Peres-Neto 2006) were used as additional predictors to improve the estimation of missing trait values (Penone *et al.* 2014). Details on their calculation are provided in Divíšek *et al*. (2018). Because MICE-PMM is a process of multiple imputations we used 10 iterations and computed their mean. |
| Phylopars | Goolsby, E., Bruggeman, J. & Ane, C. (2024). _Rphylopars: Phylogenetic Comparative Tools for Missing Data and Within-Species Variation_. R package version 0.3.10. | The phylogenetic tree from Jin & Qian (2022) assuming a Brownian motion evolutionary model was used for imputation. Phylopars settings: pheno_error = TRUE, phylo_correlated = TRUE, pheno_correlated = TRUE. |


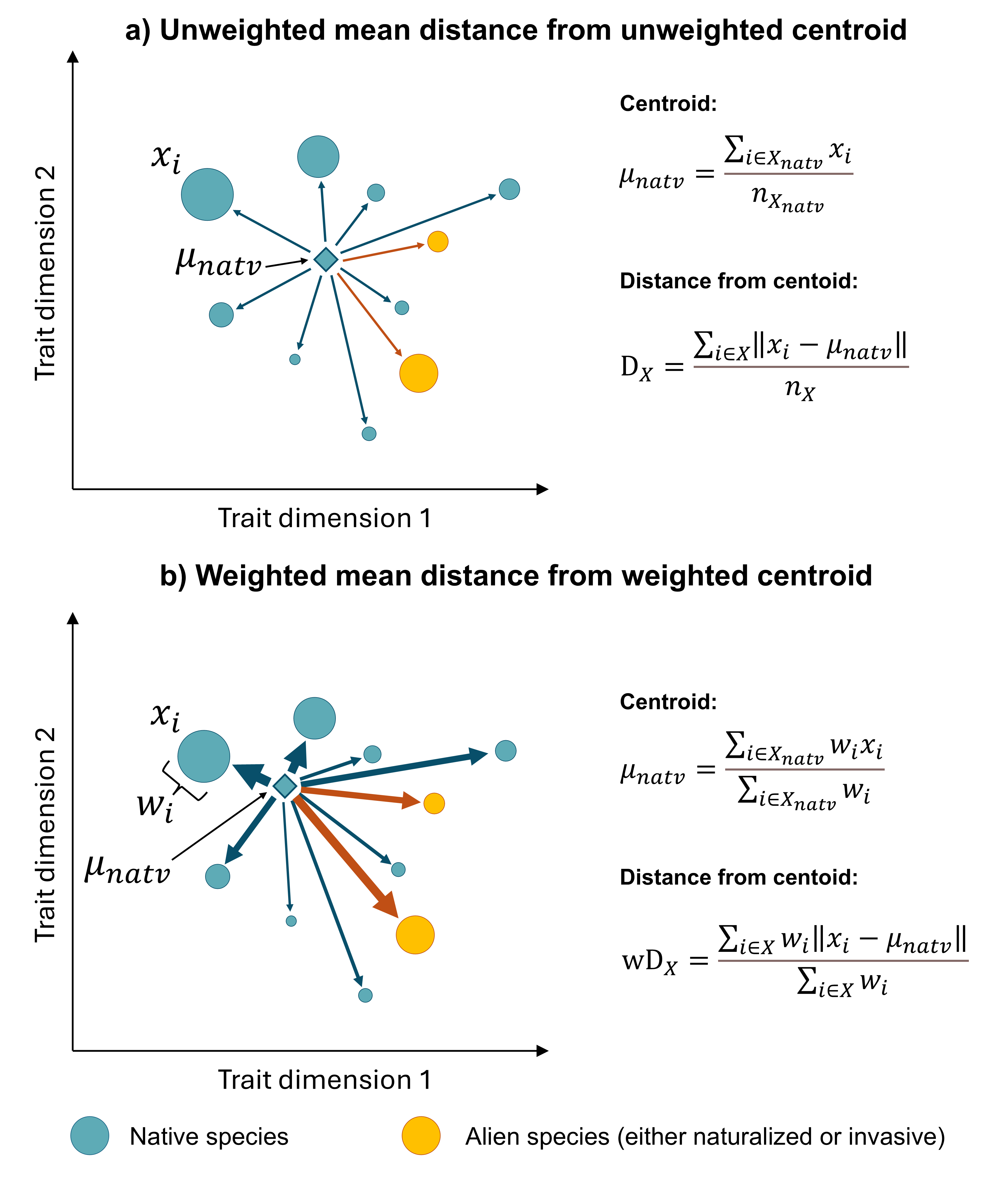


Fig. S10. An example showing how the mean distance of a community fraction from the native center of the trait space in each vegetation plot is calculated. The $\boldsymbol{n}$ individual species in a two-dimensional trait space are represented by colored circles whose sizes are proportional to their abundances (i.e. covers in the vegetation plot). Vector $\boldsymbol{x}_{\boldsymbol{i}}$ represents the position of species $\boldsymbol{i}$ from species group $\boldsymbol{X}$ (i.e. native, naturalized or invasive), vector $\boldsymbol{\mu}_{\boldsymbol{natv}}$is the centroid of native species (rhombus) and $\boldsymbol{w}_{\boldsymbol{i}}$ is the abundance of species $\boldsymbol{i}$. $\left\| \boldsymbol{\cdot} \right\|$ denotes Euclidean norm. Distances of individual species from this centroid (arrows) are scaled to relative values. In panel (a), species abundances are not taken into account, so that the unweighted mean distance of a species group $\boldsymbol{X}$ (D) is calculated from the unweighted centroid of the native species. In panel (b), square-root-transformed species abundances are used as weights to calculate the weighted centroid of the native species. The distances from this centroid are then weighted by transformed species covers (indicated by arrow thickness) resulting in the weighed mean distance from the weighed native centroid (wD). This approach is analogous to the calculation of the functional dispersion index introduced by Laliberté & Legendre (2010).


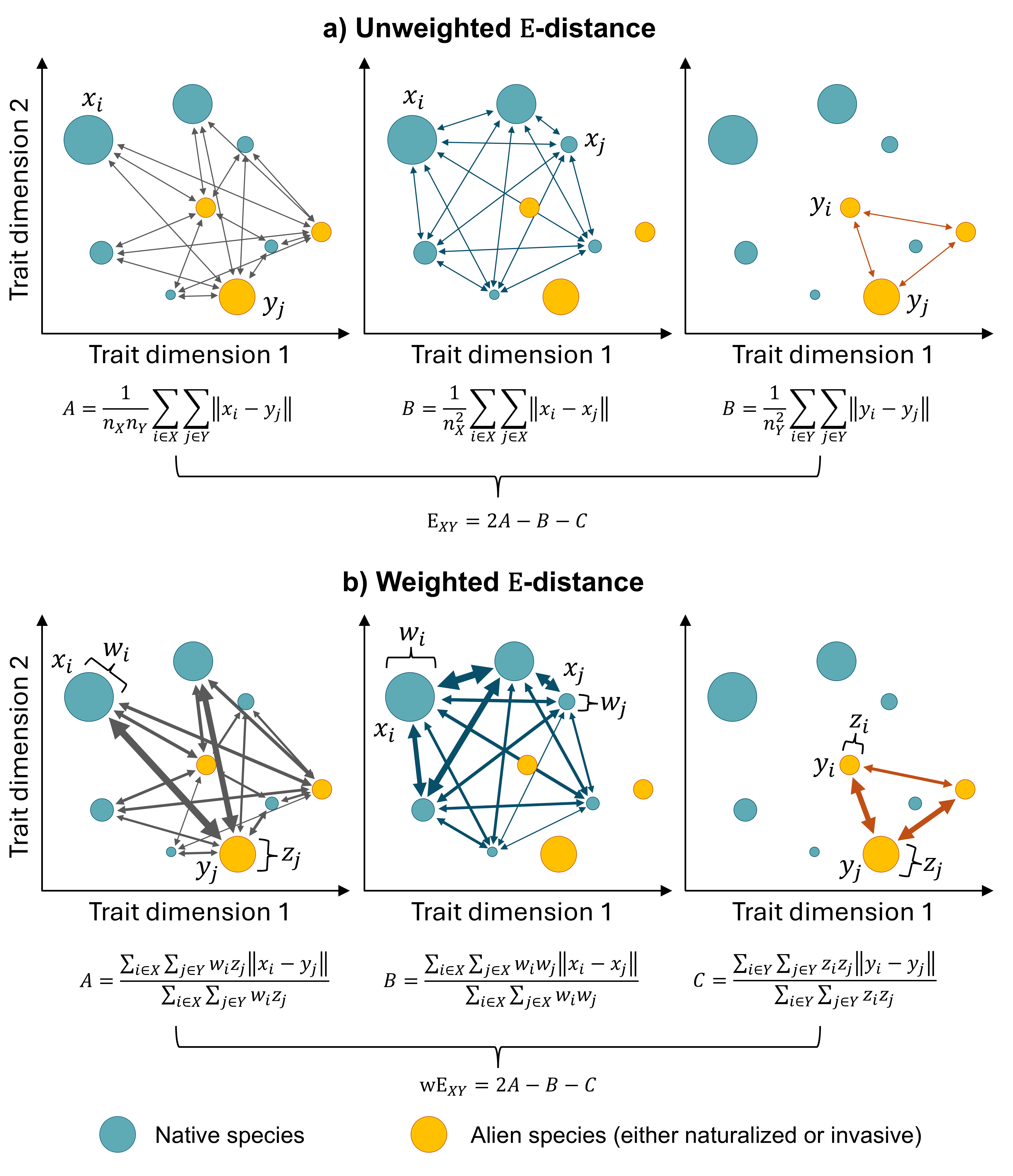


Fig. S11. An example showing how the E-distance for each vegetation plot is calculated. The $\boldsymbol{n}$ individual species in a two-dimensional trait space are represented by colored circles whose sizes are proportional to their abundances (i.e. covers in the vegetation plot). Their pairwise distances (arrows) are scaled to relative values. Vectors $\boldsymbol{x}_{\boldsymbol{i}}$ and $\boldsymbol{y}_{\boldsymbol{j}}$ represent the positions of species $\boldsymbol{i}$ and $\boldsymbol{j}$ from species groups $\boldsymbol{X}$ and $\boldsymbol{Y}$, respectively. $\boldsymbol{w}_{\boldsymbol{i}}$ and $\boldsymbol{z}_{\boldsymbol{j}}$ are the abundances of species $\boldsymbol{i}$ and $\boldsymbol{j}$. $\left\| \boldsymbol{\cdot} \right\|$ denotes Euclidean norm. In the second ($\boldsymbol{B}$) and third ($\boldsymbol{C}$) term, if $\boldsymbol{i}\mathbf{=}\boldsymbol{j}$, then $\left\| \boldsymbol{x}_{\boldsymbol{i}}\mathbf{-}\boldsymbol{x}_{\boldsymbol{j}} \right\|\mathbf{=0}$ and $\left\| \boldsymbol{y}_{\boldsymbol{i}}\mathbf{-}\boldsymbol{y}_{\boldsymbol{j}} \right\|\mathbf{=0}$, respectively. In panel (a), species abundances are not considered, so that the unweighted mean pairwise distance between groups $\boldsymbol{X}$ and $\boldsymbol{Y}$ (term $\boldsymbol{A}$) and within each of these groups (terms $\boldsymbol{B}$ and $\boldsymbol{C}$) is calculated, resulting in the unweighted E-distance ($\mathbf{E}$) as defined by (Rizzo & Székely 2010, 2016). In panel (b), square-root-transformed species abundances are used as weights to calculate the weighted mean pairwise distance between groups $\boldsymbol{X}$ and $\boldsymbol{Y}$, and within each of these groups (terms $\boldsymbol{B}$ and $\boldsymbol{C}$), resulting in the weighted E-distance ($\mathbf{wE}$).


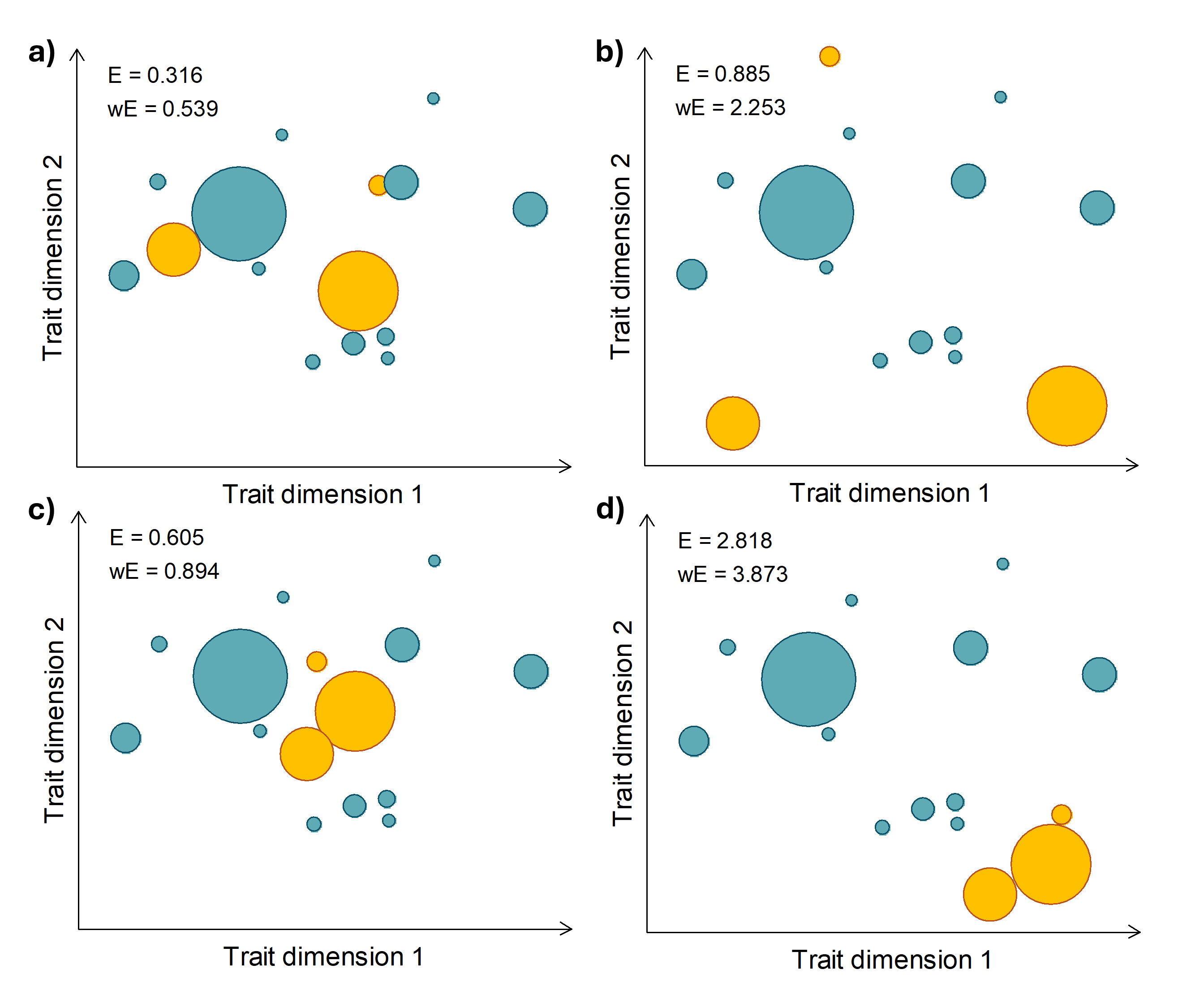


Fig. S12. Examples of unweighted (E) and weighted (wE) E-distance values calculated for four different communities. Twelve native (cyan) and three alien species (gold) in a two-dimensional trait space are represented by circles whose sizes are proportional to their abundances (i.e. covers in the vegetation plot). (a) If native and alien species occupy similar positions in the trait space (high overlap), the resulting E and wE values are small. (b) If alien species are dispersed at the edge of the trait space, the species overlap is low and the resulting E and wE values are high. (c) Similar to (b), but here the alien species cluster in the center of the trait space, while the native species are dispersed at the edge. In this case, the resulting E and wE values are high, but not as high as in (b) because the whole community is more clustered. (c) If alien species separate from native species and occupy a specific region of the trait space (low overlap), the resulting E and wE values are highest.

Table S3. List of R packages used in the analyses.

| **Package name** | **Reference** |
| --- | --- |
| coin | Hothorn, T., Hornik, K., van de Wiel, M.A. & Zeileis, A. (2008). Implementing a class of permutation tests: The coin package. *Journal of Statistical Software*, 28, 1–23. |
| energy | Rizzo, M. & Szekely, G. (2024). _energy: E-Statistics: Multivariate inference via the energy of data_. R package version 1.7-12. |
| FD | Laliberté, E., Legendre, P. & B. Shipley. (2014). FD: measuring functional diversity from multiple traits, and other tools for functional ecology. R package version 1.0-12.3. |
| GIFT | Denelle, P. & Weigelt, P. (2024). _GIFT: Access to the Global Inventory of Floras and Traits (GIFT)_. R package version 1.3.3. |
| mice | van Buuren, S. & Groothuis-Oudshoorn, K. (2011). mice: Multivariate Imputation by Chained Equations in R. *Journal of Statistical Software*, 45, 1–67. |
| missForest | Stekhoven, D.J. & Bühlmann, P. (2012). MissForest—non-parametric missing value imputation for mixed-type data. *Bioinformatics*, 28, 112–118. |
| rpart | Therneau, T. & Atkinson, B. (2022). rpart: Recursive Partitioning and Regression Trees. R package version 4.1.16. |
| Rphylopars | Goolsby, E., Bruggeman, J. & Ane, C. (2024). _Rphylopars: Phylogenetic Comparative Tools for Missing Data and Within-Species Variation_. R package version 0.3.10. |
| rstatix | Kassambara, A. (2023). _rstatix: Pipe-Friendly Framework for Basic Statistical Tests_. R package version 0.7.2. |
| vegan | Oksanen, J., Simpson, G.L., Blanchet, F.G., Kindt, R., Legendre, P., Minchin, P.R., *et al*. (2022). vegan: Community Ecology Package. R package version 2.6-4. |
| V.PhyloMaker2 | Jin Y (2025). _V.PhyloMaker2: Make phylogenetic hypotheses for vascular plants._. R package version 0.1.0. |

# Appendix S3 – Distances (D) within individual traits

Results in this appendix are based on the data from the missForest imputation and the simulations of the null model 3.


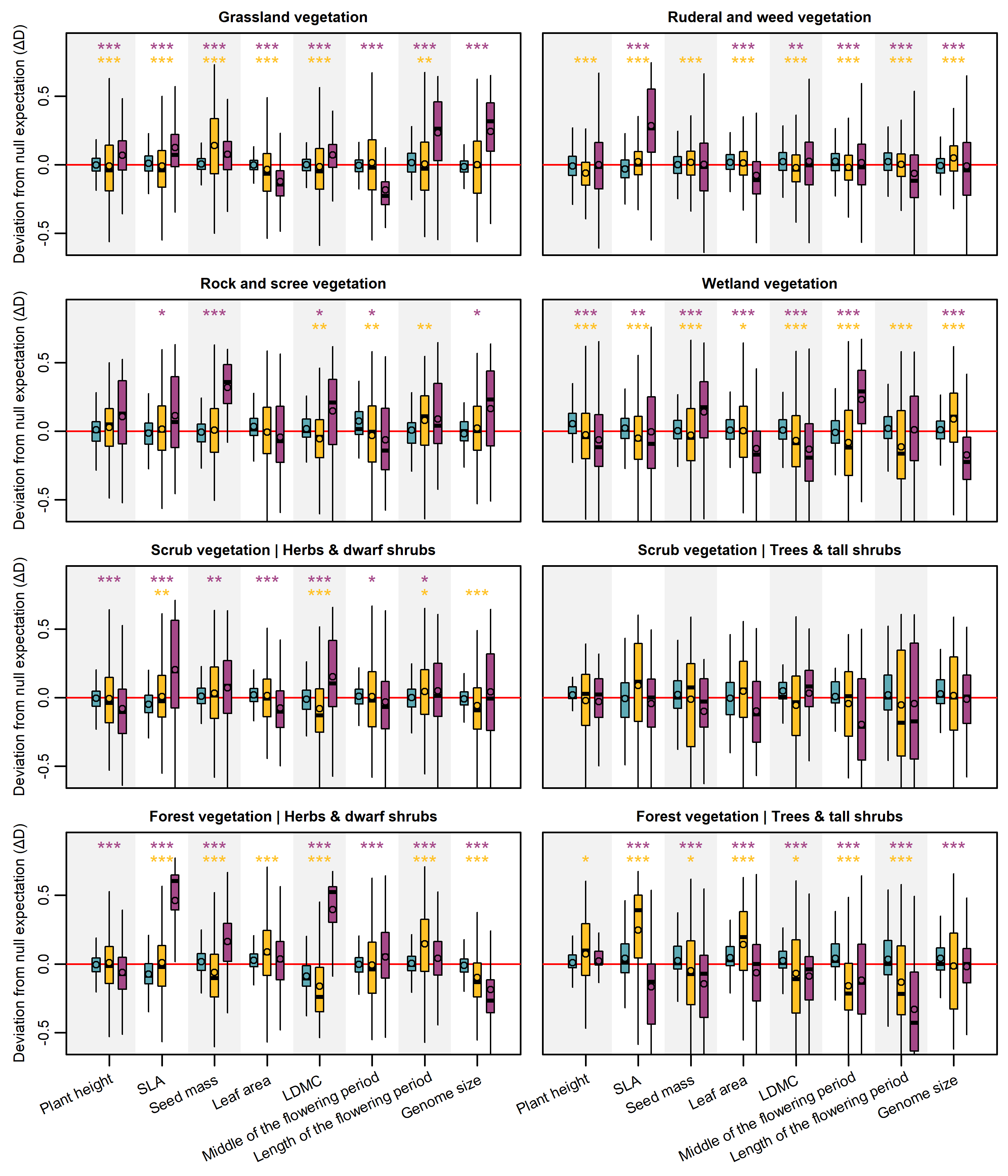


Fig. S13. Distances of native (cyan), naturalized (gold) and invasive (magenta) community fractions from the mean trait value of native species in each vegetation plot. The data from the missForest imputation and the simulations of the null model 3 were used. Distances are expressed as the deviation (ΔD) from the mean expected distance for community fractions of the same size simulated by the weighted random drawing of native species from habitat species pools (null model 3). Positive values indicate a tendency towards overdispersion (greater distance from the mean native trait value than expected for the simulated community), while negative values indicate tendency towards clustering (shorter distance from the mean native trait value than expected). Within-habitat differences between native and naturalized species (gold font) and between native and invasive species (magenta font) were tested using Wilcoxon signed-rank test for paired samples and resulting *p*-values were adjusted using Benjamini & Hochberg’s (1995) method: *** *p* < 0.001, ** 0.001 ≤ *p* < 0.01, * 0.01 ≤ *p* < 0.05. The thick horizontal line in each box indicates the median, while the dot indicates the mean. The bottom and top of each box indicate the 25th and 75th percentiles, respectively, and the vertical lines (whiskers) represent either the maximum/minimum value or 1.5 × interquartile range, whichever is closer to the mean.


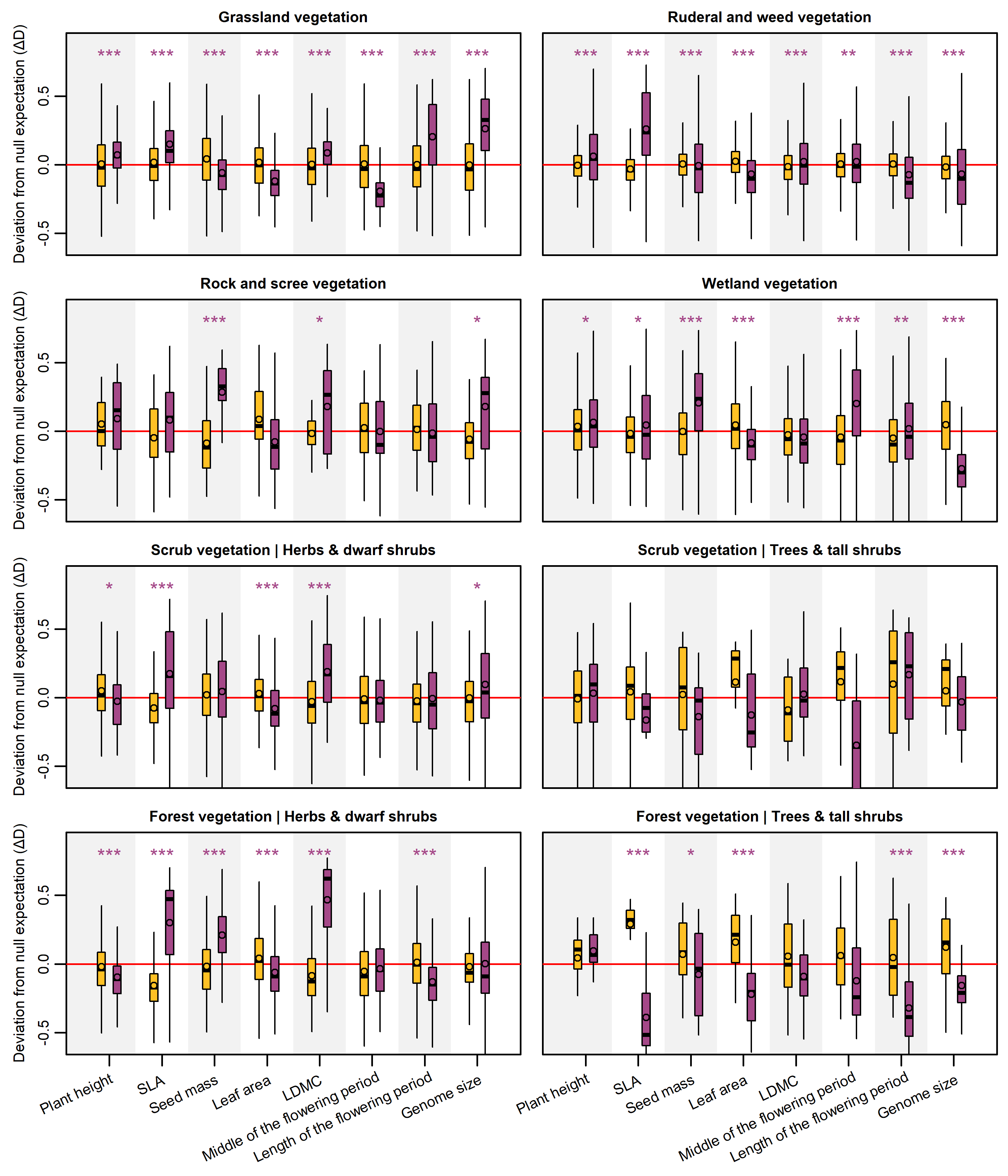


Fig. S14. Distances of naturalized (gold) and invasive (magenta) community fractions from the mean trait value of native species in each vegetation plot. The data from the missForest imputation and the simulations of the null model 3 were used. Distances are expressed as the deviation from the mean expected distance for community fractions simulated by the weighted random drawing of naturalized species from habitat species pools. For details, see Fig. S13.

# Appendix S4 – Results after excluding *Impatiens parviflora*

Results in this appendix are based on the data from the missForest imputation and the simulations of the null model 3.


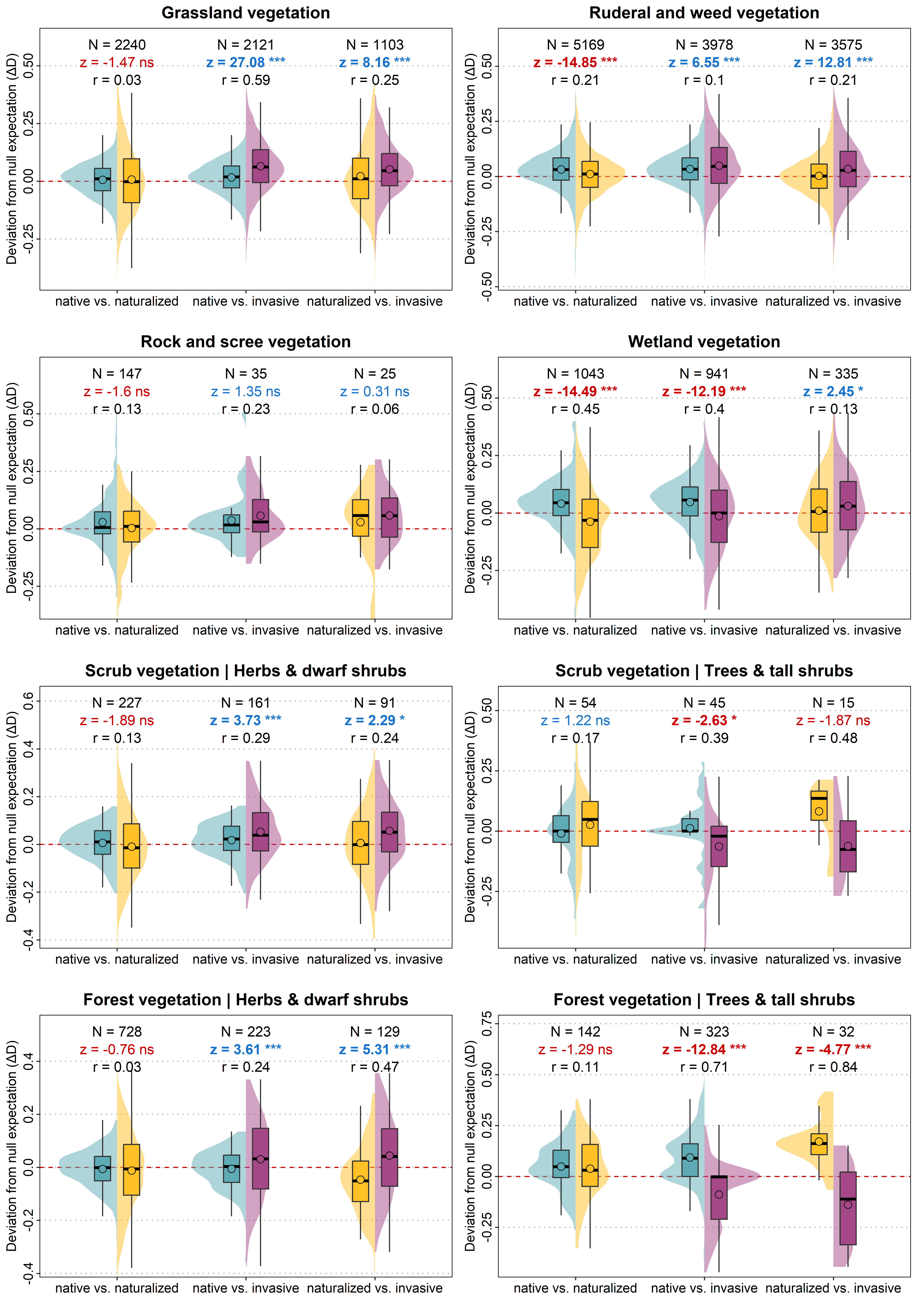


Fig. S15. Distances of native (cyan), naturalized (gold) and invasive (magenta) community fractions from the native center of the eight-dimensional trait space in each vegetation plot after removing *Impatiens parviflora*. The data from the missForest imputation and the simulations of the null model 3 were used. Distances are expressed as the deviation (ΔD) from the mean expected distance for community fractions of the same size simulated by the weighted random drawing of native species (or naturalized species for the ‘naturalized vs. invasive’ comparison) from the habitat species pools (null model 3). Positive values indicate a tendency towards overdispersion (greater distance from the center of the native trait space than expected for the simulated community), while negative values indicate tendency towards clustering (shorter distance from the center than expected). N is the number of plots, z is the statistical value of the Wilcoxon signed-rank test for paired samples. *P*-values were adjusted following Benjamini & Hochberg (1995) method: *** *p* < 0.001, ** 0.001 ≤ *p* < 0.01, * 0.01 ≤ *p* < 0.05, ns *p* ≥ 0.05. Statistically significant results (*p* < 0.05) are in bold. Red and blue fonts indicate negative and positive difference, respectively. The effect size r (in absolute value) associated with the Wilcoxon test is considered small when r < 0.3, medium when 0.3 ≤ r < 0.5, and large when r ≥ 0.5. The thick horizontal line in each box indicates the median, while the dot indicates the mean. The bottom and top of each box indicate the 25th and 75th percentiles, respectively, and the vertical lines (whiskers) represent either the maximum/minimum value or 1.5 × interquartile range, whichever is closer to the mean.


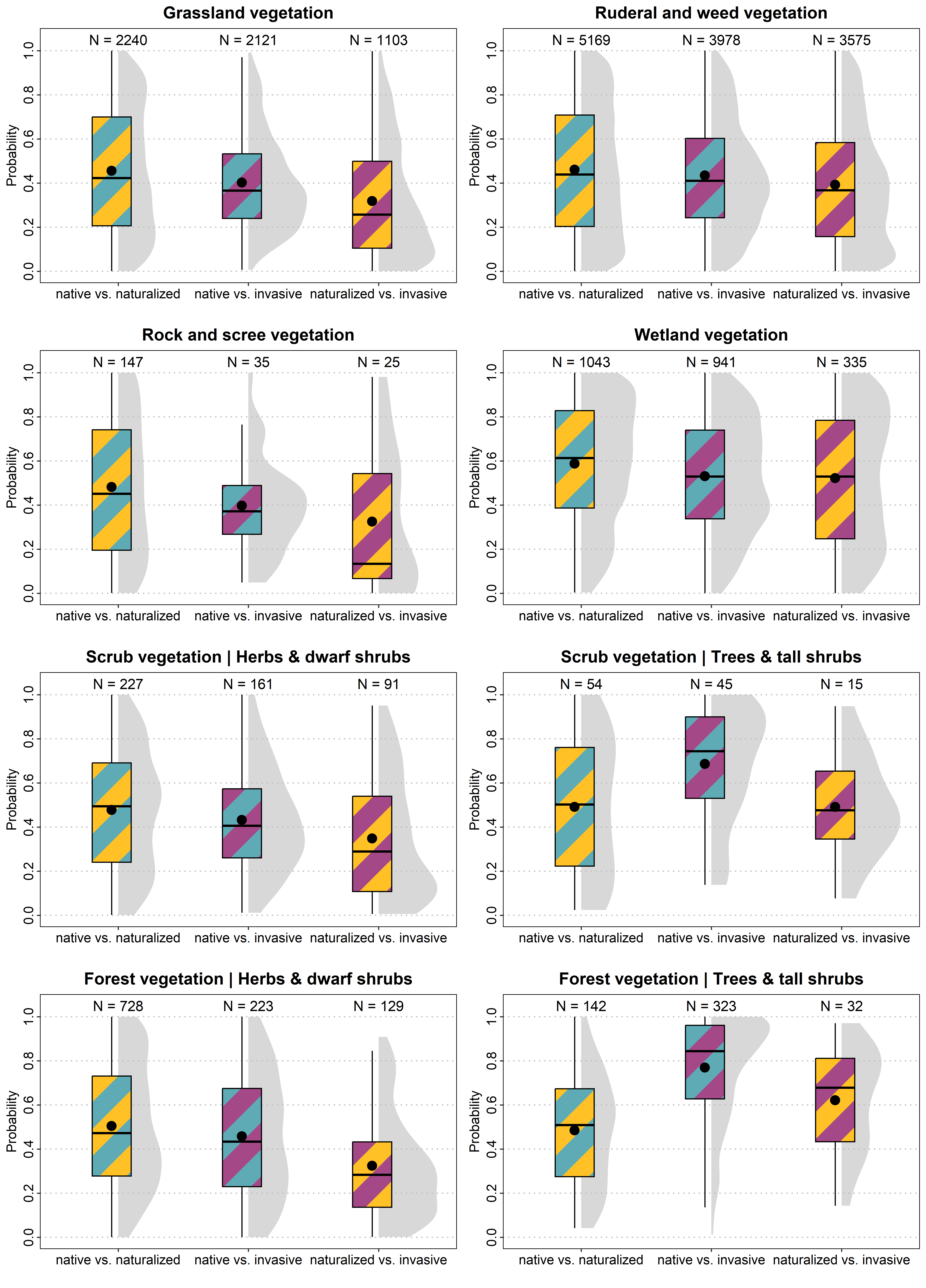


Fig. S16. Probability of overlap between native and naturalized species (cyan/gold), native and invasive species (cyan/magenta) and naturalized and invasive species (gold/magenta) in the eight-dimensional trait space of each plot after removing *Impatiens parviflora*. The data from the missForest imputation and the simulations of the null model 3 were used. Probability was calculated by comparing the observed overlap value, expressed by E-distance, with the distribution of 999 simulated overlap values for two community fractions with corresponding sizes and traits of only native (for ‘native vs naturalized’ and ‘native vs invasive’ comparisons) or naturalized species (for ‘naturalized vs invasive’ comparison). Probability values approaching zero indicate that the observed overlap is smaller (E-distance is larger) than in simulated communities while value of one indicates larger observed overlap (smaller E-distance) than in all simulated communities. N is the number of plots in which native and alien species were compared.


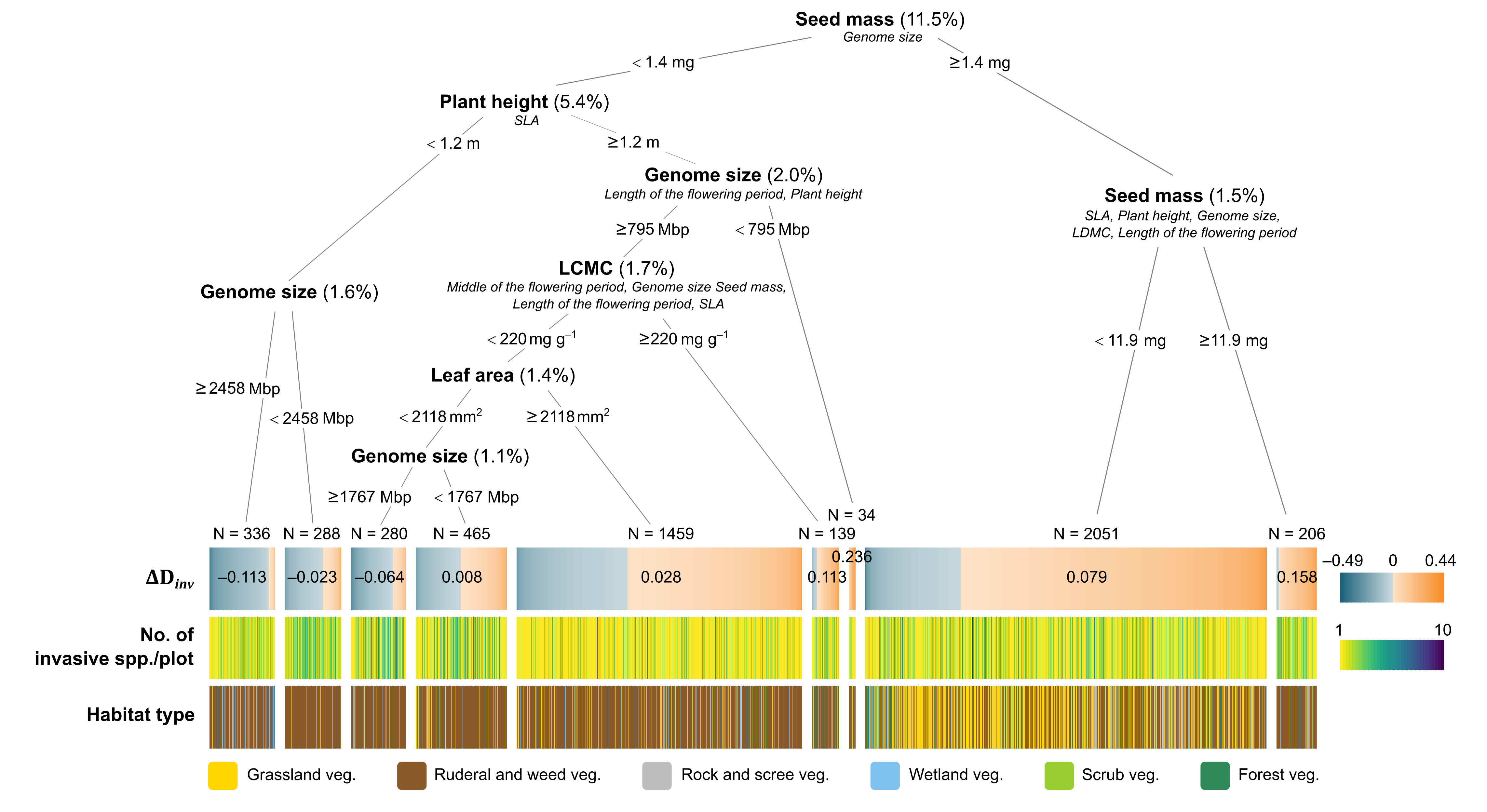


Fig. S17. Decision tree for functional traits contributing to the clustering ($\boldsymbol{\Delta D}\boldsymbol{<0}$; indicated by blue-gray color scale) or dispersion ($\boldsymbol{\Delta D}\boldsymbol{>0}$; orange color scale) of invasive community fractions when the distance from the trait space center expected for a naturalized community fraction of the same size is accounted for. *Impatiens parviflora*, tall shrubs and trees were excluded. The data from the missForest imputation and the simulations of the null model 3 were used. The primary splitter variable at each node is shown in bold and its split value is indicated below the node. The numbers in parentheses indicate the percentage variance explained by the splitting. Surrogates (i.e. variables that assign at least 90% of the cases to the same group as the primary splitter) are indicated in smaller italic letters below the primary splitter. The mean $\boldsymbol{\Delta D}_{\boldsymbol{inv}}$ and the number of community fractions (N) included in each terminal node are shown within and above the uppermost color scale, respectively. The total variance explained by the tree is 26.2%.

# Appendix S5 – Results for weighted distance metrics (wD and wE)

Results in this appendix are based on the data from the missForest imputation and the simulations of the null model 3. Square-root-transformed species percentage covers in the vegetation plots were used as weights to calculate the weighted D (wD) and E-distance (wE; see Appendix S2).


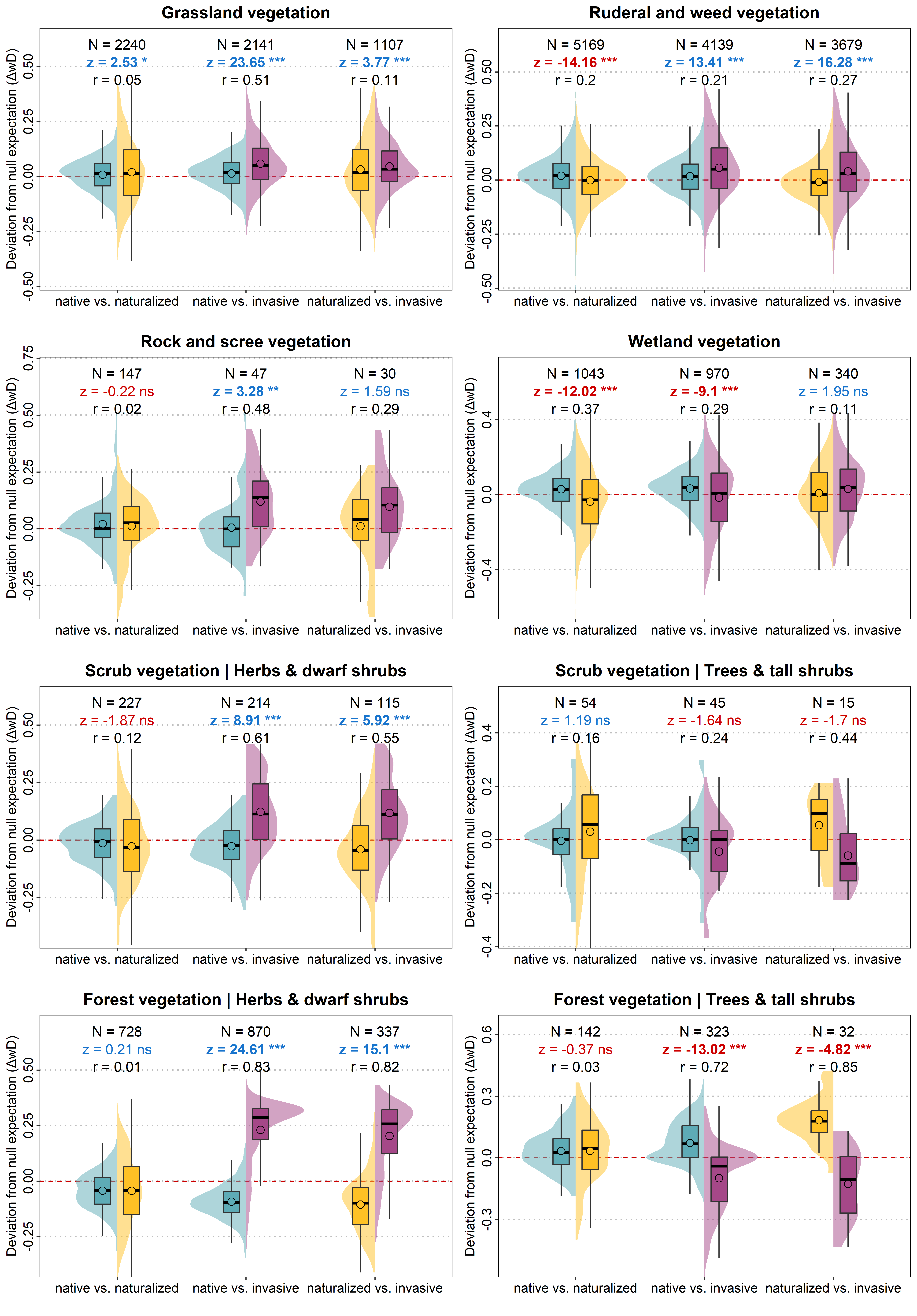


Fig. S18. Weighted distances of native (cyan), naturalized (gold) and invasive (magenta) community fractions from the native center of the eight-dimensional trait space in each vegetation plot. The data from the missForest imputation and the simulations of the null model 3 were used. Square-root-transformed species percentage covers in vegetation plots were used as weights to calculate the weighted mean distance of each community fraction from the weighted centroid of native species (wD; see Fig. S10). Distances are expressed as the deviation (ΔwD) from the mean expected distance for community fractions of the same size simulated by the weighted random drawing of native species (or naturalized species for the ‘naturalized vs. invasive’ comparison) from the habitat species pools (null model 3). For details, see Fig S15.


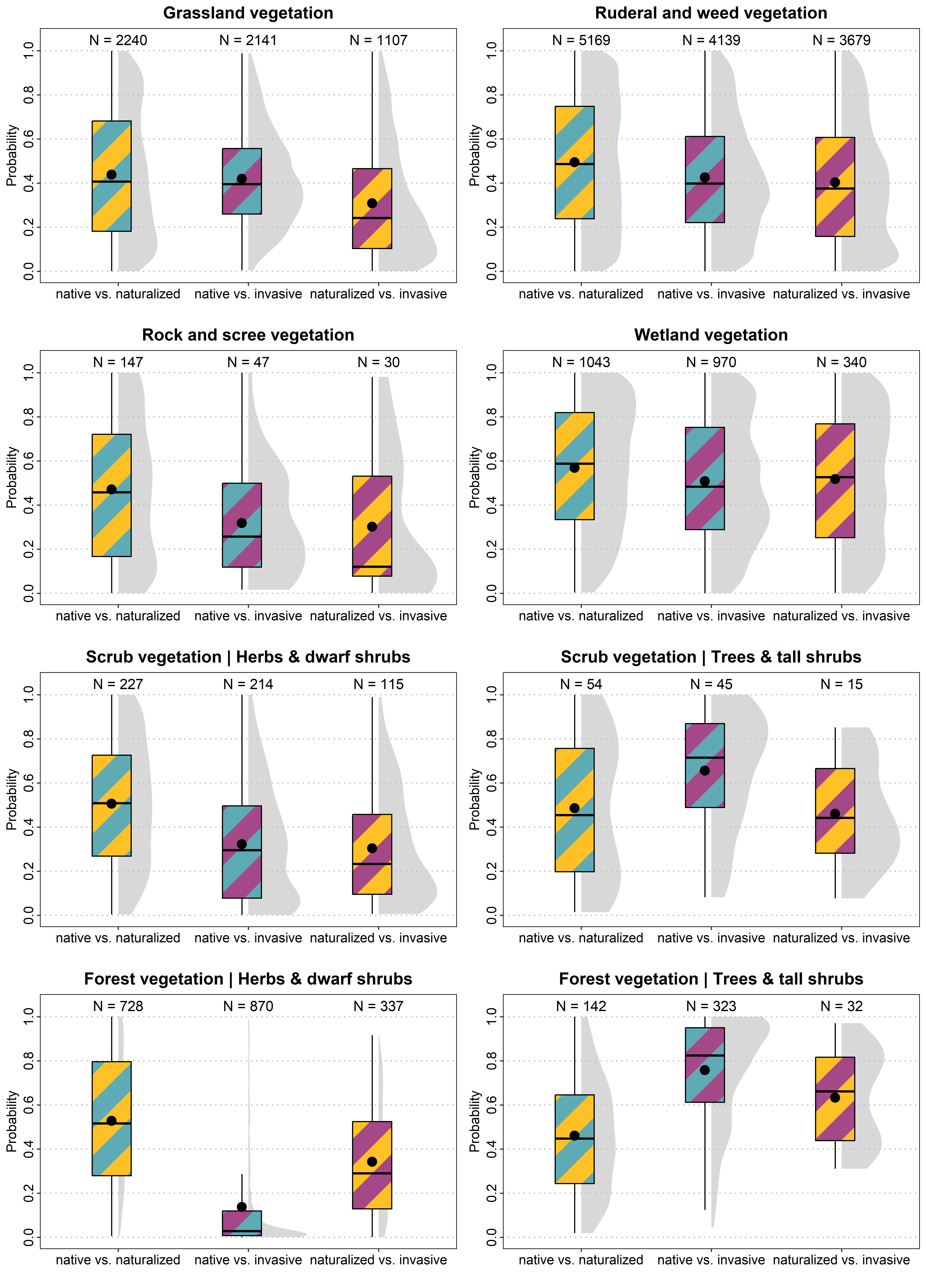


Fig. S19. Probability of overlap between native and naturalized species (cyan/gold), native and invasive species (cyan/magenta) and naturalized and invasive species (gold/magenta) in the eight-dimensional trait space of each plot. The data from the missForest imputation and the simulations of the null model 3 were used. Probability was calculated by comparing the observed overlap value, expressed by the weighted E-distance (wE, see Fig. S11), with the distribution of 999 simulated overlap values for two community fractions with corresponding sizes and traits of only native (for ‘native vs. naturalized’ and ‘native vs. invasive’ comparisons) or naturalized species (for ‘naturalized vs. invasive’ comparison). For details, see Fig. S16.

# Appendix S6 – Results based on the PCoA axes from the Gower dissimilarity matrix

The following results were obtained by analysing the PCoA axes from the Gower dissimilarity matrix instead of the original traits. The data from the missForest imputation were used and all traits except the middle of the flowering period, which was considered an ordinal variable, were log10-transformed and scaled to zero mean and unit variance. Gower's (1971) distance with Podani's (1999) extension to ordinal variables was then used to calculate the species dissimilarity matrix for each vegetation plot. This matrix was then ordinated using the principal coordinate analysis (PCoA) and the resulting axes were used to calculate the distance of each community fraction from the center of the trait space (D) and their overlap (E-distance). Finally, the simulations of the null model 3 were used to determine whether the observed distances (D and E) are larger or smaller than expected for a group of species of the same size and traits of native species randomly selected from the habitat species pool. The results of both the unweighted (D and E) and weighted distance metrics (wD and wE; see Appendix S2) are shown.

**
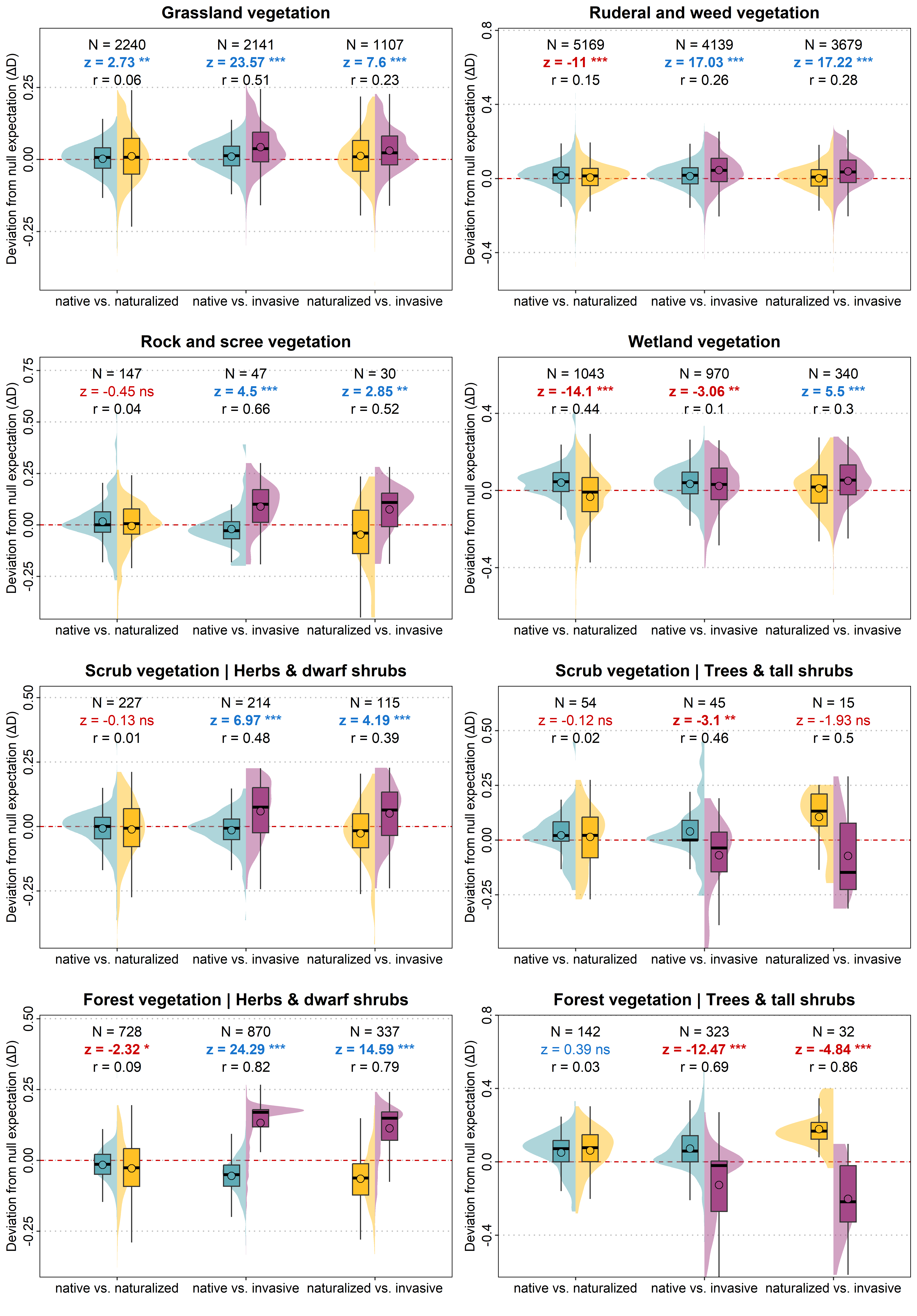
**

Fig. S20. Distances of native (cyan), naturalized (gold) and invasive (magenta) community fractions from the native center of the eight-dimensional trait space in each vegetation plot. The data from the missForest imputation and the simulations of the null model 3 were used. Distances are expressed as the deviation (ΔD) from the mean expected distance for community fractions of the same size simulated by the weighted random drawing of native species (or naturalized species for the ‘naturalized vs. invasive’ comparison) from the habitat species pools (null model 3). For details, see Fig. S15.


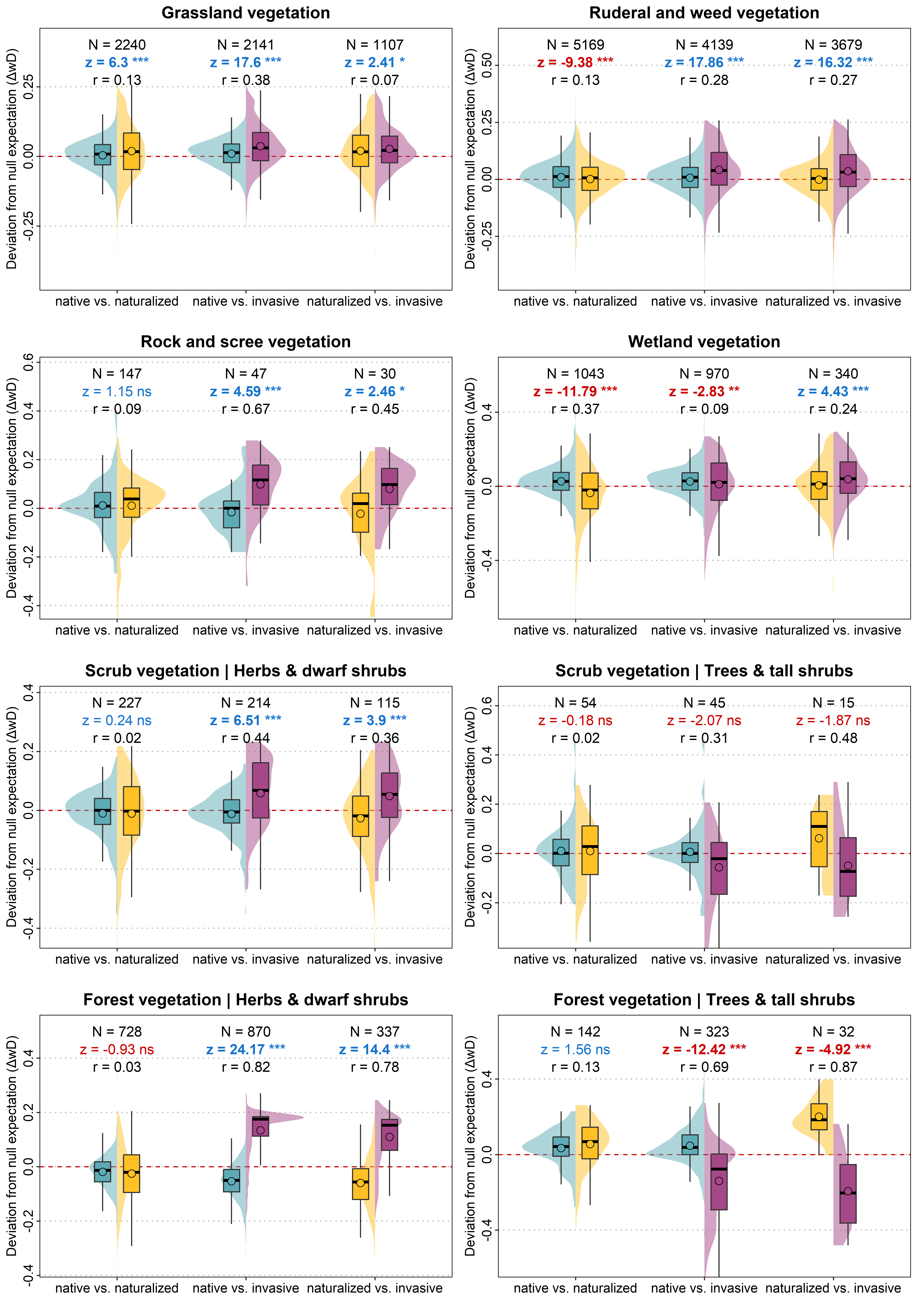


Fig. S21. Weighted distances of native (cyan), naturalized (gold) and invasive (magenta) community fractions from the native center of the eight-dimensional trait space in each vegetation plot. The data from the missForest imputation and the simulations of the null model 3 were used. Square-root-transformed species percentage covers in vegetation plots were used as weights to calculate the weighted mean distance of each community fraction from the weighted centroid of native species (wD; see Fig. S10). Distances are expressed as the deviation (ΔwD) from the mean expected distance for community fractions of the same size simulated by the weighted random drawing of native species (or naturalized species for the ‘naturalized vs. invasive’ comparison) from the habitat species pools (null model 3). For details, see Fig. S15.


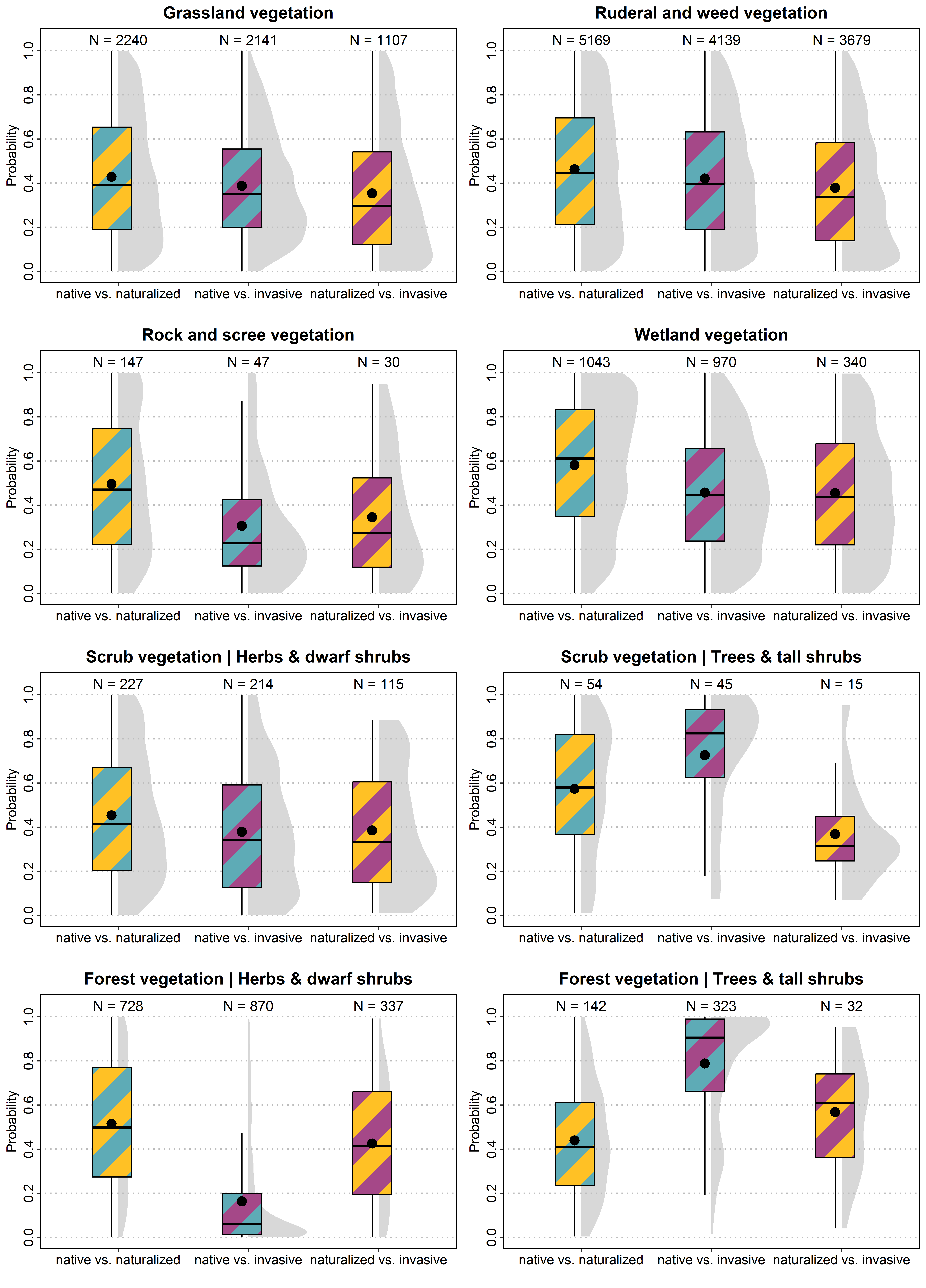


Fig. S22. Probability of overlap between native and naturalized species (cyan/gold), native and invasive species (cyan/magenta) and naturalized and invasive species (gold/magenta) in the eight-dimensional trait space of each plot. The data from the missForest imputation and the simulations of the null model 3 were used. Probability was calculated by comparing the observed overlap value, expressed by E-distance, with the distribution of 999 simulated overlap values for two community fractions with corresponding sizes and traits of only native (for ‘native vs naturalized’ and ‘native vs invasive’ comparisons) or naturalized species (for ‘naturalized vs invasive’ comparison). For details, see Fig S16.


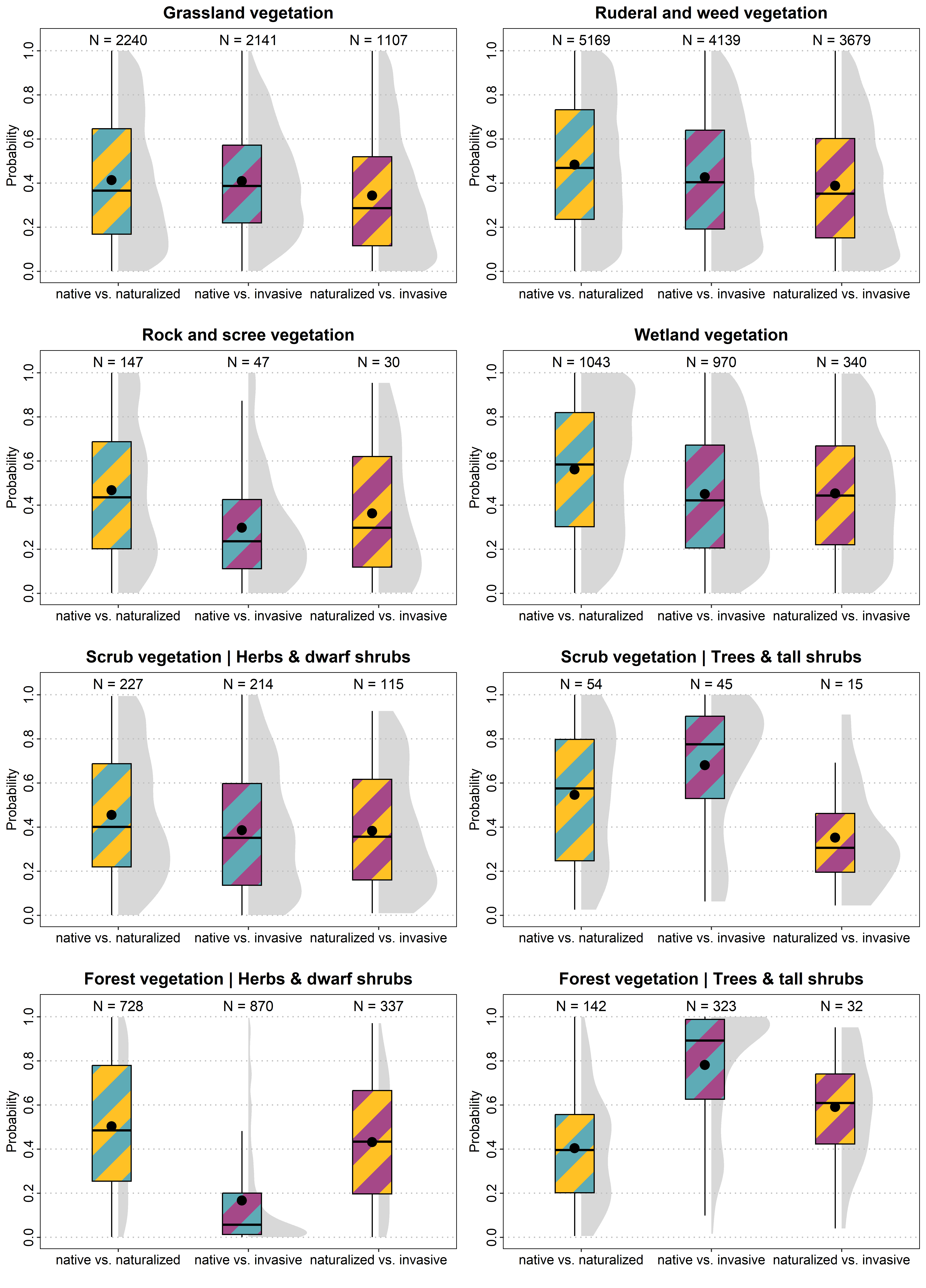


Fig. S23. Probability of overlap between native and naturalized species (cyan/gold), native and invasive species (cyan/magenta) and naturalized and invasive species (gold/magenta) in the eight-dimensional trait space of each plot. The data from the missForest imputation and the simulations of the null model 3 were used. Probability was calculated by comparing the observed overlap value, expressed by the weighted E-distance (wE, see Fig. S11), with the distribution of 999 simulated overlap values for two community fractions with corresponding sizes and traits of only native (for ‘native vs. naturalized’ and ‘native vs. invasive’ comparisons) or naturalized species (for ‘naturalized vs. invasive’ comparison). For details, see Fig. S16.

# Appendix S7 – Results based on the data from the MICE-PMM and Phylopars imputations

This appendix presents the results based on the data from the MICE-PMM and Phylopars imputations. The simulations of the null model 3 were used for this analysis. The results of both the unweighted (D and E) and weighted distance metrics (wD and wE; see Appendix S2) are shown.


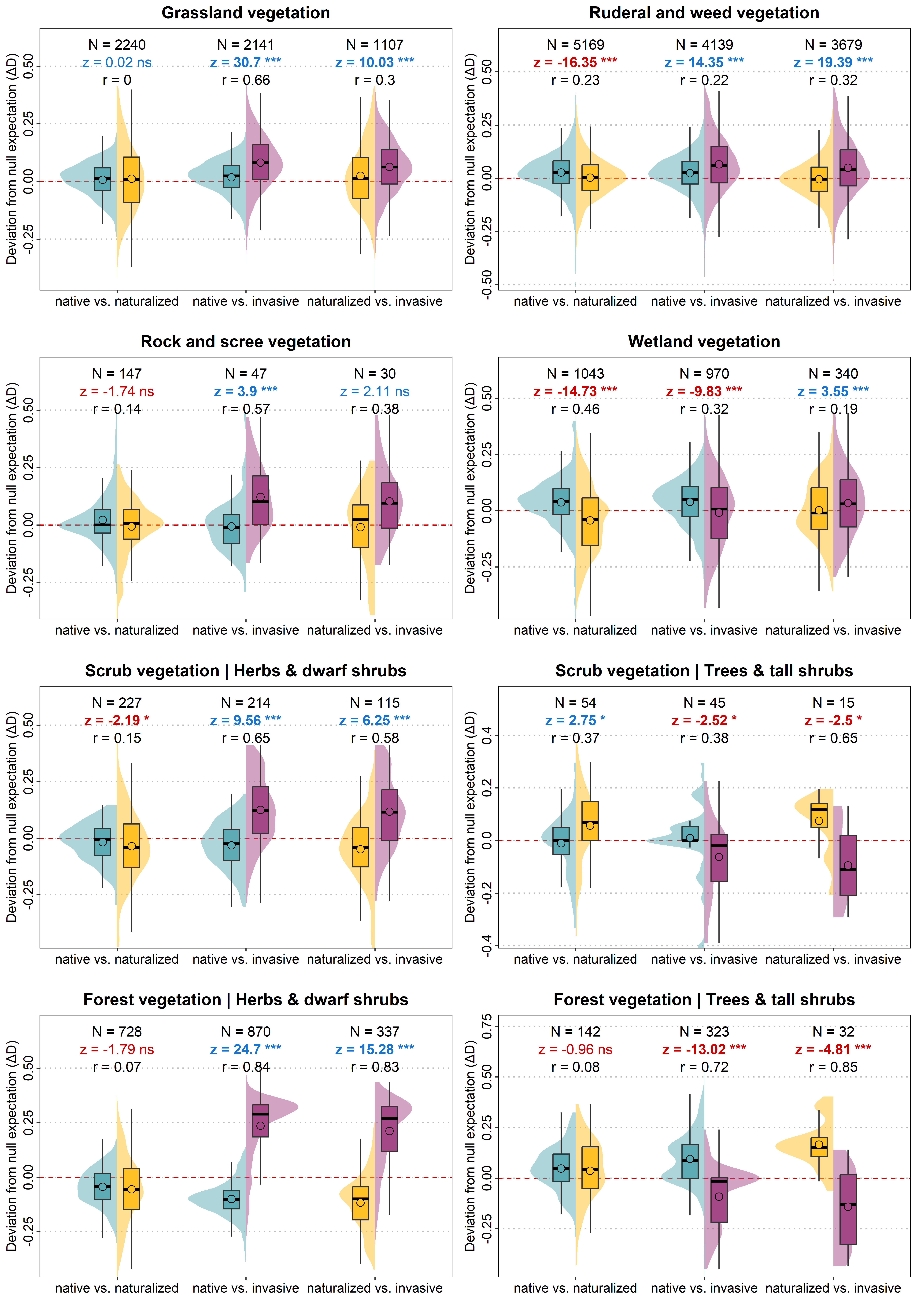


Fig. S24. Distances of native (cyan), naturalized (gold) and invasive (magenta) community fractions from the native center of the eight-dimensional trait space in each vegetation plot. The data from the MICE-PMM imputation and the simulations of the null model 3 were used. Distances are expressed as the deviation (ΔD) from the mean expected distance for community fractions of the same size simulated by the weighted random drawing of native species (or naturalized species for the ‘naturalized vs. invasive’ comparison) from the habitat species pools (null model 3). For details, see Fig S15.


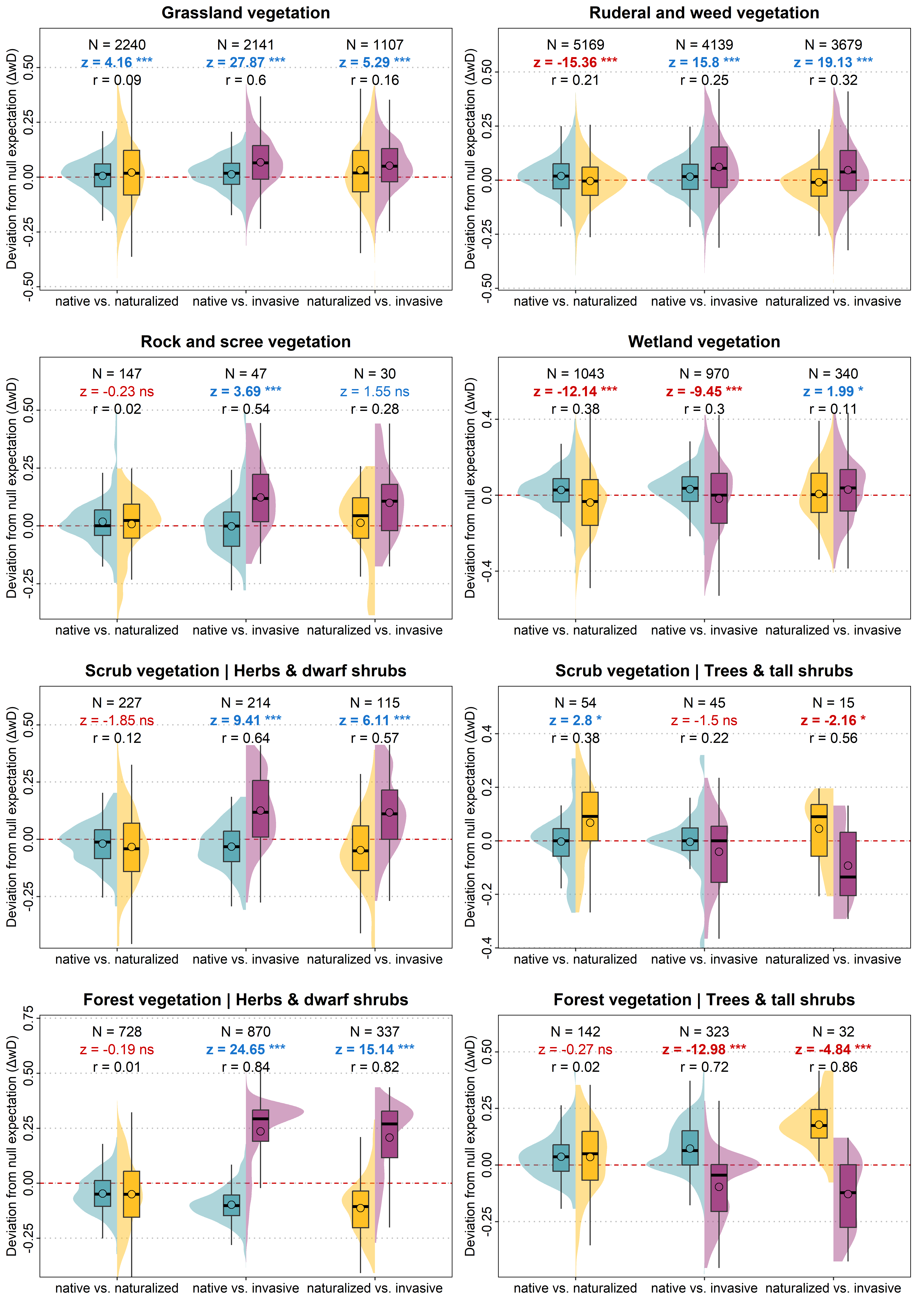


Fig. S25. Weighted distances of native (cyan), naturalized (gold) and invasive (magenta) community fractions from the native center of the eight-dimensional trait space in each vegetation plot. The data from the MICE-PMM imputation and the simulations of the null model 3 were used. Square-root-transformed species percentage covers in vegetation plots were used as weights to calculate the weighted mean distance of each community fraction from the weighted centroid of native species (wD; see Fig. S10). Distances are expressed as the deviation (ΔwD) from the mean expected distance for community fractions of the same size simulated by the weighted random drawing of native species (or naturalized species for the ‘naturalized vs. invasive’ comparison) from the habitat species pools (null model 3). For details, see Fig. S15.


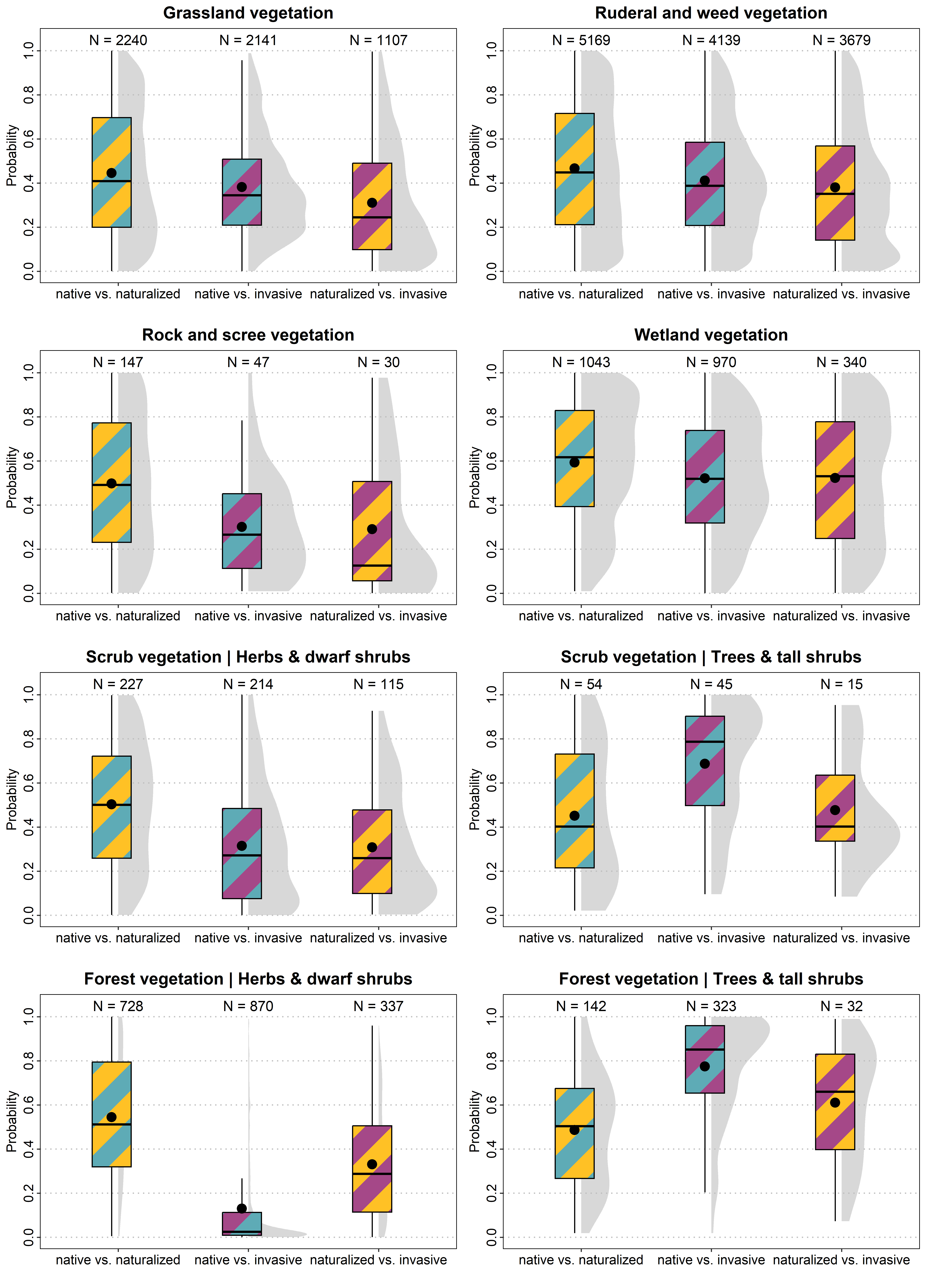


Fig. S26. Probability of overlap between native and naturalized species (cyan/gold), native and invasive species (cyan/magenta) and naturalized and invasive species (gold/magenta) in the eight-dimensional trait space of each plot. The data from the MICE-PMM imputation and the simulations of the null model 3 were used. Probability was calculated by comparing the observed overlap value, expressed by E-distance, with the distribution of 999 simulated overlap values for two community fractions with corresponding sizes and traits of only native (for ‘native vs naturalized’ and ‘native vs invasive’ comparisons) or naturalized species (for ‘naturalized vs invasive’ comparison). For details, see Fig. S16.


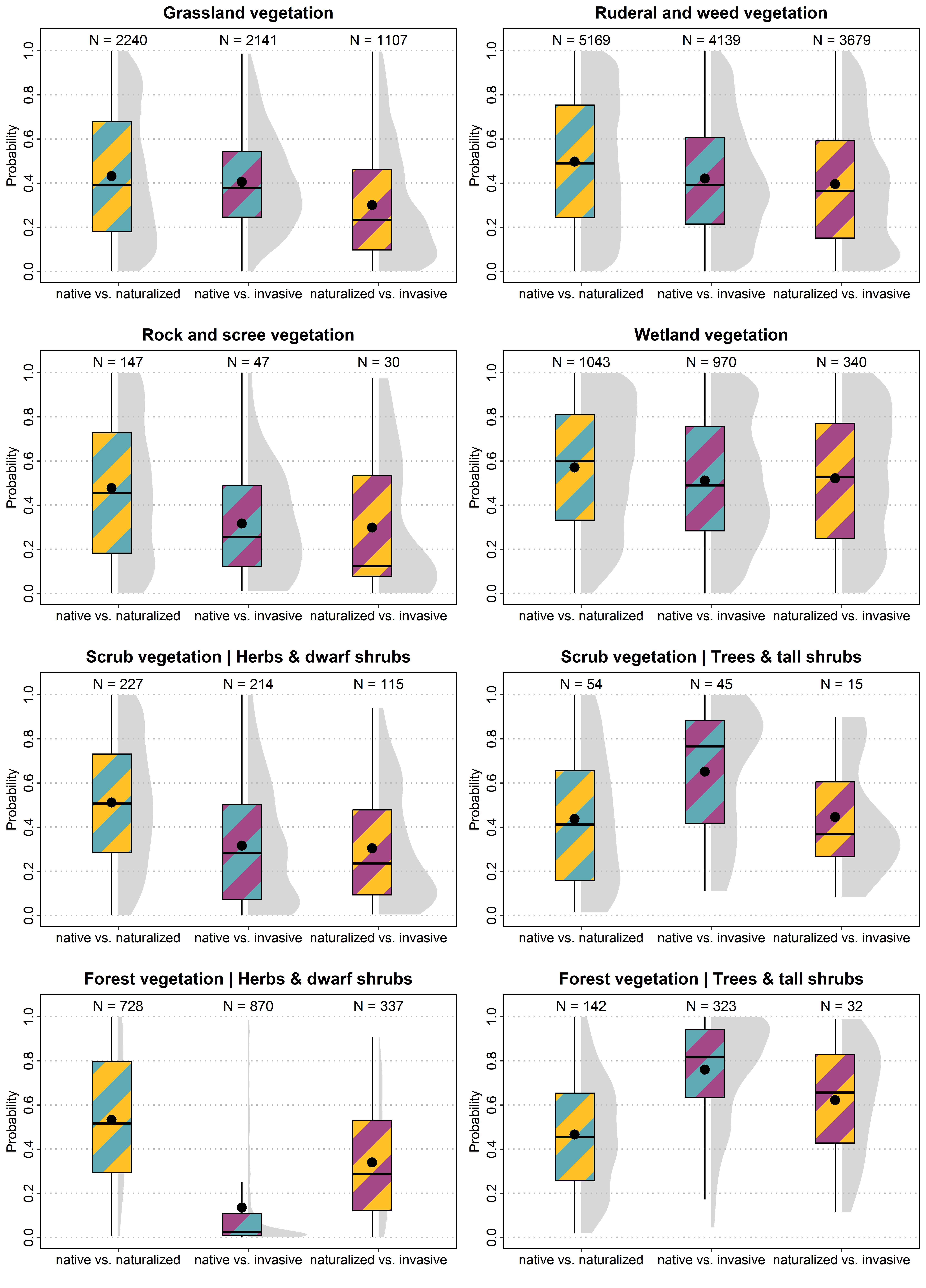


Fig. S27. Probability of overlap between native and naturalized species (cyan/gold), native and invasive species (cyan/magenta) and naturalized and invasive species (gold/magenta) in the eight-dimensional trait space of each plot. The data from the MICE-PMM imputation and the simulations of the null model 3 were used. Probability was calculated by comparing the observed overlap value, expressed by the weighted E-distance (wE, see Fig. S11), with the distribution of 999 simulated overlap values for two community fractions with corresponding sizes and traits of only native (for ‘native vs naturalized’ and ‘native vs invasive’ comparisons) or naturalized species (for ‘naturalized vs invasive’ comparison). For details, see Fig. S16.


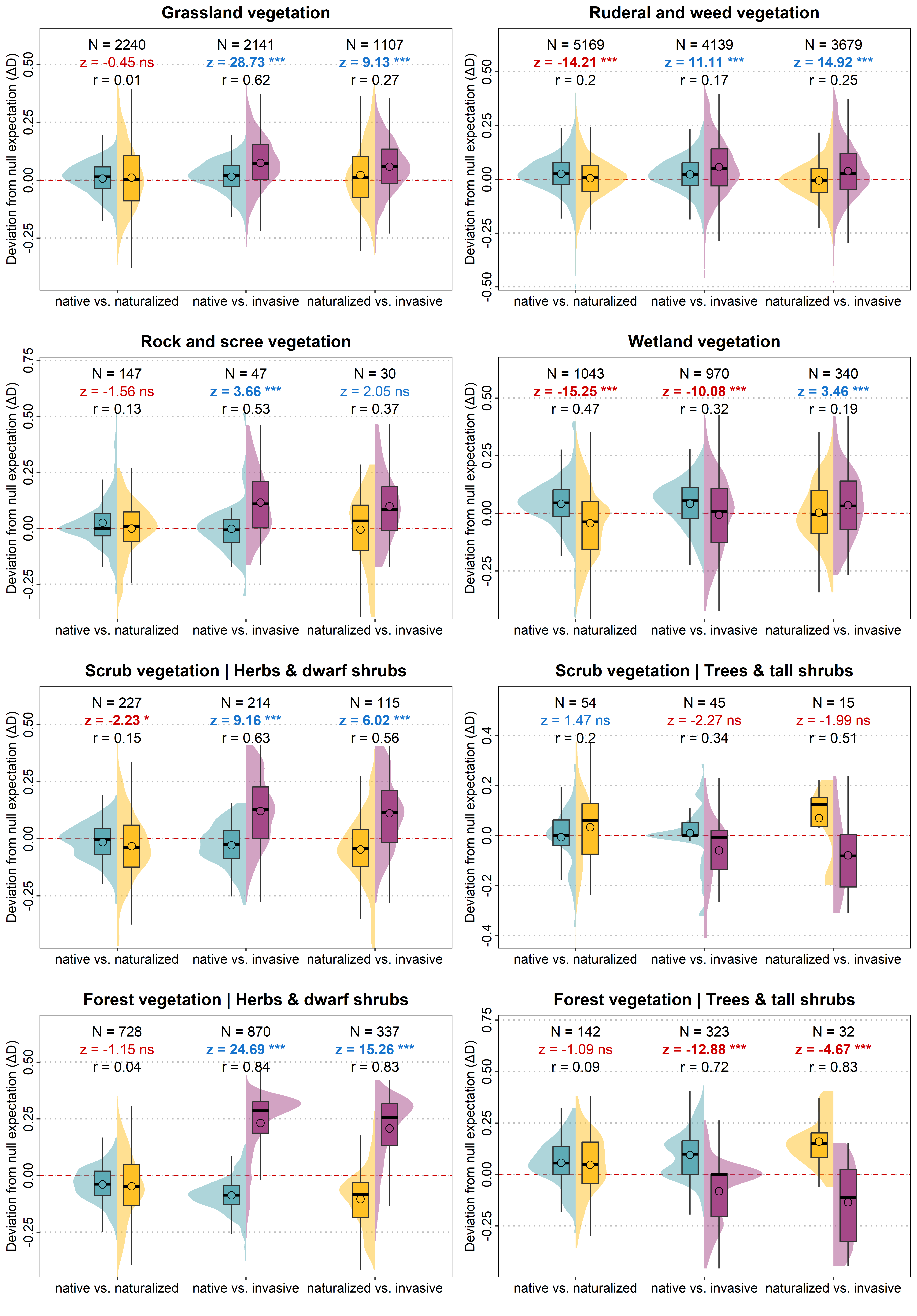


Fig. S28. Distances of native (cyan), naturalized (gold) and invasive (magenta) community fractions from the native center of the eight-dimensional trait space in each vegetation plot. The data from the Phylopars imputation and the simulations of the null model 3 were used. Distances are expressed as the deviation (ΔD) from the mean expected distance for community fractions of the same size simulated by the weighted random drawing of native species (or naturalized species for the ‘naturalized vs. invasive’ comparison) from the habitat species pools (null model 3). For details, see Fig. S15.


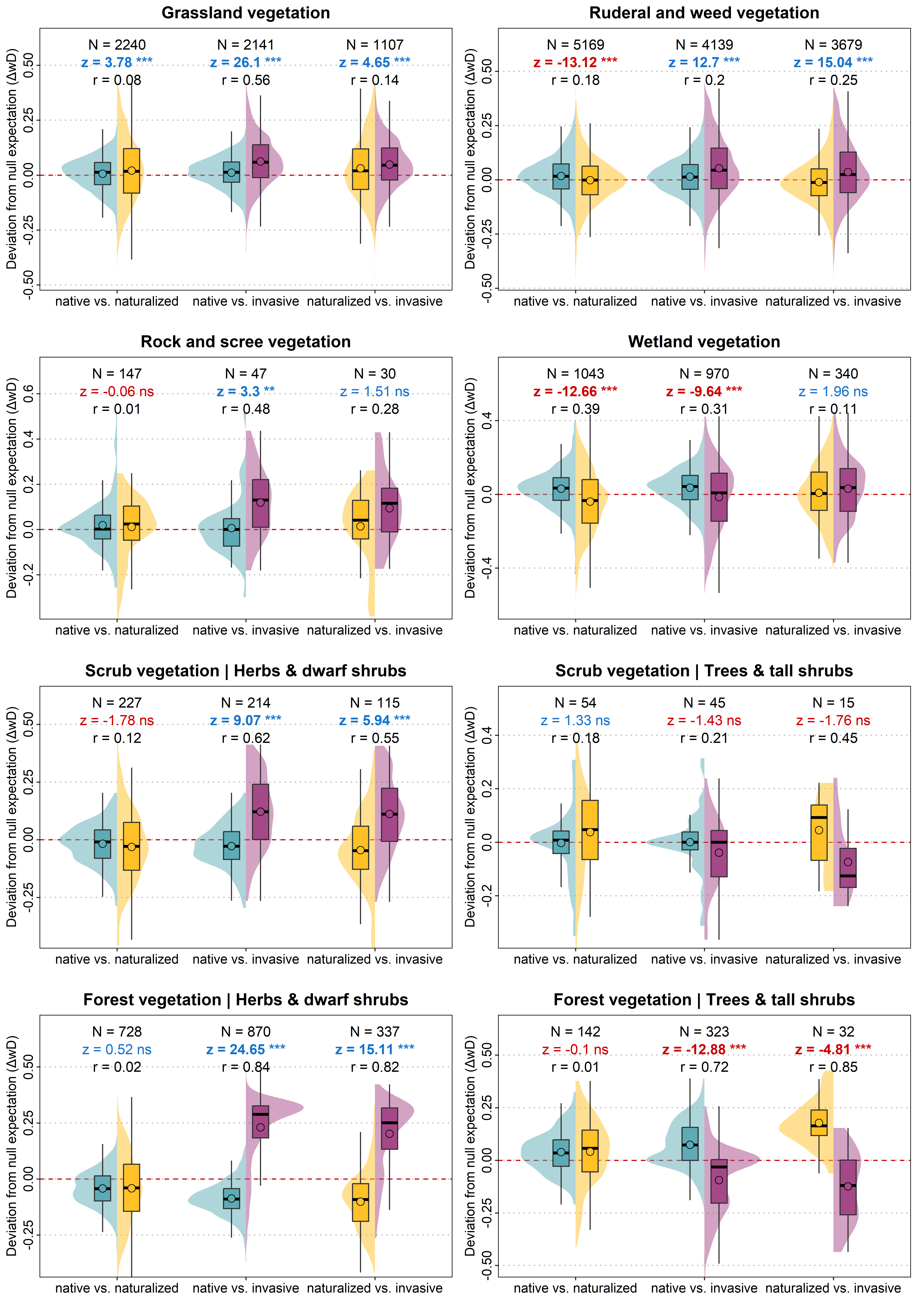


Fig. S29. Weighted distances of native (cyan), naturalized (gold) and invasive (magenta) community fractions from the native center of the eight-dimensional trait space in each vegetation plot. The data from the Phylopars imputation and the simulations of the null model 3 were used. Square-root-transformed species percentage covers in vegetation plots were used as weights to calculate the weighted mean distance of each community fraction from the weighted centroid of native species (wD; see Fig. S10). Distances are expressed as the deviation (ΔwD) from the mean expected distance for community fractions of the same size simulated by the weighted random drawing of native species (or naturalized species for the ‘naturalized vs. invasive’ comparison) from the habitat species pools (null model 3). For details, see Fig. S15.


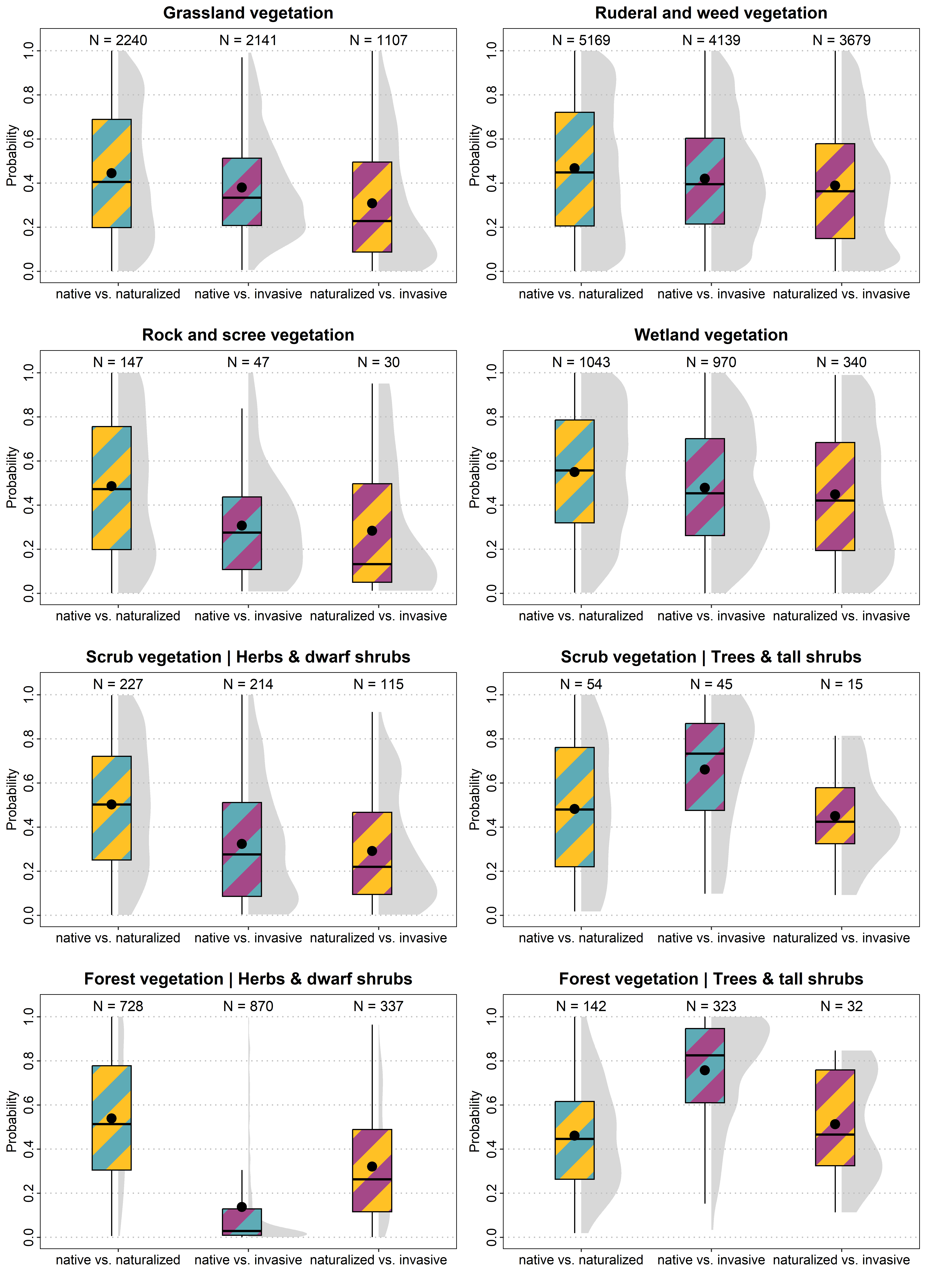


Fig. S30. Probability of overlap between native and naturalized species (cyan/gold), native and invasive species (cyan/magenta) and naturalized and invasive species (gold/magenta) in the eight-dimensional trait space of each plot. The data from the Phylopars imputation and the simulations of the null model 3 were used. Probability was calculated by comparing the observed overlap value, expressed by E-distance, with the distribution of 999 simulated overlap values for two community fractions with corresponding sizes and traits of only native (for ‘native vs naturalized’ and ‘native vs invasive’ comparisons) or naturalized species (for ‘naturalized vs invasive’ comparison). For details, see Fig. S16.


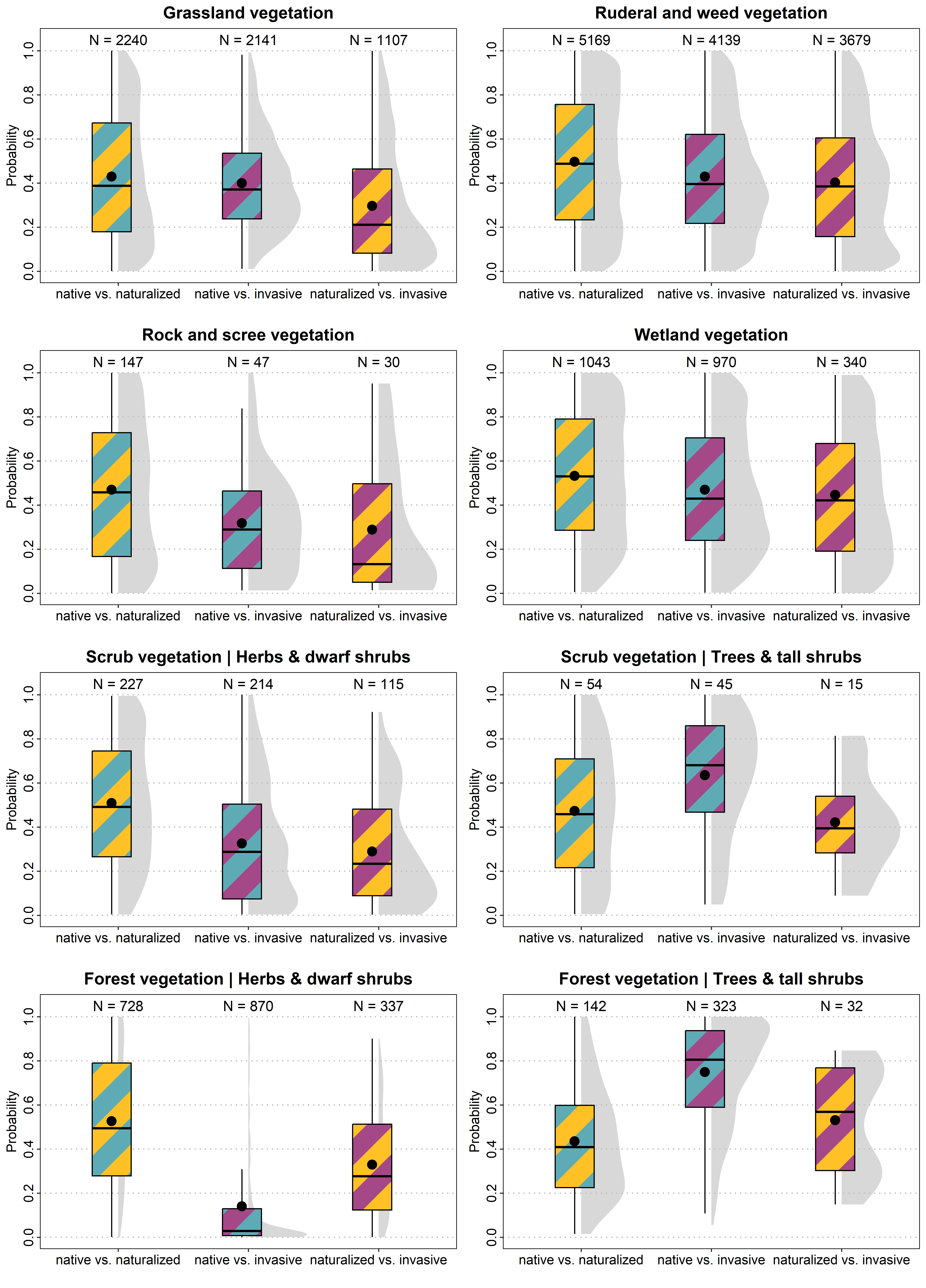


Fig. S31. Probability of overlap between native and naturalized species (cyan/gold), native and invasive species (cyan/magenta) and naturalized and invasive species (gold/magenta) in the eight-dimensional trait space of each plot. The data from the Phylopars imputation and the simulations of the null model 3 were used. Probability was calculated by comparing the observed overlap value, expressed by the weighted E-distance (wE, see Fig. S11), with the distribution of 999 simulated overlap values for two community fractions with corresponding sizes and traits of only native (for ‘native vs naturalized’ and ‘native vs invasive’ comparisons) or naturalized species (for ‘naturalized vs invasive’ comparison). For details, see Fig. S16.

# Appendix S8 – Results of the alternative null models

This appendix presents the results of analyses in which the simulations of the alternative null models 1 and 2 (see Methods) were used. Only the data from the missForest imputation were used for these analyses. The results of both the unweighted (D and E) and weighted distance metrics (wD and wE; see Appendix S2) are shown.


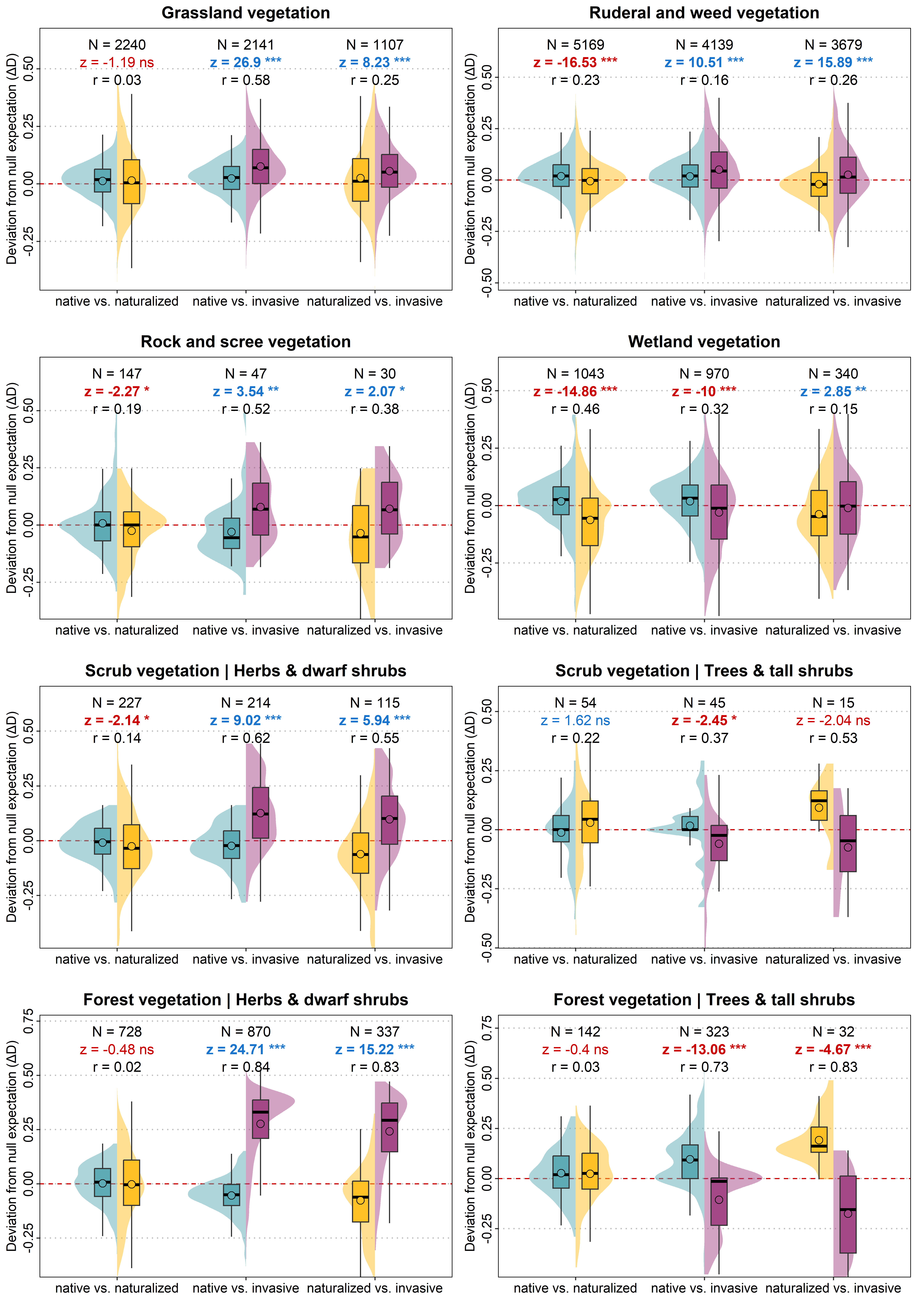


Fig. S32. Distances of native (cyan), naturalized (gold) and invasive (magenta) community fractions from the native center of the eight-dimensional trait space in each vegetation plot. The data from the missForest imputation and the simulations of the null model 1 were used. Distances are expressed as the deviation (ΔD) from the mean expected distance for community fractions of the same size simulated by the unweighted random drawing of native species (or naturalized species for the ‘naturalized vs. invasive’ comparison) from the habitat species pools (null model 1). For details, see Fig. S15.


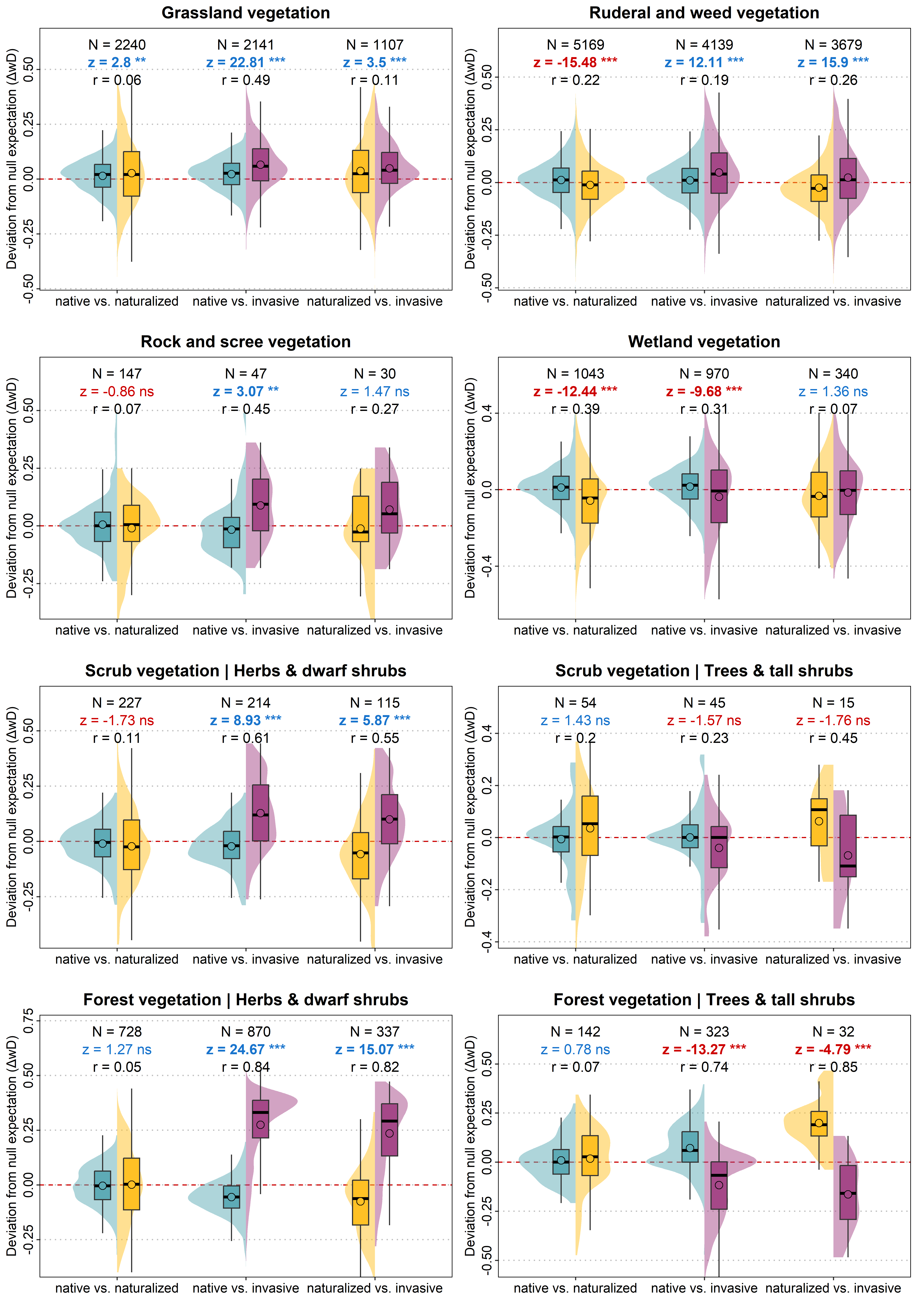


Fig. S33. Weighted distances of native (cyan), naturalized (gold) and invasive (magenta) community fractions from the native center of the eight-dimensional trait space in each vegetation plot. The data from the missForest imputation and the simulations of the null model 1 were used. Square-root-transformed species percentage covers in vegetation plots were used as weights to calculate the weighted mean distance of each community fraction from the weighted centroid of native species (wD; see Fig. S10). Distances are expressed as the deviation (ΔwD) from the mean expected distance for community fractions of the same size simulated by the unweighted random drawing of native species (or naturalized species for the ‘naturalized vs. invasive’ comparison) from the habitat species pools (null model 1). For details, see Fig. S15.


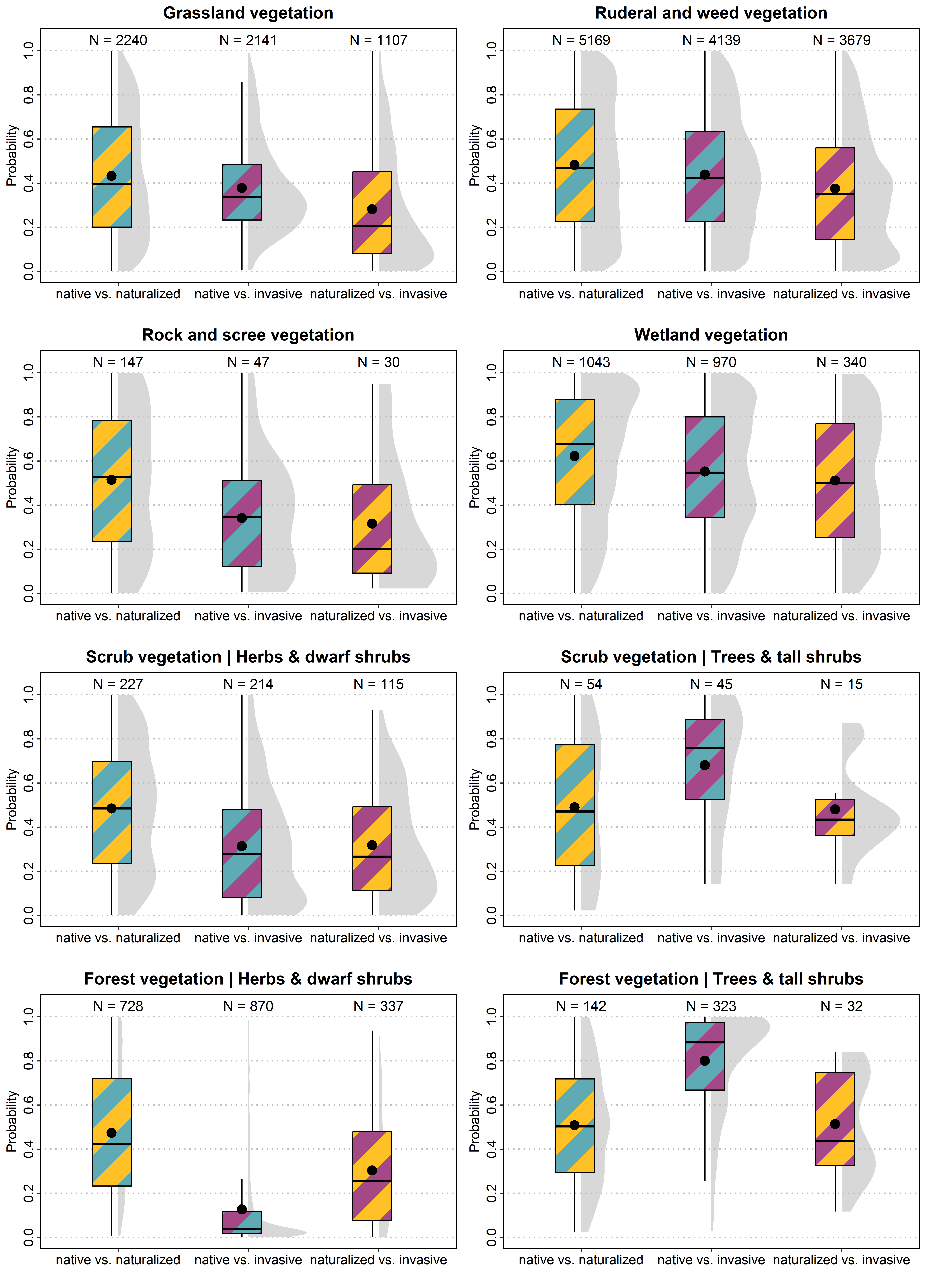


Fig. S34. Probability of overlap between native and naturalized species (cyan/gold), native and invasive species (cyan/magenta) and naturalized and invasive species (gold/magenta) in the eight-dimensional trait space of each plot. The data from the missForest imputation and the simulations of the null model 1 were used. Probability was calculated by comparing the observed overlap value, expressed by E-distance, with the distribution of 999 simulated overlap values for two community fractions with corresponding sizes and traits of only native (for ‘native vs naturalized’ and ‘native vs invasive’ comparisons) or naturalized species (for ‘naturalized vs invasive’ comparison). For details, see Fig. S16.


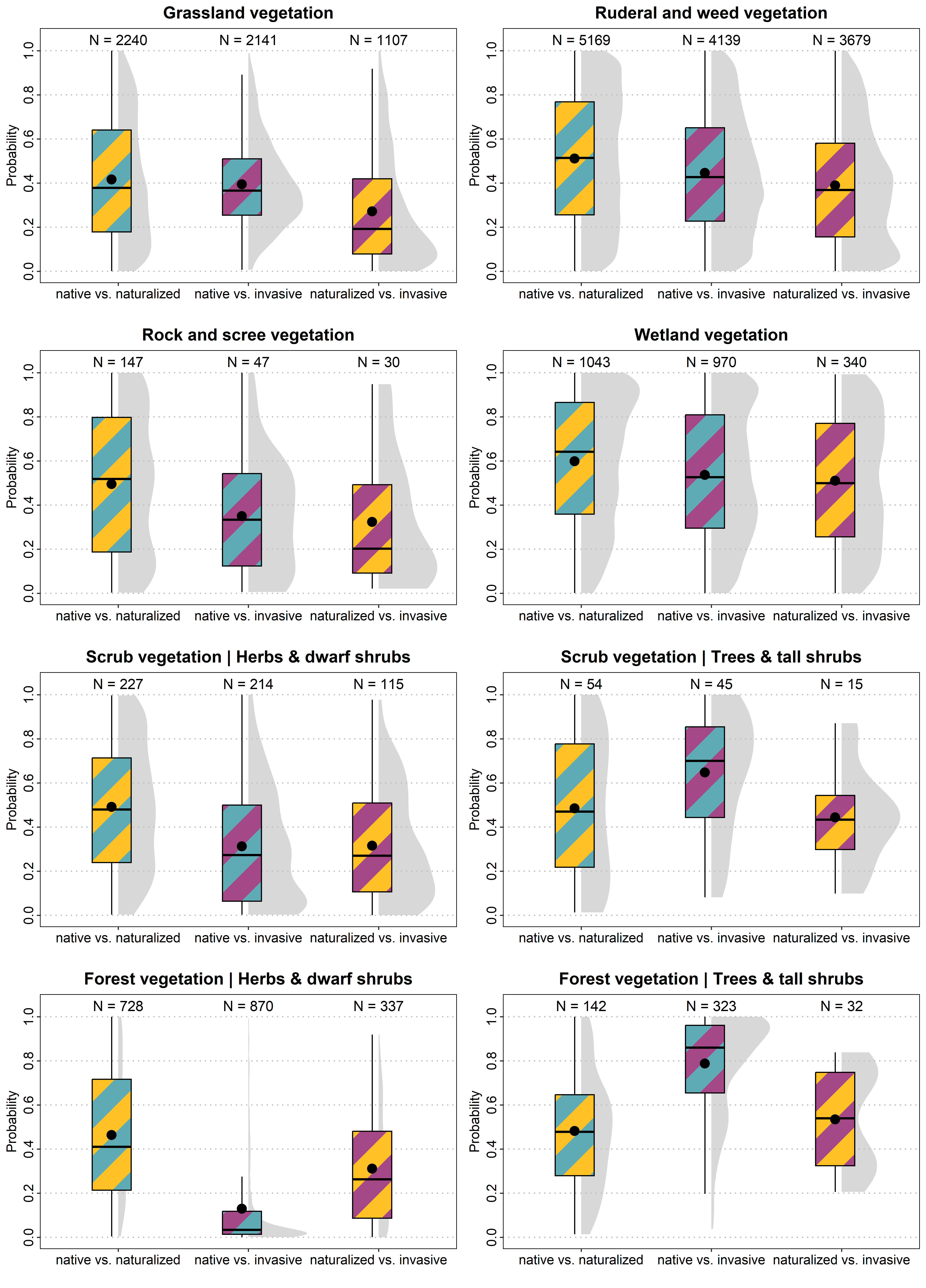


Fig. S35. Probability of overlap between native and naturalized species (cyan/gold), native and invasive species (cyan/magenta) and naturalized and invasive species (gold/magenta) in the eight-dimensional trait space of each plot. The data from the missForest imputation and the simulations of the null model 1 were used. Probability was calculated by comparing the observed overlap value, expressed by the weighted E-distance (wE, see Fig. S11), with the distribution of 999 simulated overlap values for two community fractions with corresponding sizes and traits of only native (for ‘native vs naturalized’ and ‘native vs invasive’ comparisons) or naturalized species (for ‘naturalized vs invasive’ comparison). For details, see Fig. S16.


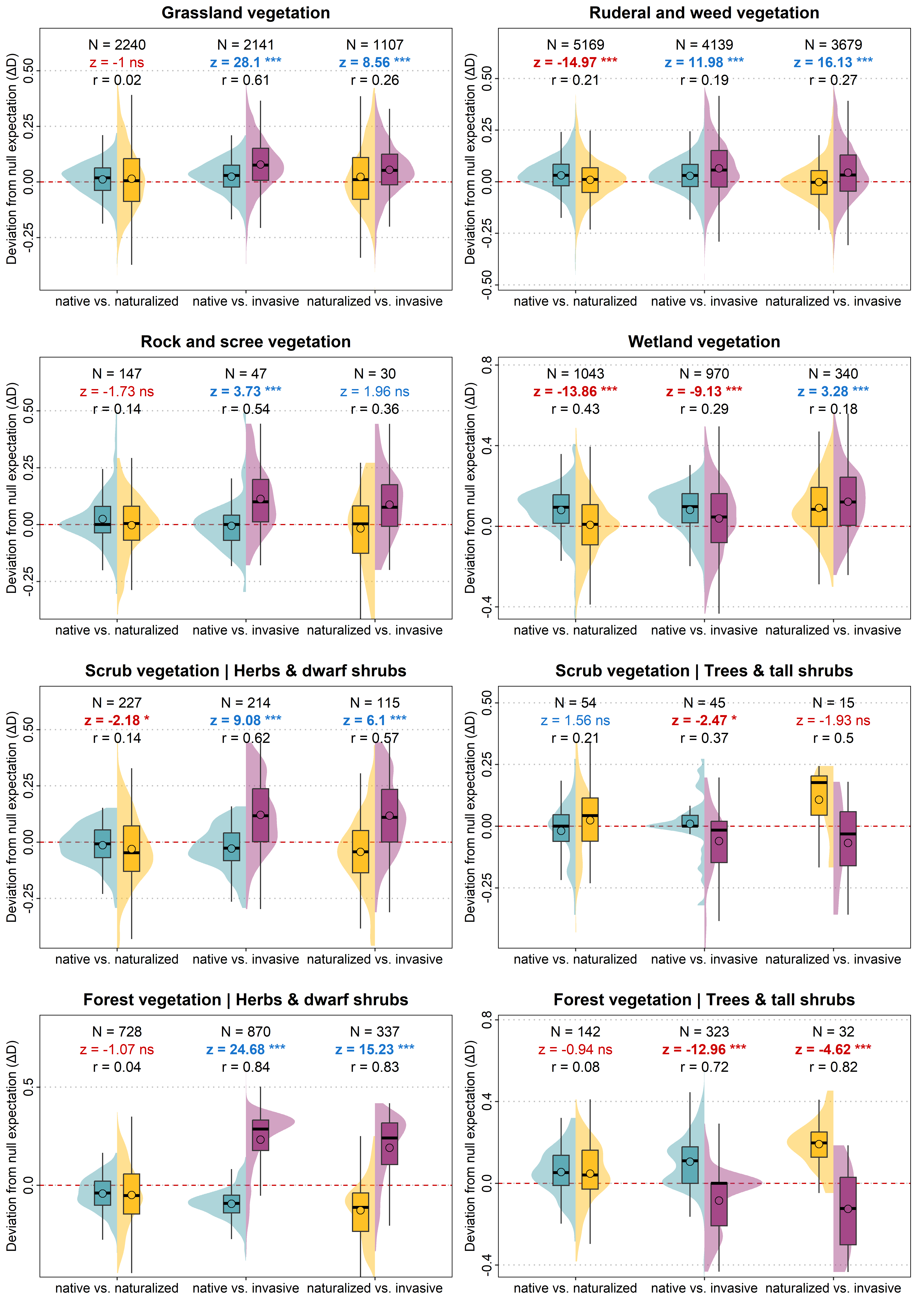


Fig. S36. Distances of native (cyan), naturalized (gold) and invasive (magenta) community fractions from the native center of the eight-dimensional trait space in each vegetation plot. The data from the missForest imputation and the simulations of the null model 2 were used. Distances are expressed as the deviation (ΔD) from the mean expected distance for community fractions of the same size simulated by the weighted random drawing of native species (or naturalized species for the ‘naturalized vs. invasive’ comparison) from the habitat species pools (null model 2). For details, see Fig. S15.


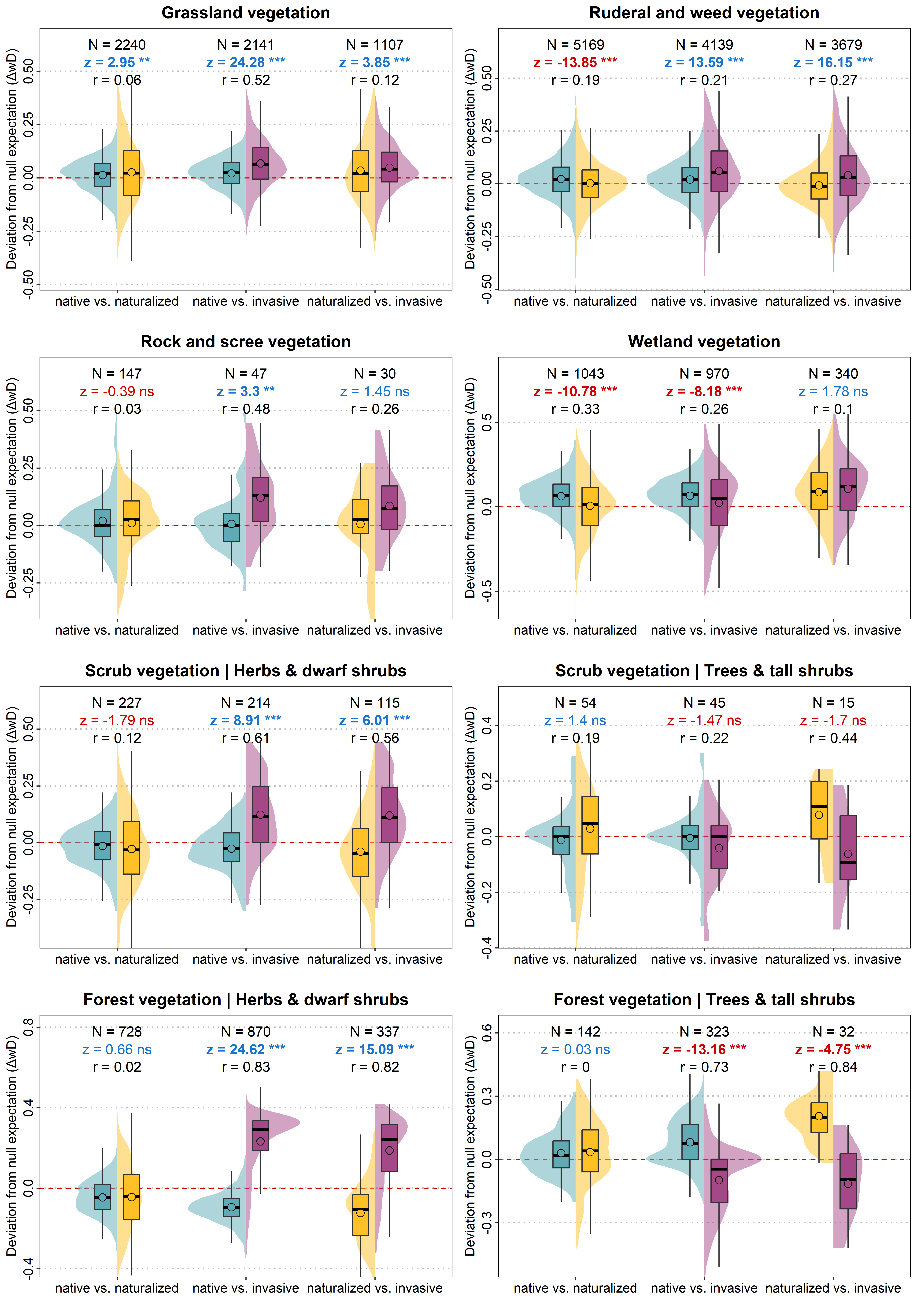


Fig. S37. Weighted distances of native (cyan), naturalized (gold) and invasive (magenta) community fractions from the native center of the eight-dimensional trait space in each vegetation plot. The data from the missForest imputation and the simulations of the null model 2 were used. Square-root-transformed species percentage covers in vegetation plots were used as weights to calculate the weighted mean distance of each community fraction from the weighted centroid of native species (wD; see Fig. S10). Distances are expressed as the deviation (ΔwD) from the mean expected distance for community fractions of the same size simulated by the weighted random drawing of native species (or naturalized species for the ‘naturalized vs. invasive’ comparison) from the habitat species pools (null model 2). For details, see Fig. S15.


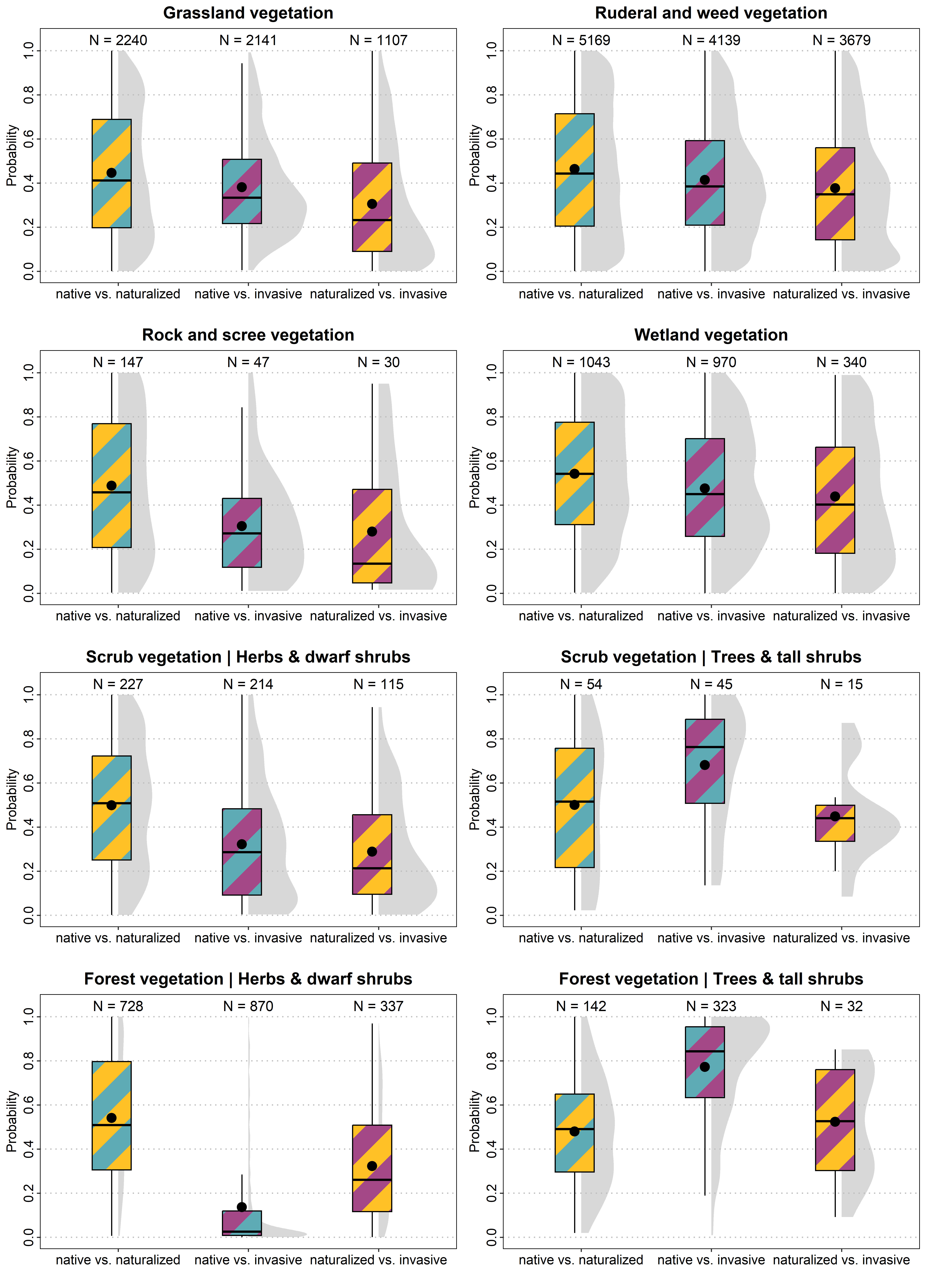


Fig. S38. Probability of overlap between native and naturalized species (cyan/gold), native and invasive species (cyan/magenta) and naturalized and invasive species (gold/magenta) in the eight-dimensional trait space of each plot. The data from the missForest imputation and the simulations of the null model 2 were used. Probability was calculated by comparing the observed overlap value, expressed by E-distance, with the distribution of 999 simulated overlap values for two community fractions with corresponding sizes and traits of only native (for ‘native vs naturalized’ and ‘native vs invasive’ comparisons) or naturalized species (for ‘naturalized vs invasive’ comparison). For details, see Fig. S16.


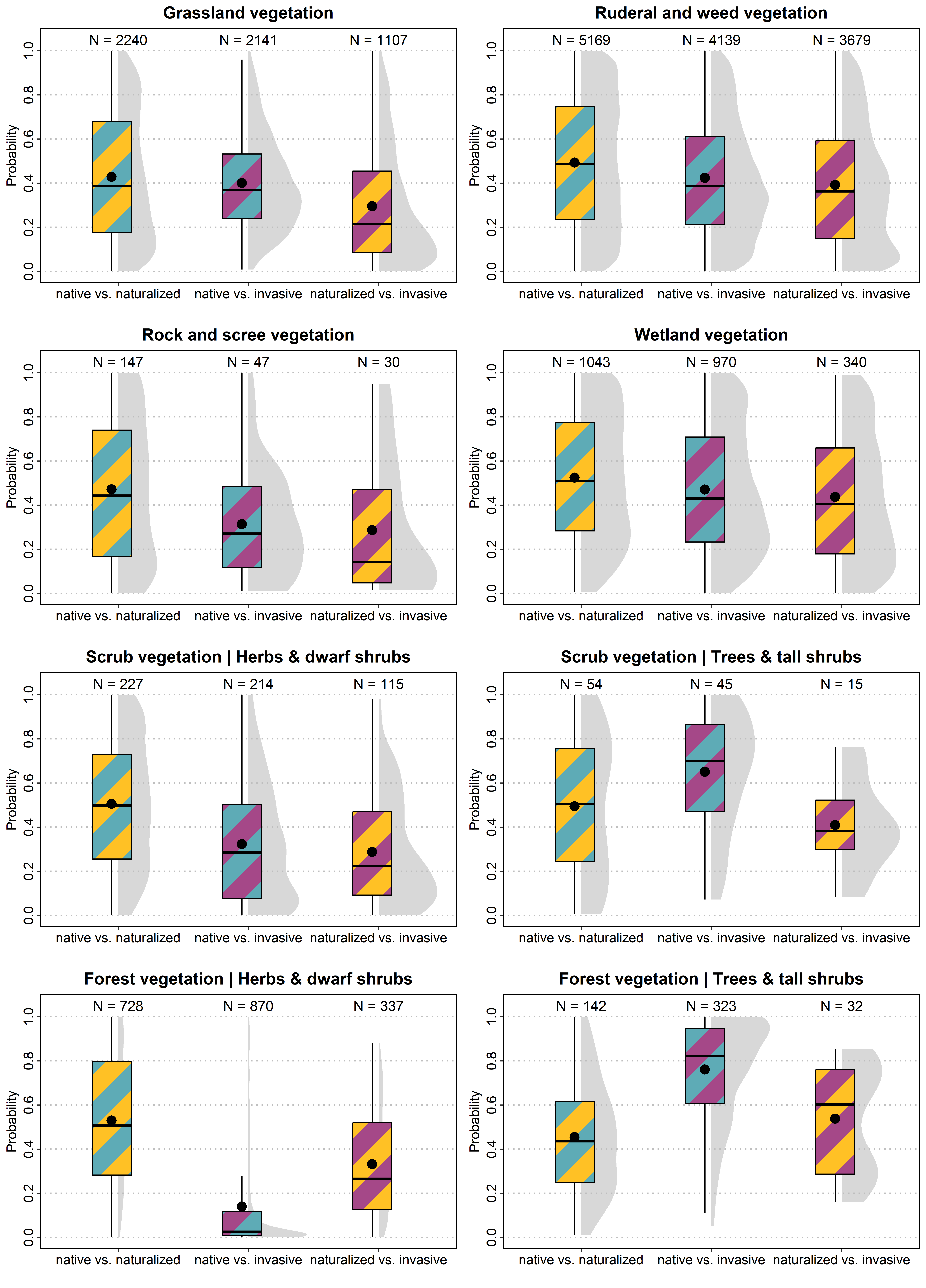


Fig. S39. Probability of overlap between native and naturalized species (cyan/gold), native and invasive species (cyan/magenta) and naturalized and invasive species (gold/magenta) in the eight-dimensional trait space of each plot. The data from the missForest imputation and the simulations of the null model 2 were used. Probability was calculated by comparing the observed overlap value, expressed by the weighted E-distance (wE, see Fig. S11), with the distribution of 999 simulated overlap values for two community fractions with corresponding sizes and traits of only native (for ‘native vs naturalized’ and ‘native vs invasive’ comparisons) or naturalized species (for ‘naturalized vs invasive’ comparison). For details, see Fig. S16.

# References

Divíšek, J., Chytrý, M., Beckage, B., Gotelli, N.J., Lososová, Z., Pyšek, P., *et al.* (2018). Similarity of introduced plant species to native ones facilitates naturalization, but differences enhance invasion success. *Nature Communications*, 9, 4631.

Gower, J.C. (1971). A general coefficient of similarity and some of its properties. *Biometrics*, 27, 857–871.

Jin, Y. & Qian, H. (2022). V.PhyloMaker2: An updated and enlarged R package that can generate very large phylogenies for vascular plants. *Plant Diversity*, 44, 335–339.

Laliberté, E. & Legendre, P. (2010). A distance-based framework for measuring functional diversity from multiple traits. *Ecology*, 91, 299–305.

Penone, C., Davidson, A.D., Shoemaker, K.T., Marco, M.D., Rondinini, C., Brooks, T.M., *et al.* (2014). Imputation of missing data in life-history trait datasets: which approach performs the best? *Methods in Ecology and Evolution*, 5, 961–970.

Peres-Neto, P.R. (2006). A unified strategy for estimating and controlling spatial, temporal and phylogenetic autocorrelation in ecological models. *Oecologia Brasiliensis*, 10, 105–119.

Podani, J. (1999). Extending Gower’s general coefficient of similarity to ordinal characters. *TAXON*, 48, 331–340.

Rizzo, M.L. & Székely, G.J. (2010). DISCO analysis: A nonparametric extension of analysis of variance. *The Annals of Applied Statistics*, 4, 1034–1055.

Rizzo, M.L. & Székely, G.J. (2016). Energy distance. *WIREs Computational Statistics*, 8, 27–38.
